# Non-invasive temporal interference electrical stimulation of the human hippocampus

**DOI:** 10.1038/s41593-023-01456-8

**Published:** 2023-10-19

**Authors:** Ines R. Violante, Ketevan Alania, Antonino M. Cassarà, Esra Neufeld, Emma Acerbo, Romain Carron, Adam Williamson, Danielle L. Kurtin, Edward Rhodes, Adam Hampshire, Niels Kuster, Edward S. Boyden, Alvaro Pascual-Leone, Nir Grossman

**Affiliations:** 1 School of Psychology, Faculty of Health and Medical Sciences, University of Surrey, Guildford, UK; 2 Department of Brain Sciences, Imperial College London, London, UK; 3 UK Dementia Research Institute, Imperial College London, London, UK; 4 Foundation for Research on Information Technologies in Society (IT’IS), Zurich, Switzerland; 5 Institut de Neurosciences des Systèmes, Aix-Marseille University, INSERM, Marseille, France; 6 Department of Neurology and Neurosurgery, Emory University Hospital, Atlanta, GA USA; 7 Department of Functional and Stereotactic Neurosurgery, Timone University Hospital, Marseille, France; 8 International Clinical Research Center, St. Anne’s University Hospital and Faculty of Medicine, Masaryk University, Brno, Czech Republic; 9 Department of Information Technology and Electrical Engineering, Swiss Federal Institute of Technology, Zurich, Switzerland; 10 Departments of Brain and Cognitive Sciences, Media Arts and Sciences, and Biological Engineering, McGovern and Koch Institutes, Massachusetts Institute of Technology, Cambridge, MA USA; 11 Howard Hughes Medical Institute, Cambridge, MA USA; 12 Hinda and Arthur Marcus Institute for Aging Research and Deanna and Sidney Wolk Center for Memory Health, Hebrew SeniorLife, Boston, MA USA; 13 Department of Neurology, Harvard Medical School, Boston, MA USA

**Keywords:** Medical research, Neuroscience, Biological techniques

## Abstract

Deep brain stimulation (DBS) via implanted electrodes is used worldwide to treat patients with severe neurological and psychiatric disorders. However, its invasiveness precludes widespread clinical use and deployment in research. Temporal interference (TI) is a strategy for non-invasive steerable DBS using multiple kHz-range electric fields with a difference frequency within the range of neural activity. Here we report the validation of the non-invasive DBS concept in humans. We used electric field modeling and measurements in a human cadaver to verify that the locus of the transcranial TI stimulation can be steerably focused in the hippocampus with minimal exposure to the overlying cortex. We then used functional magnetic resonance imaging and behavioral experiments to show that TI stimulation can focally modulate hippocampal activity and enhance the accuracy of episodic memories in healthy humans. Our results demonstrate targeted, non-invasive electrical stimulation of deep structures in the human brain.

## Abstract (web summary)

Electrical deep brain stimulation therapy is limited by the risks of inserting electrodes into the brain. Here the authors report non-invasive deep brain stimulation in the human hippocampus using temporal interference of kHz electric fields.

## Main

A multitude of brain disorders have debilitating impacts on quality of life, with neurological conditions increasingly recognized as major causes of death and disability, accounting for approximately 30% of the global burden of disease^1^. Most patients with brain disorders are unamenable to any form of pharmacological treatment^2,3^. Physical means of brain stimulation, known as ‘neuromodulation’, represent a tenable, nonpharmacological treatment strategy that acts through direct control of the aberrant neural activity underpinning the diseases or their symptomatic manifestation. Invasive electrical deep brain stimulation (DBS) has been used worldwide to treat patients with severe movement disorders, such as Parkinson’s disease^4^, and affective disorders, such as obsessive-compulsive disorder^5^. In addition, DBS is being investigated as a treatment for conditions such as depression^6,7^ and Alzheimer’s disease^8,9^. However, the risks associated with brain surgery make exploration of different brain targets difficult and limit DBS’s potential therapeutic impact^4,10^.

Non-invasive stimulation methods, such as transcranial magnetic stimulation and transcranial electrical stimulation (tES), have been used in many human clinical investigations^11,12^. However, their ability to directly stimulate deeper brain structures is achieved at the expense of inducing stronger stimulation of the overlying cortical areas, resulting in unanticipated side effects that can approach the limits of safety guidelines^13^.

**Fig. 1:**
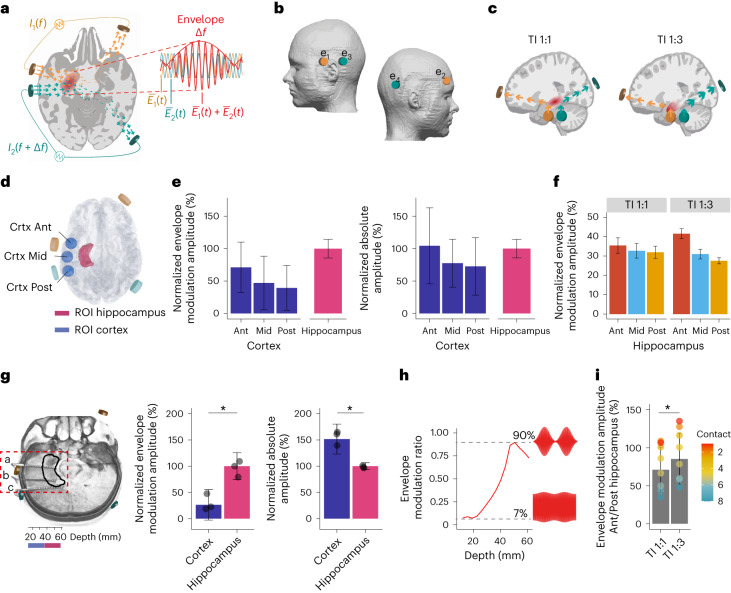
Fundamentals of TI hippocampal stimulation and validation using computational modeling and cadaver measurements. Concept of TI hippocampal stimulation: **a**, Two current sources *I*_1_ and *I*_2_ are applied simultaneously via electrically isolated pairs of scalp electrodes (orange and green) at kHz frequencies *f*_1_ and *f*_2_, with a small frequency difference Δ*f* = *f*_1_ − *f*_2_ within the range of neural activity. The currents generate oscillating electric fields $\bf E$_1_(*t*) and $\bf E$_2_(*t*) inside the brain (orange and green arrows, respectively). Superposition of these fields, $\bf E$_1_(*t*) + $\bf E$_2_(*t*), results in an envelope amplitude that is modulated periodically at Δ*f*. The peak amplitude of the envelope modulation can be localized in deep brain structures such as the hippocampus (highlighted in red). **b**, Schematic of electrode configuration targeting the left hippocampus. Electrodes e_1_ and e_2_ formed one electrode pair (orange) and electrodes e_3_ and e_4_ another (green), corresponding to *I*_1_ and *I*_2_ in **a**. e_1_ and e_3_ were located at nasion plane of the left hemisphere, symmetrically above the anterior–posterior midline of the hippocampus (5 cm distance between electrode centers). e_2_ and e_4_ were located at a plane above the eyebrow on the right hemisphere (approximately 16 cm distance between electrode centers). Electrodes were 1.5 cm × 1.5 cm square with rounded corners for ex vivo and in vivo experiments and circular 2 cm diameter for computational modeling. **c**, Illustration of steering of the TI stimulation locus along the hippocampal longitudinal axis. TI stimulation with 1:1 current ratio (‘TI 1:1’) and stimulation locus in the middle region (left); TI stimulation with 1:3 current ratio (‘TI 1:3’) and locus in the anterior region (right). By reducing the current amplitude in one electrode pair and increasing it in the second while keeping the current sum fixed, the stimulation locus can be steered toward the electrode pair with the smaller current amplitude^14^. Computation of TI stimulation locus in a human anatomical model: **d**, Schematic of the ROIs in the left (stimulated) hippocampus and its overlying cortex; Ant, anterior; Mid, middle; Post, posterior. **e**, Left: fields’ envelope modulation amplitude. Right: fields’ absolute amplitude; for the ROIs shown in **d**. Values are median ± s.d. normalized to the hippocampal value here and thereafter (*n* indicates number of voxels (nvox) per ROI: Cortex (Crtx), Crtx Ant 48,103, Crtx Mid 43,247, Crtx Post 42,656, Hippocampus 50,349). For whole-brain electric field modeling, see Supplementary Fig. 1a. Note that the cortex ROIs are more heterogeneous than the hippocampus as these include gray matter with different folding and white matter tissue. **f**, Envelope modulation amplitude in hippocampal ROIs (for ROI schematic, see Fig. 2b) during TI 1:1 and TI 1:3 stimulations (*n* indicates nvox: Ant 22,651, Mid 17,718, Post 9,980); for additional current ratios, see Supplementary Fig. 1. Measurement of TI stimulation locus in a human cadaver (*I*_1_ = 2 kHz, 1 mA; *I*_2_ = 2.005 kHz, 1 mA): **g**, Left: CT head image with intracranial electrode leads a, b and c implanted in the left mesial temporal lobe. Each electrode consisted of 15 electrode contacts; black contour, approximate location of the left hippocampus; orange and green stimulation electrodes. Middle: amplitudes of the envelope modulation in the left (stimulated) hippocampus and its overlying cortex showing higher envelope amplitude at the hippocampus (LMM, two-sided paired *t*-test, *t*_(2)_ = −5.515, *P* = 0.0345, *n* = 3 electrodes). Right: absolute amplitudes in the left hippocampus and overlying cortex, showing higher absolute amplitude in the overlying cortex (LMM, two-sided paired *t*-test, *t*_(2)_ = 7.051, *P* = 0.0195). Dots represent individual electrodes. See Supplementary Table 1 for full statistics and Supplementary Fig. 2 for additional amplitude maps. **h**, Envelope modulation ratio versus depth for electrode b, showing increasing envelope modulation with depth. **i**, Anterior (Ant) to posterior (Post) envelope modulation amplitude for the TI 1:1 and TI 1:3 conditions, showing higher Ant/Post amplitudes for the TI 1:3 condition (two-sided paired *t*-test, *t*_(7)_ = −7.765, *P* = 1.204 × 10^−4^, *n* = 8 hippocampal electrode contacts); envelope modulation amplitudes in the anterior electrode a were normalized to the posterior electrode c. Dots represent individual contacts in the hippocampal region and are color coded by depth (cold colors for more superficial contacts and warmer colors for deeper contacts). Asterisks identify significant differences, *P* < 0.05. Bar plots show median ± s.d.

We recently reported a strategy for sculpting electrical fields to enable focused yet non-invasive neural stimulation at depth^14,15^. The strategy is based on delivering multiple electric fields to the brain at different kHz frequencies, which are too high to drive effective neural firing. The envelope amplitude of the combined field is modulated at the difference frequency, between the kHz fields, and set low enough to drive neural activity. Neural stimulation will occur at the targeted region, at the difference frequency, where the amplitude of the electric field envelope modulation is larger (Fig. 1a). We call this strategy temporal interference (TI) stimulation since the interference of multiple electric fields enables its focality. Since the magnitude of the envelope modulation depends on the relative amplitude and orientation of the applied electric fields, the stimulation locus (that is, the envelope modulation peak) can be focused remotely from the electrodes. The stimulation locus can also be steered toward one electrode pair by reducing its relative amplitude (the envelope modulation is typically proportional to the field with the lower amplitude)^14^.

In this Article, we aim to test the translation of these results by investigating the application of TI to the human hippocampus. Earlier human studies tested TI stimulation of cortical structures^16–18^, but the crucial non-invasive DBS capability has not been validated so far. We first focused on validating the locus of TI stimulation using computational modeling and cadaver measurements. We followed this by performing simultaneous TI and functional magnetic resonance imaging (fMRI) experiments designed to explore physiological changes in brain activity in response to stimulation and provide evidence for target engagement. Finally, we tested the behavioral impact of delivering TI stimulation to the hippocampus in healthy participants. We demonstrate the safety and tolerability of TI stimulation in humans, the ability to focally target the stimulation locus to the hippocampus, and the capacity to modulate hippocampal activity and behavioral performance.

## Results

### Validation of hippocampal targeting

We first examined whether the locus of TI stimulation can be localized to the hippocampus with minimal exposure of the overlying cortex. We positioned two pairs of electrodes on the scalp in a configuration that targets the left hippocampus (Fig. 1b) and computed the field distribution in the established anatomical MIDA model^19^. The model distinguishes a large number of tissue classes derived from high-resolution multi-modal magnetic resonance imaging (MRI) data, and accounts for electrical conductivity anisotropy and neural orientation based on diffusion tensor imaging (DTI). We applied two sinusoidal currents at 2.005 kHz and 2 kHz (resulting in a Δ*f* envelope frequency of 5 Hz) and an equal amplitude of 1 mA (peak to baseline, current density ~0.314 mA cm^−^^2^), that is, TI with 1:1 current ratio (‘TI 1:1’; Fig. 1c), and computed the fields’ envelope modulation amplitude and absolute amplitude along the (DTI-derived) principal fibers axis (Supplementary Fig. 1). We extracted these fields in regions of interest (ROIs) at the left, that is stimulated, hippocampus (‘Hipp’) and the overlying cortical regions, both underneath (anterior, ‘Crtx Ant’; posterior, ‘Crtx Post’) and between (middle, ‘Crtx Mid’) the stimulation electrodes (Fig. 1d). The fields’ envelope modulation amplitude in the hippocampus was 30–60% larger than in the overlying cortical regions (Hipp, 0.26 ± 0.04 V m^−1^ median ± s.d.; Crtx Ant, 0.18 ± 0.10; Crtx Mid, 0.12 ± 0.11; Crtx Post, 0.10 ± 0.09; Fig. 1e, left). In contrast, the fields’ absolute amplitude in the hippocampus was smaller than in the overlying cortical region underneath the Ant electrode (Hipp, 0.29 ± 0.04 V m^−1^; Crtx Ant, 0.30 ± 0.17; Crtx Mid, 0.23 ± 0.11; Crtx Post, 0.21 ± 0.13; Fig. 1e, right).

Given the distinctive functional organization along the hippocampal longitudinal axis^20^, we next explored the relative distribution of the TI electric fields between the ‘Ant’, ‘Mid’ and ‘Post’ regions of the hippocampus. The model showed similar envelope modulation amplitudes across hippocampal regions relative to total hippocampal exposure (Fig. 1f). Since the anterior hippocampus has been explicitly implicated in successful associative encoding^21^, we explored whether the locus of the TI electric fields can be steered anteriorly. We found that reducing the current in the anterior electrode pair e_1_–e_2_ to 0.5 mA (~0.225 mA cm^−^^2^) and increasing the amplitude in the posterior electrode pair e_3_–e_4_ to 1.5 mA (~0.675 mA cm^−^^2^), that is, TI with 1:3 current ratio (‘TI 1:3’; Fig. 1c), could increase the relative envelope modulation amplitude in the Ant hippocampal region (Fig. 1f; for additional current ratios, see Supplementary Fig. 1).

To validate that the locus of TI stimulation could indeed be targeted to the hippocampus, we applied the same electrode configuration and sinusoidal currents (Fig. 1c, TI 1:1) to a human cadaver and measured the electrical potential using intracranial electrodes implanted in the left mesial temporal lobe, perpendicular to the hippocampus long axis (Fig. 1g). Consistent with our modeling, the normalized envelope modulation amplitude was ~75% larger in the hippocampus compared to the overlying cortex (Fig. 1g and Supplementary Table 1). The largest electric field’s envelope modulation amplitude along the recording electrode b, between the two stimulation electrodes e_1_ and e_2_ (that is, a field direction perpendicular to the hippocampal longitudinal axis), was ~0.1 V m^−1^ at a depth of ~44 mm (consistent with the location of the hippocampus^22^) per 1 mA applied current (current density ~0.45 mA cm^−^^2^). The envelope modulation ratio along electrode b was low at the cortex (~7% at 12 mm depth) and high near the hippocampus (~90% at 50 mm depth; Fig. 1h). In contrast, the absolute amplitude was largest in the overlying cortical region, the normalized amplitude was ~50% larger in the cortex compared to the underlying hippocampus (Supplementary Table 1). The distribution of the absolute amplitude was similar when we applied two sinusoids at the Δ*f* frequency of 5 Hz (Supplementary Fig. 2). Changing the current ratio to 1:3 (Fig. 1c, TI 1:3) resulted in a larger envelope modulation amplitude in the Ant hippocampal region relative to the Post hippocampal region (*t*_(7)_ = −7.765, *P* < 0.001; Fig. 1i).

### Probing the physiological effect of TI stimulation

**Fig. 2:**
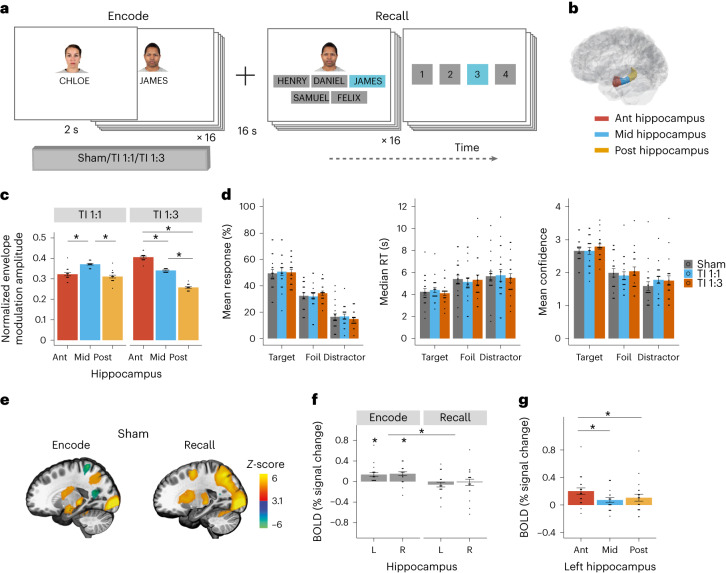
Experimental design, BOLD signal during sham and hippocampal fields. **a**, The face–name task was composed of nine blocks of encoding and recall. Each block contained 16 unique face^67^–name pairs followed by a delay and a recall period, where participants tried to select the correct name for each face out of five options (that is, one target, two foil names that were present in the block but associated with a different face, and two distractor names that were not present during the task). After each name selection, participants were asked to rate their choice confidence (1 (not confident at all) to 4 (extremely confident)). **b**, Schematic of the Ant, Mid and Post ROIs along the hippocampal longitudinal axis. **c**, Participants’ envelope modulation amplitude in hippocampal ROIs during TI 1:1 and TI 1:3 stimulations, computed with individualized MRI-based anatomical models; *n* = 16 participants (Supplementary Fig. 3). Showing a steering of the envelope amplitude peak from Mid hippocampal ROI during TI 1:1 stimulation (LMM, *F*_(2,30)_ = 26.05, *P* = 2.7 × 10^−7^; post hoc comparisons using the Tukey honestly significant difference (HSD) test, two-sided, Mid–Post/Ant, *P* < 0.0001, Post–Ant, *P* = 0.420) to Ant hippocampal ROI during TI 1:3 stimulation (LMM, *F*_(2,30)_ = 359.62, *P* < 2.2 × 10^−16^; post hoc comparisons using the Tukey HSD test, two-sided, *P* < 0.0001 between all ROIs); amplitudes were normalized to total hippocampal exposure. For full statistics, see Supplementary Table 2. **d**, Participant’s performance across stimulation conditions, sham (gray), TI 1:1 (blue) and TI 1:3 (orange). Left: percentage mean response selection for each response category, showing a higher proportion of target selection compared to foils or distractors (probability of correct selection by chance was 0.2). Middle: median reaction time (RT) during recall, showing faster reaction times for target selection. Right: mean confidence rating for each response category, showing higher confidence ratings for correct associations compared to foils and distractors. There was no effect of stimulation for response type or reaction time. There was an effect of TI 1:3 stimulation for confidence rating. There was no interaction between stimulation and response category for any of the behavioral metrics. For full statistics, see Supplementary Table 4. **e**, Whole-brain group *z*-score change in BOLD signal during encode and recall. **f**, Group median change in BOLD signal in the left (L) and right (R) hippocampi in sham condition blocks. Showing significant BOLD signal increase during the encode (one-sample *t*-test, two-sided, left hippocampus *t*_(19)_ = 3.70, *P* = 0.003; right hippocampus *t*_(19)_ = 3.92, *P* = 0.003; FDR corrected), but not recall (left hippocampus *t*_(19)_ = −1.28, *P* = 0.287; right hippocampus *t*_(19)_ = −0.25, *P* = 0.805; FDR corrected); and significant effect of task stage (LMM, *F*_(1,57)_ = 20.492, *P* = 3.09 × 10^−5^; for full statistics, see Supplementary Table 5. **g**, Group median change in BOLD signal in the anterior (Ant), middle (Mid) and posterior (Post) regions of the left hippocampus during the encoding stage in the sham condition. Showing a larger BOLD signal increase in the Ant hippocampal region in relation to the Mid and Post regions (LMM, *F*_(2,38)_ = 8.72, *P* = 7.658 × 10^−4^; post hoc comparisons using the Tukey HSD test, Ant–Mid, *P* = 0.0008; Ant–Post, *P* = 0.0137; Mid–Post, *P* = 0.5518); for full statistics, see Supplementary Table 6. Asterisks identify significant differences, *P* < 0.05. Bar plots show mean and SE, black dots show individual participant data. Images in **e** were thresholded at *Z* > 3.1, with a cluster significance level of *P* < 0.05, and are displayed in *x* = −21 plane of the MNI template. *n* = 20 throughout except for **c** where *n* = 16.

After establishing that the TI stimulation locus could be focally and steerably targeted to the hippocampus, we aimed to test whether the stimulating fields could modulate hippocampal neural activity. We applied TI stimulation to 20 healthy participants (mean age ± standard deviation (s.d.) 27.1 ± 7.6 years, 11 females) while measuring brain activity using blood-oxygenation-level-dependent (BOLD) fMRI. Endogenous background activity is known to modulate stimulation-induced changes in BOLD signal^23,24^. Therefore, to evoke hippocampal activity during stimulation, participants performed a hippocampal-dependent face–name paired associative task (Fig. 2a), known to robustly evoke BOLD signal in the hippocampus^25,26^.

Stimulation was applied using the same electrode configuration as before (Fig. 1b) across three conditions: (1) TI 1:1 (2.005 kHz, 2 mA, 0.9 mA cm^−^^2^; and 2 kHz, 2 mA, 0.9 mA cm^−^^2^), and (2) TI 1:3 (2.005 kHz, 1 mA, 0.45 mA cm^−^^2^; and 2 kHz, 3 mA, 1.35 mA cm^−^^2^), but with two-fold larger amplitudes, and (3) a sham condition (2.005 kHz, 0 mA, 0 mA cm^−^^2^; and 2 kHz, 0 mA, 0 mA cm^−^^2^). We chose a Δ*f* of 5 Hz within the theta-band due to the evidential bases for the role of hippocampal theta-band oscillation in episodic memory^27^. Stimulation was only applied for a short period during the encoding stage of each block (that is, 32 s). Each participant received three blocks of each stimulation condition (that is, a total of 96 s per stimulation condition) in the same session in a counterbalanced order between participants (Fig. 2a). We chose this on/off design to minimize stimulation build-up while maximizing signal-to-noise ratio to assess physiological responses.

We first assessed whether tuning the current ratio from 1:1 to 1:3 steered the TI stimulation locus toward the Ant hippocampal region (Fig. 2b). We performed individualized simulations based on participants’ anatomical models and electrode locations (four subjects were excluded from the modeling because their electrodes were not visible in the MRI). We found that TI with 1:1 current ratio resulted in a relatively larger envelope modulation amplitude in the Mid region of the left hippocampus (Ant: 0.32 ± 0.03 median ± s.d. relative to total hippocampal exposure; Mid: 0.37 ± 0.02; Post: 0.31 ± 0.03; linear mixed model (LMM): *F*_(2,30)_ = 26.05, *P* = 2.7 × 10^−7^; Mid–Post/Ant, *P* < 0.0001, Post–Ant, *P* = 0.42; Fig. 2c). Changing the current ratio to 1:3 indeed steered the location with the largest amplitude to the Ant region of the left hippocampus (Ant: 0.40 ± 0.02; Mid: 0.34 ± 0.01; Post: 0.26 ± 0.02; LMM: *F*_(2,30)_ = 359.62, *P* < 2.2 × 10^−16^; *P* < 0.0001 across all regions, Fig. 2c and Supplementary Table 2), as predicted by simulations in the MIDA model.

We then assessed participants’ behavioral performance in the face–name task. Recall accuracies were above chance for all stimulation conditions (*t* > 10, *P* < 0.001, Supplementary Table 3), with no difference in accuracy or recall time between stimulation conditions (Fig. 2d and Supplementary Table 4). There was a main effect of stimulation in the confidence rating, explained by a small but general increase in confidence during the TI 1:3 condition (*χ*^2^(2) = 10.43, *P* = 0.005, estimated mean ± standard error (SE), sham: 2.07 ± 0.08, TI 1:1: 2.08 ± 0.08, TI 1:3: 2.20 ± 0.08; Fig. 2d), but no interaction between stimulation condition and response type (*P* = 0.25). Stable performance across conditions is advantageous for the physiological investigation of target engagement since changes in behavioral performance can confound the specificity of stimulation-induced modulations of the BOLD signal. This is because nonspecific fluctuations in brain state (such as attention^28^) or indirect stimulation of cortical sites^29^ can also induce changes in behavior and hippocampal BOLD signal.

In the absence of stimulation (that is, sham condition), the task elicited evoked BOLD activity in both hippocampi during encoding (one-sample *t*-test, left hippocampus *t*_(19)_ = 3.70, *P* = 0.003; right hippocampus *t*_(19)_ = 3.92, *P* = 0.003; false discovery rate (FDR) corrected), but not during recall (left hippocampus *t*_(19)_ = −1.28, *P* = 0.29; right hippocampus *t*_(19)_ = −0.25, *P* = 0.81; FDR corrected; Fig. 2f), similar to previous reports^26,30^. The LMM confirmed the significant effect of task stage (*F*_(1,57)_ = 20.492, *P* = 3.09 × 10^−5^) and lack of hemisphere or interaction between the two (*F* < 0.6, *P* > 0.4, Supplementary Table 5). Along the hippocampal longitudinal axis (Fig. 2b), the BOLD signal increase in the left hippocampus during encoding was largest in the Ant region (main effect of ROI, *F*_(2,38)_ = 8.72, *P* < 0.001; Ant–Mid, *P* < 0.001; Ant–Post, *P* = 0.014; Mid–Post, *P* = 0.55; Fig. 2g and Supplementary Table 6). Across the left hippocampal regions, the BOLD signal was larger when the memory association was encoded correctly (main effect of response type (correct versus incorrect), *F*_(1,95)_ = 11.09, *P* = 0.001 and ROI, *F*_(2,95)_ = 4.58, *P* = 0.012, but no interaction, *F*_(2,95)_ = 0.94, *P* = 0.39, Supplementary Table 7). These results show left hippocampal activity is modulated during formation of correct associations, consistent with previous studies^25^. In contrast, the BOLD signal increase in the right hippocampus during encoding was similar across hippocampal regions (*F*_(2,38)_ = 0.20, *P* = 0.82), and we did not observe a difference in BOLD signal between correct and incorrect encodings (*F* < 1.4, *P* > 0.3, Supplementary Table 7).

### Targeted modulation of memory-evoked hippocampal activity

**Fig. 3:**
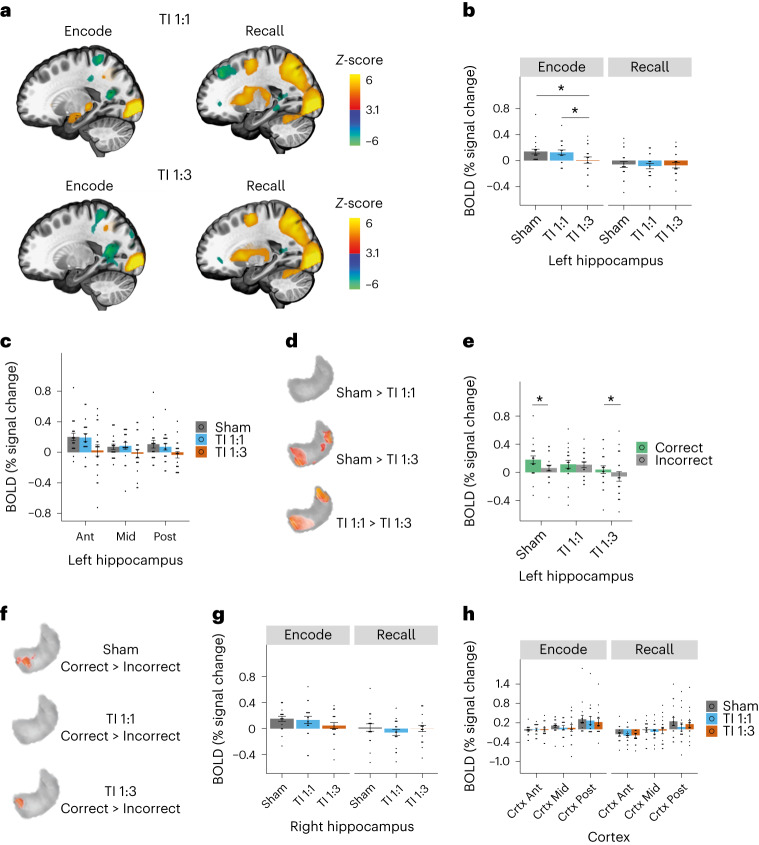
Effect of TI stimulation on hippocampal episodic memory activity. **a**, Whole-brain group *z*-score change in BOLD signal during encode and recall stages of the task for TI 1:1 and TI 1:3 stimulation conditions. Note the increase in BOLD signal in the left hippocampus during encode for the TI 1:1 condition, similar to the pattern observed for sham (Fig. 2e), but not for the TI 1:3 condition. **b**, Comparison of group median change in BOLD signal between stimulation conditions, in the left (stimulated) hippocampus during encoding and recall stages. Showing an effect of stimulation and reduction in the evoked BOLD signal in the left hippocampus during encoding stage by TI 1:3 stimulation (LMM, effect of stimulation *F*_(2,95)_ = 3.2, *P* = 0.0443; task stage *F*_(1,95)_ = 44.84, *P* = 1.49 × 10^−9^; interaction *F*_(2,95)_ = 2.96, *P* = 0.056; post hoc comparisons using the Tukey honestly significant difference test, two-sided, Encode: Sham–TI 1:1, *P* = 0.953; Sham–TI 1:3, *P* = 0.006; TI 1:1–TI 1:3, *P* = 0.015); for full statistics, see Supplementary Table 8. **c**, Comparison of group median change in BOLD signal between stimulation conditions, in the Ant, Mid and Post regions of the left hippocampus during the encoding stage; for ROIs schematic, see Fig. 2b. There is a significant main effect of stimulation in the evoked BOLD signal during the TI 1:3 stimulation; for full statistics, see Supplementary Table 9. **d**, Voxelwise group-level contrasts comparing stimulation conditions in the left hippocampus, confirming higher BOLD signal for the sham and TI 1:1 conditions compared to TI 1:3. No significant voxels for the comparison between sham and TI 1:1, confirming the results observed in the hippocampal ROIs. **e**, Group median change in BOLD signal in the left hippocampus for memory associations encoded correctly (green) and incorrectly (gray). Showing significantly higher BOLD signal for correct compared to incorrect associations in the left hippocampus during sham (LMM, effect of response type (correct, incorrect) *F*_(1,95)_ = 11.09, *P* = 0.001), and TI 1:3, (LMM, effect of response type, *F*_(1,95)_ = 6.6, *P* = 0.0117); for full statistics, see Supplementary Fig. 4 and Supplementary Table 10. **f**, Voxelwise group-level contrasts comparing correctly and incorrectly encoded associations in the left hippocampus, showing that BOLD signal during the formation of correct associations is predominantly modulated in a cluster located in the anterior portion of the hippocampus for sham and TI 1:3 conditions, while no significant voxels were observed for the TI 1:1 condition. **g**, Same as **b** but for the right hippocampus, where there is no effect of stimulation; for full statistics, see Supplementary Table 8. **h**, Comparison of group median percentage change in BOLD signal between stimulation conditions, in the anterior (Ant), Middle (Mid) and posterior (Post) regions of the overlying cortex; for ROIs schematic, see Fig. 1d; for full statistics, see Supplementary Table 12. No difference in the BOLD signal change between stimulation conditions. Similar results were obtained for the brain regions underneath the stimulation electrodes in the right hemisphere; for full statistics, see Supplementary Fig. 5 and Supplementary Table 13. Asterisks identify significant differences, *P* < 0.05. Bar plots show mean and SE, and black dots show individual participant data. Images in **a** were thresholded at *Z* > 3.1, with a cluster significance level of *P* < 0.05, and are displayed in *x* = −21 plane of the MNI template. Images in **d** and **f** were thresholded at significance level of *P* < 0.05, TFCE FWE corrected, voxelwise permutation-based *t*-tests on the ROI. *n* = 20 throughout except for **h** where *n* = 16.

We next assessed whether TI stimulation affected the BOLD signal evoked by the face–name task in the left, that is, targeted, hippocampus. We found that TI 1:1 stimulation did not significantly change the BOLD signal in the hippocampus (Fig. 3a top and Fig. 3b). In contrast, TI 1:3 stimulation that was steered to the Ant region, reduced the BOLD signal (effect of stimulation *F*_(2,95)_ = 3.2, *P* = 0.04; task stage *F*_(1,95)_ = 44.84, *P* = 1.49 × 10^−9^; interaction *F*_(2,95)_ = 2.96, *P* = 0.056; Encode: Sham–TI 1:1, *P* = 0.953; Sham–TI 1:3, *P* = 0.006; TI 1:1–TI 1:3, *P* = 0.015; Fig. 3a bottom, Fig. 3b and Supplementary Table 8). The reduction in the evoked BOLD signal by the TI 1:3 stimulation in the left hippocampus was significant across hippocampal regions, with larger nominal magnitude in the Ant region exposed to the largest relative envelope modulation amplitude, (Fig. 3c, effect of stimulation *F*_(2,152)_ = 12.65, *P* = 8.13 × 10^−6^ and ROI, F_(2,152)_ = 6.35, *P* = 0.002; but no interaction between the two *P* = 0.76; mean difference: Ant_(Sham)_ − Ant_(TI 1:3)_ = 0.18, Mid_(Sham)_ − Mid_(TI 1:3)_ = 0.09, Post_(Sham)_ − Post_(TI 1:3)_ = 0.14, Supplementary Table 9). This pattern was confirmed in the voxelwise analysis comparing stimulation conditions in the left hippocampus (Fig. 3d, *P* < 0.05, threshold-free cluster enhancement (TFCE) family-wise error (FWE) corrected). There were no voxels with significant BOLD differences between sham and TI 1:1 stimulation, whereas during TI 1:3 stimulation there was a significant reduction in BOLD activity predominantly in the Ant and Post segments of the hippocampus in relation to both sham and TI 1:1 stimulation (percentage voxels with significant signal change, Sham > TI 1:3: Ant 30%, Mid 2%, Post 25%; TI 1:1 > TI 1:3: Ant 31%, Mid 3%, Post 25%). The amplitude of the evoked BOLD signal in the left hippocampus during TI 1:3 stimulation, but not TI 1:1 stimulation, was larger when the memory associations were encoded correctly as in the sham condition (Fig. 3e, Supplementary Fig. 4 and Supplementary Table 10). In addition, the spatial pattern of activity in correct compared to incorrect trials was similar between sham and TI 1:3 conditions (Fig. 3f). This indicates that, despite a reduction in BOLD signal during the TI 1:3 stimulation, the relative signal difference between correct and incorrect encodings is maintained. The magnitude of the BOLD signal in the left hippocampal regions for the different stimulation conditions was not correlated with recall accuracy (*P* > 0.2, Supplementary Table 11).

Next, we assessed whether the BOLD signal was modulated in the right (not targeted) hippocampus and the overlaying cortical regions underneath and between the stimulation electrodes. There were no significant differences in BOLD signal in the right hippocampus (Fig. 3g, *F* < 2, *P* > 0.1, and Supplementary Table 8) or ROIs close to the stimulation electrodes (Fig. 3h, *P* > 0.4, and Supplementary Table 12; Supplementary Fig. 5 for right hemisphere). To support that the lack of changes in BOLD signal was not driven by anatomical variability (electrode locations determined using landmarks based on head size) or reduced sample (16 out of 20), we proceeded to extract BOLD signal in the left temporal lobe (excluding the hippocampus) for all participants. There was no significant effect of stimulation on BOLD signal in the left temporal ROI (*P* > 0.2, Supplementary Table 12), which was again confirmed by group-level voxelwise analysis. As a final interrogation on the spatial specificity of the BOLD changes, we investigated whether BOLD signal was modulated in the left amygdala, located anteriorly to the stimulated hippocampus. We did not observe changes in BOLD signal for the left amygdala (Supplementary Fig. 6). The lack of BOLD signal change in the overlaying cortical regions and neighboring amygdala cannot be explained by a lack of task activation since, across conditions, we observed evoked BOLD signal in these regions (Supplementary Table 13).

Taken together, our results demonstrate a non-invasive focal modulation of evoked neural activity in the targeted hippocampus. The hippocampal decrease in BOLD signal observed during the TI 1:3 condition is in alignment with previous animal studies, showing theta frequency stimulation decreases the magnitude of the BOLD signal in the hippocampus^31^. This would suggest that larger field magnitudes should result in larger decreases in BOLD signal. We observed some evidence to support this relationship (significant Pearson, but no significant robust correlations possibly due to the small sample size) in the same hippocampal regions where BOLD signal was mostly modulated by the stimulation (‘Ant’ and ‘Post’ regions, Supplementary Fig. 7).

### Modulation of hippocampal functional connectivity

**Fig. 4:**
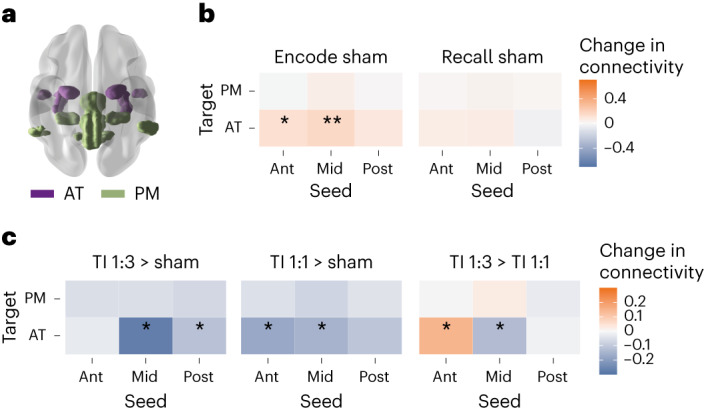
TI stimulation change in hippocampal–cortical FC. **a**, AT (purple) and PM (green) hippocampal–cortical networks. **b**, Group mean change (% signal change) in FC between the anterior (Ant), Middle (Mid) and posterior (Post) regions of the left (L) hippocampus and the AT and PT networks during encode and recall task stages in sham blocks (that is, without stimulation) for the contrast correct > incorrect. Showing a larger connectivity between the Ant and Mid regions of the L hippocampus and the AT network during encoding of successful associations; (one-sample *t*-tests, two-sided; Ant → AT, *P* = 0.0223, *P*(FDR) = 0.1336; Mid → AT, *P* = 0.0024, *P*(FDR) = 0.0287); **P < 0.05 FDR corrected; *P < 0.05 uncorrected; for full statistics, see Supplementary Table 14. **c**, Effect of TI stimulation on FC, showing the same as **b** but comparing changes between stimulation conditions during encoding stage in which a connectivity increase was observed in sham blocks. A reduction in FC by both TI 1:1 and TI 1:3 stimulations (relative to sham) and, when comparing the TI stimulations, a larger connectivity in the Mid region during TI 1:1 and in the Ant region during TI 1:3 (LMM with significant three-way interaction between stimulation type, seed and network, *F*_(4,1576)_ = 2.54, *P* = 0.038; post hoc comparisons using the Tukey honestly significant difference test, two-sided; Ant → AT: TI 1:1–Sham, *P* = 0.0010, TI 1:3–TI 1:1, *P* = 0.0082; Mid → AT: TI 1:1–Sham, *P* = 0.0059, TI 1:3–Sham, *P* < 0.0001, TI 1:3–TI 1:1, *P* = 0.0232); *P < 0.05; for full statistics, see Supplementary Table 14). *n* = 20 throughout. ‘→’ indicates the direction of the connectivity from seed to target.

Given that successful associative memory involves interactions between the hippocampus and cortical networks, in particular the antero-temporal (AT; more connected to the Ant hippocampus) and posterior-medial (PM; more connected to the Post hippocampus) networks^32,33^ (Fig. 4a), we sought to explore whether stimulation of the hippocampus changes the functional connectivity (FC) in those networks. In the absence of stimulation, successful encodings increased FC between the Ant and Mid, but not Post, hippocampal regions and the AT network, (Fig. 4b, AT: Ant: *t* = 2.322, *P* = 0.022, uncorrected; Mid: *t* = 3.117, *P* = 0.029, FDR corrected, Supplementary Table 14). There was no change in FC between hippocampal regions and the PM network or during recall.

Compared to sham, both TI stimulations reduced FC between the hippocampus and the AT network. This reduction was localized to the Ant and Mid regions of the hippocampus during TI 1:1 stimulation and the Mid and Post regions during TI 1:3 stimulation (Fig. 4c, significant interaction between stimulation type, seed and network *F*_(4,1576)_ = 2.5, *P* = 0.04; post hoc tests, TI 1:1: Ant: *P* = 0.001; Mid: *P* = 0.006; Post: *P* = 0.07; TI 1:3: Ant: *P* = 0.8; Mid: *P* < 0.001; Post: *P* = 0.04). Comparison of FC between TI conditions showed a higher relative connectivity at the hippocampal region that was exposed to the largest envelope modulation amplitude. Specifically, FC between the Mid hippocampus and the AT network was larger during TI 1:1 stimulation than during TI 1:3 stimulation (*P* = 0.023; Fig. 4c). In contrast, FC between the Ant hippocampus and the AT network was larger during TI 1:3 stimulation (*P* = 0.008; Fig. 4c and Supplementary Table 14). We did not find specific seed-network FC differences between the stimulation conditions during the recall period; however, we found a main effect of stimulation, explained by lower connectivity values during the TI 1:1 stimulation (*F*_(2,1576)_ = 8.320, *P* = 2.544 × 10^−4^, Supplementary Table 15). These results suggest that the reduction in the memory evoked BOLD signal in the hippocampus occurred alongside a reduction in the FC between the hippocampus and its AT cortical network.

### Enhancing hippocampal-dependent episodic memory performance

We next aimed to explore whether TI stimulation of the hippocampus could affect the underlying memory function. We tested a new cohort of twenty-one participants (mean age ± s.d. 22.7 ± 3.2 years, 10 females) with a similar hippocampal-dependent face–name task but this time with an extended period of stimulation and a larger number of behavioral trials (Fig. 2a)—an experimental protocol designed to probe behavioral effects of stimulation^34^. We applied TI 1:3 stimulation that showed stronger modulation of hippocampal memory BOLD signal and sham in counterbalanced order in two separate experimental sessions. In contrast to the first experiment, we applied the stimulation continuously not just during encoding but also during the maintenance and recall periods. Furthermore, since earlier studies have shown that retrieving a memory can transform the information being remembered^35,36^, thereby facilitating or impeding the memory^37^, we explored this effect by retesting all the face–name pairs again after 30 min.

**Fig. 5:**
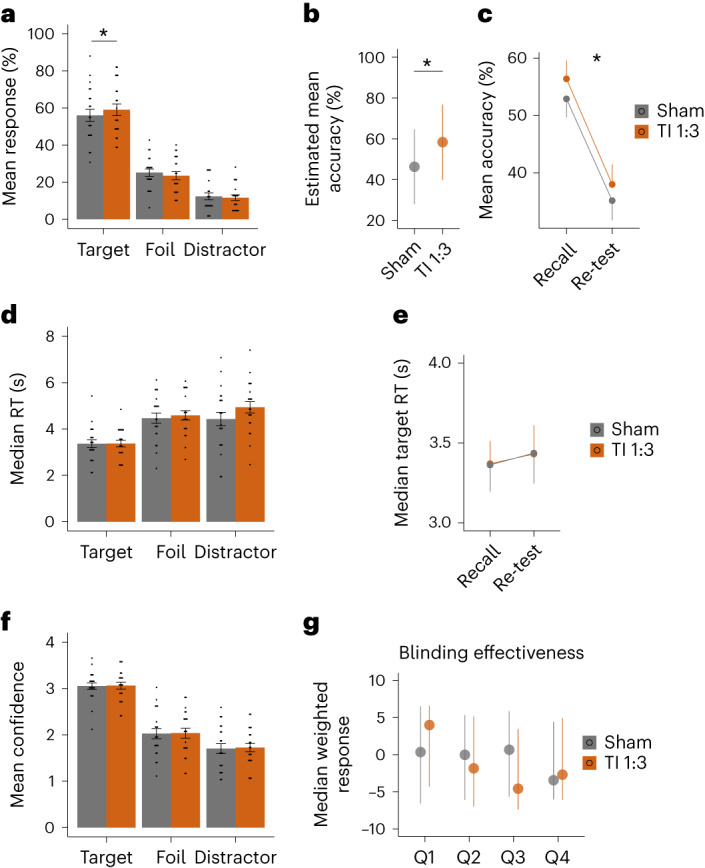
Probing the effect of hippocampal TI stimulation on behavioral function. **a**, Comparison of participants’ memory performances during recall between sham (gray) and TI 1:3 (orange) across response type (target, foil and distractor), showing higher probability of target responses, that is, face–name pairs correctly remembered during TI (GLMM, *χ*^2^(2) = 6.353, *P* = 0.0417; post hoc paired *t*-tests, two-sided, Sham–TI 1:3; Target, *P* = 0.0068; Foils, *P* = 0.1418, *P* = 0.3836). **b**, Estimated mean accuracy for sham and TI 1:3, as estimated using a mixed-effects logistic regression model for correct and incorrect responses and independent random-effect terms for ‘participant’, ‘session’ and ‘task block’, showing higher odds of correct recall during TI stimulation (logit scale; GLMM, *χ*^2^(2) = 5.857, *P* = 0.0155). **c**, Comparison of mean accuracy for target selections between recall and re-test (30 min after first recall, for items correctly remembered at recall) for sham and TI 1:3, showing that target selection was higher for TI 1:3 condition at both time points (GLMM, main effect of stimulation, *χ*^2^(1) = 7.581, *P* = 0.006). **d**, Same as **a** but for median reaction time (RT), showing no differences between stimulation conditions. **e**, Same as **b** but for median RT for target responses, showing no significant difference between recall and re-test or stimulation conditions. **f**, Same as **c** but for mean confidence ratings, which were similar between stimulation conditions. **g**, Blinding effectiveness. Shown are median weighted scores and 95% confidence intervals for TI 1:3 and sham conditions. Participants were asked at four time points during each session (indicated by a Q on the *x* axis) whether they thought they had stimulation and how confident they were (1 is not confident at all; 10 is extremely confident). The two questions were combined into a weighted score, whereby a ‘yes’ answer was assigned a +1 value and ‘no’ answer a value of −1, which were then multiplied by the confidence rating. Thus, a positive score on the *y* axis indicates that participants reported having stimulation, and negative score not having stimulation. No significant effects of stimulation; for full statistics, see Supplementary Table 24. In **a**–**f**, bar or dot plots show mean and SE, and black dots show individual participant data. Asterisks indicate significant differences between stimulation conditions. For full statistics, see Supplementary Table 16. *n* = 21 throughout.

We found an effect of TI stimulation on participants’ performance (generalized linear mixed model (GLMM): *χ*^2^(2) = 6.353, *P* = 0.042; Fig. 5a). Specifically, participants showed higher proportions of correct (that is, target) recalls during TI compared to sham (*P* = 0.007), with no difference in the number of foils (*P* = 0.142) or distractors (*P* = 0.384). Given the lack of difference in foils and distractors (that is, incorrect responses), we followed the analysis with a frequentist binomial model (that is correct and incorrect responses as the dependent variable). This confirmed the higher odds of correct recall during TI stimulation (*χ*^2^(2) = 5.857, *P* = 0.016; Fig. 5b), as did the equivalent Bayesian model, showing stimulation increased the odds of selecting the target by 12% (mean posterior estimate 0.12, 95% credible interval (CI) 0.02 to 0.21; 99.15% of the posterior >0, Supplementary Table 16). The total stimulation duration differed slightly between participants (mean ± s.d. 34.5 ± 3 min) due to self-paced responses. However, recall accuracy was not correlated with stimulation duration (*r* = −0.045, *P* = 0.85; Pearson correlation). While accuracy for all re-test items did not differ between TI and sham (*P* > 0.2, Supplementary Table 17), focusing the analysis to items that were correctly remembered at recall showed an effect of stimulation (*χ*^2^(1) = 7.581, *P* = 0.006) and an effect of time of testing (*χ*^2^(1) = 233.124, *P* < 0.001), but no interaction (*χ*^2^(1) = 0.063, *P* = 0.802), suggesting that the memory benefit gained during TI stimulation was maintained at re-test (Fig. 5c; for additional analysis of re-test response patterns, see Supplementary Fig. 8).

The improvement in recall accuracy was not accompanied with a change in recall time, (Fig. 5d, GLMM/LMM: no main effect of stimulation using multinomial logistic regression *χ*^2^(1) = 3.017, *P* = 0.082, or binomial model *χ*^2^(1) = 2.993, *P* = 0.084; no interaction between stimulation and recall time, *P* > 0.2). Comparison between median reaction times for recall and re-test for correct recalled items showed no effect of stimulation, time or interaction (Fig. 5e, *χ*^2^ < 0.6, *P* > 0.4). Similarly, there was no effect of stimulation on confidence rating during recall (Fig. 5f, no main effect of stimulation *χ*^2^(1) = 0.35, *P* = 0.557, or interaction between confidence per response category and stimulation *P* = 0.809, Supplementary Table 16).

### Safety, tolerability and blindness

Across both experiments, participants tolerated well the TI stimulation. There were no adverse effects and only a few incidences of mild common side effects (Supplementary Tables 19 and 20). In the last experiment, where stimulation and sham were performed in separate sessions, we could directly compare the incidence of side effects. We observed that only itchiness at the electrode site was higher for TI than sham stimulation (*Z* = −2.354, *P* = 0.019, Supplementary Table 20). Despite the high current densities the threshold current intensity at which participants reported perceiving extraneous skin sensation underneath the electrodes was much higher than when we tested short conventional transcranial alternating current stimulation (tACS) during setup (mean ± s.d., tACS: 0.424 ± 0.195 mA; TI: 2 ± 0.540 mA; *t*_(65)_ = 17.203, *P* < 0.001, unpaired two-tailed *t*-test, *n* = 42 reporting perceptual sensations during tACS and *n* = 25 during TI, pooled from both studies; Supplementary Tables 21–23).

Finally, we assessed stimulation blindness at four time points during each session of the behavioral experiment. We found no difference between TI stimulation and sham in participants’ weighted confidence of receiving stimulation, indicating appropriate blinding (Fig. 5g, *P* > 0.2 and Supplementary Table 24).

## Discussion

Here we present the first demonstration of non-invasive electrical DBS in humans using TI of kHz electric fields, expanding on our earlier validation in rodents^14,38^. We first used electric field modeling and measurements in a human cadaver to verify that the locus of transcranial TI stimulation can be steerably localized to the human hippocampus with minimal exposure of the overlying cortex. We then use neuroimaging and behavioral experiments in healthy humans to demonstrate focal non-invasive modulation of hippocampal memory activity and the capacity to augment memory performance.

Our modeling predicts that the TI fields locus in the hippocampus have a median amplitude of ~0.25 V m^−1^ per ~0.45 mA cm^−^^2^ applied current density (1 mA current in our electrodes), consistent with previous computational studies^39,40^. This yields a median hippocampal envelope amplitude of ~0.5 V m^−1^ in our human studies (~0.9 mA cm^−^^2^ applied current density) and ~0.2 V m^−1^ difference between the Ant and the Mid/Post regions. Similar field amplitudes have been consistently reported to synchronize neural spiking activity tuned to the endogenous oscillation frequency range in vitro^41^ and in vivo^42,43^. One limitation of our cadaver measurements is that data were collected at a temperature lower than the living body, resulting in lower tissue conductivity and, consequently, higher electric field amplitudes for fixed current densities^44^. However, since the electric field amplitudes change equally across the head tissues^44^, the relative field distribution estimations in the cadaver were not affected.

Our neuroimaging experiments aimed to probe a focal physiological effect. We demonstrate that when the TI stimulation locus is transiently applied to the hippocampus with a theta-band difference frequency during encoding of episodic memory, it reduces the hippocampal evoked BOLD signal without affecting evoked BOLD signal in the overlying cortex. The BOLD reduction was strongest when the TI stimulation locus was steered to the anterior hippocampus, in line with the repeated reports on this region’s central role in the successful encoding of face–name associations^21,25,26^. Our results agree with the literature in similar cohorts, reporting differences in hippocampal activity between remembered and subsequently forgotten items without the magnitude of the BOLD signal correlating with the proportion of correct responses^21,45^. Furthermore, focal physiological changes in hippocampal activity could be measured without confounds from behavioral differences. This finding supports robust inference of target engagement.

Our subsequent experiment aimed to probe the behavior consequence of the physiological effect in the hippocampus. We designed the behavioral experiment to include ~4 times more associations per condition and ~20 times longer stimulation durations, delivered during the encoding, maintenance and recall stages, in agreement with the parameters typically used in other non-invasive tES experiments^34^. We demonstrate that in these conditions TI provides an improvement in memory accuracy. The magnitude of the memory improvement was small, but in line with many tES studies modulating working memory performance^34^. We observed that memories formed during TI endured the effects of re-test, but the rate at which they were forgotten was similar to sham. Future studies aimed at understanding longer-term effects of stimulation in memory should investigate whether more extended continuous TI stimulation and/or repeated sessions may be able to achieve stronger and sustained memory benefits. Those studies may be able to pinpoint the optimal stimulation timing (that is, memory encoding, maintenance and/or recall) and include fMRI designs that allow for more nuanced analyses of the activity patterns between each stage of the task, using for example multivariate approaches^46^.

What is the possible mechanism by which theta-band TI stimulation of the hippocampus improves episodic memory function? A substantial body of evidence shows that formation of episodic memories involves hippocampal theta oscillations^27^, which coordinate periodic changes in excitability that synchronize spiking activity across the hippocampal network (without affecting mean spiking rate)^47,48^. Synchronization of spiking activity critically amplifies the transfer of information above the background activity and promotes sustained access via synaptic spike time-dependent plasticity^27,49^. Since TI stimulation modulates neural activity at the difference frequency of the kHz-frequency electric fields^14^, we hypothesize that its application with a theta-band difference frequency will augment the endogenous theta synchronization in the hippocampus thereby improving the underlying memory function.

What is the possible mechanism by which theta-band TI stimulation of the hippocampus reduces the magnitude of the BOLD signal and FC evoked by episodic memory? The BOLD signal reflects a complex set of neurovascular processes whereby local changes in neuronal activity drive changes in blood oxygenation and blood flow^50^. One possible explanation for our findings is that by augmenting endogenous theta synchronization in the hippocampus, the stimulation decreased underlying metabolic demand resulting in reduced BOLD signal. The smaller BOLD signal in the hippocampus may weaken its coherent fluctuations, thereby reducing the correlation with the rest of the AT network. Indeed, earlier human studies with intracranial electroencephalography recordings from the hippocampus have shown higher coherence but lower power spectrum at the theta band during encoding of episodic memories that were subsequently remembered versus not remembered^51–54^. Alternatively, the reduced BOLD signal might index an increase in metabolic activity that is not met by a parallel increase in oxygenated hemoglobin^55^. The difficulty in linking changes in BOLD signal with changes in neural oscillations is well documented^56^, particularly in the hippocampus and medial temporal lobe, where negative correlations between BOLD signal and neuronal activity and theta-gamma phase amplitude coupling have been shown^57^. Furthermore, the low signal-to-noise ratio of the BOLD and the dynamic change in the strength of the neurovascular coupling itself renders linking matrices of BOLD correlation and neural activity challenging^50^. Yet, our observed reduction of hippocampal BOLD signal due to the TI stimulation is consistent with previous reports in animal models delivering theta frequency electrical stimulation to the hippocampal circuit^31^.

Although our current data do not allow us to disambiguate which components of the BOLD signal are affected by TI stimulation, it is unlikely that the effect observed can be simply explained by vascular changes. This is because no changes in BOLD signal were observed when TI was directed to the Mid hippocampus. Further, changes in FC were specific to the AT cortical network, and their modulation between the two TI conditions was in line with TI fields’ distribution along the hippocampal longitudinal axis, providing further evidence of the TI stimulation specificity and steerability. Studies with concurrent fMRI and intracranial electroencephalography recordings from the hippocampus will be able to elucidate the direct neural response and offer further mechanistic insights to the observed BOLD changes.

Could the change in hippocampal BOLD signal and memory performance have been mediated by stimulation of the overlying cortex exposed to larger field amplitudes? The lack of stimulation effect on the evoked BOLD signal in the overlying cortex renders this possibility unlikely. Could the observed change in hippocampal BOLD signal and memory performance have been mediated by conventional AC stimulation of the kHz fields in the hippocampus? A recent electrophysiological study investigating the effect of kHz-frequency electric fields in hippocampal brain slices reported no effect on neural activity, even with amplitudes that are two orders of magnitude larger than those used in this study^58^, consistent with our earlier electrophysiological study in the live mouse brain^14^. In our experiments, the absolute amplitude of the applied electric fields (that is, the strength of conventional AC stimulation) was larger at the cortical region near the electrodes, while the envelope modulation amplitude (that is, the strength of TI stimulation) was larger at the underlying hippocampal region. This provides a robust general way of testing the TI hypothesis with ‘intrinsic’ stimulation controls. Our results render the possibility of conventional AC stimulation by the kHz fields low, for three main reasons: (1) lack of stimulation effect on the evoked BOLD signal in the overlying cortex where the absolute amplitude was largest; (2) effect of TI 1:3 condition that steered the envelope amplitude within the hippocampus while keeping the total cortical AC field exposure fixed; and (3) evidence of a correlation, albeit weak, between the participants’ change in hippocampal BOLD signal and the envelope modulation amplitude in the hippocampus, but not between BOLD and the absolute amplitude in the overlying cortex or hippocampus. Nevertheless, a general limitation of our fMRI and behavioral studies is the modest sample sizes that, while sufficient to demonstrate physiological and behavioral effects, impose limitations to the establishment of brain–behavior relationships. An additional limitation of our study is an inconsistency in the intra-hippocampus spatial selectivity across investigations. Although the TI 1:3 with a higher field exposure in the Ant hippocampal region (established from cadaver measurements and MRI-based individualized simulations) reduced the hippocampal BOLD signal relative to sham and TI 1:1 stimulation, the change within the hippocampus was not entirely localized to the anterior region. The effects on FC were more complex (that is, TI 1:3 versus TI 1:1 was consistent with the relative TI field distribution but not TI 1:3 versus sham). The variability in the BOLD response distribution to our non-invasive DBS via TI is, however, consistent with studies using invasive DBS reporting that the BOLD response distribution can depend on contextual factors such as the patient characteristics and therapeutic outcomes (for a recent review, see Loh et al.^59^). Indeed, the complexity of the neurovascular coupling dynamics renders a direct linear relationship between neural activity, local BOLD signal, and network BOLD signal coherence unlikely^56^. Future studies using TI stimulation could uncover this complex contextual relationship in the hippocampus by repeating the experiment under different physiological and pathophysiological states.

Overall, TI stimulation was well tolerated, no adverse effects were recorded, and side effects were mild. We used current densities that are considered safe and in line with those applied across tES studies, where no serious adverse effects have been reported^60^. We found that the thresholds at which TI stimulation produces extraneous sensations are much higher than those for conventional tACS and allowed for adequate stimulation blinding. This is useful as therapeutical applications might benefit from higher current densities for which blinding becomes harder to achieve^61^.

The hippocampus is important in a myriad of brain functions, including learning and memory, spatial navigation and emotional behavior. It also plays a central role in many of the most common brain disorders^62^. The evidence for reduced BOLD signal during TI stimulation to the anterior hippocampus offers a possible means of targeting hippocampal hyperactivity, which is present in prodromal Alzheimer’s disease (where it is associated with subsequent cognitive decline)^63,64^, schizophrenia^65^ and temporal lobe epilepsy^66^. By modulating hippocampal neural activity non-invasively, TI stimulation offers new opportunities to probe its causal role in brain functions. Future studies using different electrode configurations and current parameters may sculpt the TI locus to focally modulate neural activity in other deep brain structures. Tuning the difference frequency of the applied kHz-frequency fields will allow exploring the frequency band specificity of the target brain regions and contribute to inform stimulation optimization strategies to treat brain disorders.

## Methods

### Ethics oversight

Data for the reference head model used in this study were previously published and collected in accordance with the appropriate ethical approval from the Institute for Biomedical Engineering at the ETH, Zurich, Switzerland. Ethical approval for the human cadaver experiments was granted by the Faculty of Medicine La Timone (Aix Marseille Université). Ethical approval for in vivo experiments with human participants was granted by the Imperial College Research Ethics Committee (ICREC). Participants gave written informed consent and those taking part in the in vivo experiments were compensated for their time. The study conforms to the Declaration of Helsinki.

### Electric field simulations

To characterize the in vivo exposure to high-frequency fields and to low-frequency TI modulation, as well as for the identification of optimized stimulation configurations, dosimetric electromagnetic simulations involving anatomical head models were performed. Two kinds of head models were used: (1) a highly detailed and accurate reference head model, and (2) personalized head models that permit studying the relationship between inter-subject anatomy and exposure variability and subject-specific BOLD response.

### Reference head model

For maximal simulation realism, the highly detailed MIDA head model jointly developed with the US Food and Drug Administration was used (one healthy 29-year-old female volunteer)^19^. This model is based on high-resolution (<0.5 mm throughout) multi-modal MRI data, which allows for the distinction of more than 100 different tissue regions, including a range of deep brain targets, the crucial distinction of cancellous and cortical skull layers, the dura, various scalp layers (skin, muscle, fat and tunica) and the complex distribution of cerebrospinal fluid, among other tissues. Co-registered DTI data provide the necessary information about brain heterogeneity and anisotropy, as well as the local principal orientation of fibers. In the simulations with the reference MIDA model, the scalp electrode geometry was circular (2 cm diameter, 3.14 cm^2^ area), while the ex vivo and in vivo experiments used square electrodes with rounded corners (1.5 cm × 1.5 cm, 2.25 cm^2^ area). Thus, the electrode surface area was 1.4× larger in the simulations, resulting in a 30% average current density reduction for a given current amplitude when compared to the ex vivo and in vivo experiments. The simulation in the reference model was done as a first step with the aim of establishing the feasibility of localizing the locus of TI stimulation to the human hippocampus. We later reduced the surface area of the scalp electrodes to maximize the hippocampal field amplitude. In the simulations in the MIDA model, the applied electric currents were 1 mA per electrode pair, rather than the 2 mA from the in vivo experiments. Importantly, the total current magnitude does not affect the relative electric field distribution. Similarly, the relative TI exposure distribution depends only on the ratio of the applied currents and not on the absolute scaling. Measuring and simulating the electric field distributions for a unit current amplitude (for example, 1 mA as in our study) facilitates the generation of exposure maps for arbitrary current amplitudes (the electric fields are simply multiplied with the current amplitude applied in the study).

### Personalized head models

Individualized (though less accurate and detailed) head models were generated from T1 images (see ‘MRI data acquisition’ section) using the SimNIBS framework (version 3.2 (ref. ^68^)), employing the ‘headreco’ pipeline^69^ to distinguish six tissue classes: scalp, skull, cerebrospinal fluid (CSF), gray matter, white matter and head cavities. Segmented images were visually inspected and manually corrected when necessary (manual corrections were mostly restricted to the skull–CSF boundary). Because the hippocampi were not included in the automatic segmentation from SimNIBS, these were extracted using FreeSurfer (see ‘ROIs’ section) and converted into tissue surfaces using the iSEG software (IT’IS Foundation, Zurich, Switzerland). Using subject-specific images also permitted to accurately position the scalp electrodes on the reconstructed scalp surfaces in 16 out of 20 participants from the fMRI experiment (in 4 participants the field of view of the T1 image did not allow for a clear localization of the electrodes on the skin and accurate scalp and skull tissue segmentations; Supplementary Fig. 3). In the individualized simulations, the scalp electrode geometry was circular instead of a square with rounded corners, as in the in vivo experiments. The circular electrode shape was due to technical constraints. However, since the surface areas were identical and the geometric differences were minute, we do not expect differences in the transcranial cerebral field distribution. The electric field distributions were first generated for 1 mA and then extracted in the ROIs and multiplied by the current amplitude used in vivo.

### Electromagnetic field computation

The reference and the reconstructed subject-specific head models were imported as surface mesh entities into the Sim4Life (ZMT ZurichMedTech AG) platform with extended TI modeling functionality. Electrode geometries (2-cm-diameter cylinders) were created in Sim4Life, placed at the identified electrode locations, and aligned to the head surfaces, while ensuring gap-less contact. The setup for EM simulations consisted of dielectric property and boundary condition assignment, followed by gridding and voxeling for numerical discretization. The simulations were executed using Sim4Life’s finite element method low-frequency electro-quasistatic, ohmic-current-dominated (EQSCD) solver, that computes solutions to the quasistatic and ohmic-current-dominated approximation of the Maxwell equation ($\nabla \sigma \nabla \varphi =0$, with boundary conditions) on an adaptive, rectilinear grid, where *φ* is the electric potential and *σ* the electric tissue conductivity distribution. EQSCD assumes that ohmic (resistive) currents dominate over displacement currents at the frequencies of interest and that the wave-length is large compared to the computational domain^70^—conditions that were confirmed by a solver-performed analysis. The conductivities of the non-brain tissues were assigned on the basis of a recent meta-analysis of reported human head electrical conductivity values^71^. To account for the important impact of brain tissue dielectric anisotropy and heterogeneity, DTI-based electrical conductivity tensor maps were generated. The local main orientation was derived through spectral decomposition of the DTI tensors and in turn combined with the longitudinal and transversal conductivities according to ref. ^72^, to reconstruct *σ* tensors. A convergence analysis was performed to identify an optimal grid resolution that ensures sufficient accuracy (that is, negligible discretization errors) while minimizing the number of discretization elements (voxels) to reduce computational resource requirements. Simulations were executed at a homogeneous 0.5 mm resolution, which resulted in models consisting of about 80 million voxels. Each TI stimulation exposure condition required the execution of two EM simulations per subject, one for each electrode pair. Dirichlet boundary conditions were assigned to the active electrodes (thus capturing the inhomogeneous current distribution across the electrode interface), applying an arbitrary voltage difference of 1 V subject to subsequent current normalization.

### Computed TI exposure metrics

The calculated electric (*E*) fields for each electrode pair were normalized to an input current of 1 mA, by integrating the normal current density $\mathbf{{j}}(\mathbf{r})=\sigma \mathbf{E}(\mathbf{r})$ over a spherical surface surrounding one electrode and performing a convergence analysis. The spatial distribution of the projected TI envelope modulation amplitude along the local brain structure orientation $\mathbf{n}$ was computed from the fields of both electrode pairs using $\left|{\mathbf{E}}_{\mathrm{AM}}\left(\mathbf{n,}\mathbf{r}\right)\right|=\left||\left({\mathbf{E}}_{1}\left(\mathbf{r}\right)+{\mathbf{E}}_{2}\left(\mathbf{r}\right)\right)\cdot \mathbf{n}|-|\left({\mathbf{E}}_{1}\left(\mathbf{r}\right)-{\mathbf{E}}_{2}\left(\mathbf{r}\right)\right)\cdot \mathbf{n}{\rm{|}}\right|$ where ${\mathbf{E}}_{1}(\mathbf{r})$ and ${\mathbf{E}}_{2}(\mathbf{r})$ are the fields generated by the first and second electrode pair, respectively, at the location $\mathbf{r}(x,y,z)$. The local brain structure (for example, white-matter fibers, organized pyramidal neurons in the hippocampus) orientation was identified as the principal axis of the corresponding DTI tensor. The modulation magnitude and the carrier frequency field distributions can be inspected for the MIDA model using an online-explorable 3D viewer^73^.

### Electric field measurements in human cadaver

A human male cadaver (93 years old) with no known brain disorder was provided by the ‘service des corps donnés à la science’ by Aix Marseille Université. Experiments were performed in the Faculty of Medicine La Timone (Aix Marseille Université). The subject was perfused with zinc chloride, stored in freezer until the experiments and left to warm to 20 °C before the recording session.

Electric fields were recorded using three stereoelectroencephalography 15-contact electrodes, ring diameter 0.8 mm, length 2 mm, useful exploration length 51 mm (2069-EPC-15C-35, Alcis). The sEEG electrodes were implanted in the left mesial temporal lobe, perpendicular to the hippocampal longitudinal axis. The technique of implantation was based on the neurosurgeon’s experience in performing sEEG (R.C.) and was similar to the one routinely applied to human patients for the presurgical workup of drug-resistant epilepsy. Each electrode was orthogonally inserted through a short 20-mm guidance screw (Alcis, 2023-TO-C-020) after 2.5-mm diameter drilling of the bone. The internal hole of the screw was insulated with a silicon tube, and an insulating nylon cap covered its top surface. A thin electrode lead (0.8 mm diameter) was then inserted via the screw and the insulating silicon tube. The craniotomy was tightly sealed to avoid leakage of CSF outside the head and external liquid to the head. Reference and ground electrodes were placed on the shoulder of the cadaver using ECG electrodes (WhiteSensor WS, Ambu,1.5 × 1.5 cm).

The electric potential signals from the stereoelectroencephalography electrodes were amplified and sampled at 30 kS s^−1^ using the RHS Stim/Recording Controller (Intan Technologies). The stimulating currents were applied using 1.5 cm × 1.5 cm scalp electrodes (WhiteSensor WS, Ambu) as in Fig. 1b. The two currents were generated using two electrically isolated current sources (DS5, Digitimer) driven by voltage waveforms from a function generator (Keysight, EDU33212A). In the case of TI stimulation, we applied one current at 2.005 kHz frequency and 1 mA (peak to baseline, current density ~0.45 mA cm^−^^2^) amplitude and a second current at 2 kHz frequency and same amplitude (that is, TI with Δ*f* = 5 Hz and 1:1 current ratio; Fig. 1c, left). In the case of conventional tACS, we applied two currents at 5 Hz frequency and 1 mA amplitude. For an explanation for normalizing the exposure simulations to 1 mA instead of 2 mA current as used for the in vivo experiments, see ‘Reference head model’ section. Each stimulation condition was applied for 25 s. The 3D location of the electrodes within the expected mesial temporal anatomical targets was confirmed by a computed tomography (CT) of the head at the end of the experiment.

The recorded data were analyzed using a custom-written script in MATLAB (Mathworks). The electric potential signals were first bandpass filtered using a first-order Butterworth filter with cutoff frequencies of 0.5 kHz and 5 kHz in the case of TI stimulation and 1 Hz and 40 Hz in the case of conventional tACS. The normalized envelope modulation amplitude in each electrode was estimated by first extracting the recorded signal’s envelope waveform using a Hilbert transform and a low-pass filter (that is, first-order Butterworth filter with a cutoff frequency of 0.5 kHz) and then computing the mean half difference between the waveform maxima and minima (averaged first in 1 s epochs and then across the 25 epochs). The envelope modulation amplitude of each electrode was then normalized to the largest envelope modulation amplitude across the electrode’s contact points. The envelope modulation ratio was estimated by dividing the amplitude of the envelope waveform by the maximum signal amplitude. The field’s envelope modulation amplitude along the axis of the recording electrodes (that is, perpendicular to the hippocampal longitudinal axis) was estimated by computing the difference in envelope modulation amplitudes between neighboring contact points and dividing the value by the inter-contact distance. The normalized absolute amplitude in each electrode was estimated by computing the median value of the signal maxima (again averaged first in 1 s epochs and then across the 25 epochs). The field’s amplitude along the axis of the recording electrodes (that is, perpendicular to the hippocampal longitudinal axis) was estimated by computing the difference in amplitudes between neighboring contact points and dividing the value by the inter-contact distance. The spatial maps of the normalized envelope modulation amplitude and normalized absolute amplitude (Supplementary Fig. 2) were created by first applying a 3-point moving average over the electrode contacts followed by a linear interpolation of the electrode contact values in an 100 × 151 grid.

Hilbert transform was applied to TI data, followed by low-pass filtering (500 Hz) using a first-order zero-phase, forward–reverse Butterworth filter. Maximum and minimum amplitudes were computed by calculating the median values extracted from 1 s epochs. The amplitude envelope modulation for TI data was calculated using the average of the maximum and minimum amplitudes. The envelope modulation ratio was calculated as the ratio of the envelope modulation amplitude to the maximum amplitude. Field strengths were calculated using the first spatial derivative of the envelope modulation amplitude or maximum amplitude.

### Human subjects—in vivo experiments

Twenty-two healthy volunteers were recruited for the MRI experiment and 21 for the experiment probing the effects of TI stimulation on behavior. In the fMRI experiment, two participants were excluded, one because of technical difficulties with the MRI scanner (no images were collected) and another due to excessive movement in the scanner. Thus, the final cohort for this experiment was composed of twenty subjects (11 females, age range: 20–54 years old, mean age ± s.d. 27.1 ± 7.6 years, 19 right-handed and 1 left-handed). For the behavioral experiment, all participants were included in the analysis (10 females, age range: 19–30 years old, mean age ± s.d. 22.8 ± 3.2 years, all right-handed). All subjects were educated to degree level or above with no self-reported history of neurological or psychiatric illness. No statistical methods were used to predetermine sample sizes, but our sample sizes are similar to those reported in previous publications^24,25,29,34^. Data collection and analysis were not performed blind to the conditions of the experiments.

### Face–name task

The task was designed using the Psychtoolbox^74^ for MATLAB (Mathworks). In the MRI experiments, responses were collected with a response box (NordicNeuroLab) that was connected to the stimulus presentation computer through a fiberoptic cable. In the behavioral experiment, responses were collected using a computer keyboard connected to the stimulus presentation computer. The order of the stimulation was counterbalanced across participants using a balanced Latin square. This allowed us to keep factors of no interest fixed (that is, difficulty of a specific block or tiredness), while controlling for the variable of interest, that is, stimulation condition.

The face–name task was chosen on the basis of a strong body of evidence demonstrating that face–name associations are dependent on hippocampal function and elicit bilateral hippocampal activations in healthy subjects^21,25,26^. Faces were retrieved from the Chicago Face Database v.2.0.3 (ref. ^67^) and names from the Office for National Statistics (Baby Names, England and Wales, 1996; which corresponds to the dataset closest to the mean age for the faces in the Chicago Face Database, mean age 28 years). We selected names that had between five and seven letters. Names present in both female and male lists were removed (for example, Charlie), and if the same name was present with a different spelling (for example, Elliot and Elliott) only the one with the highest frequency was kept. The task was composed of 9 blocks in the fMRI experiment and 12 blocks (per session) in the behavioral experiment, each containing 16 unique face–name pairs of different ethnicities (4 Black female, 4 Black male, 4 white female and 4 white male per block; all with neutral facial expressions). The task was composed of an encoding and a recall stage. During the encoding stage each face–name pair was displayed for 2 s. Faces were displayed in the center of the screen with the name underneath (Fig. 2a). Participants were instructed to read the name underneath the faces and try to learn each face–name pair. This was followed by either a delay period (16 s) where a fixation cross was present in the center of the screen in the fMRI experiment, or a distractor task (40 s) where participants made odd/even judgments for random integers ranging from 1 to 99 (to prevent maintenance of information in working memory) in the behavioral experiment. In the recall stage, participants were shown each face with five names underneath: the target name, two distractor names (that is, names that were not present in the block) and two foil names (that is, names that were present in the block but associated with a different face)—target and distractor names were selected to have a similar name frequency. Each name appeared in black font, inside a gray rectangle and the temporally selected rectangle was colored in cyan. Participants moved between rectangles (using left and right index buttons in the fMRI experiment or left and right arrow keys in the behavioral experiment) and pressed a key to confirm their selection (right thumb in the fMRI or space bar in the behavioral experiment). This was followed by a confidence rating, in which participants were asked to rate how confident they were in their selection from 1 to 4 (1 being not confident at all and 4 extremely confident). Selection was made using the same procedure described for the name selection. For the recall stage, participants were instructed to respond as quickly and as accurately as possible. There was a time limit (20 s in the fMRI experiment and 8 s in the behavioral experiment) to select each name and to rate the confidence level (5 s in the fMRI experiment and 3 s in the behavioral experiment). Each block (and session for the behavioral experiment) contained unique stimuli. The order of the blocks was kept constant across participants, but the order of the face–name pairs was pseudo-randomized across participants, such that no more than three faces of the same type appeared in a row. The order of face recall was randomized across participants, and the last two encoding trials were never presented at the beginning of the recall. The position of the names in the recall stage was also randomized. In the behavioral experiment, participants performed a re-test, 30 min after the last recall block. During this period participants were asked to recall the names matching all faces presented for that session. Stimuli were grouped in their original blocks, but blocks presented in a different order from the recall after encoding and the order of the stimuli randomized per block. The order of the blocks in the re-test was kept constant between sessions. Confidence ratings were not collected during re-test. Participants watched a nature video between the last block of the recall period and the re-test. There was one video per session, and all participants watched the same videos.

### Behavioral analyses

Three main variables of interest were analyzed, that is, response type, reaction time for name selection and confidence level (reaction times for confidence were also recorded but not analyzed). For each face–name trial, four response types were possible at the recall stage: (1) correct association, selection of the correct name for the face presented; (2) foil (incorrect association), selection of a name that was present in the same block but did not match the face (two foils were present per recall trial); (3) distractor (incorrect association), selection of a name that was not associated to any face across all blocks (two distractors were present per recall trial); (4) omission, participant did not select a response within the time limit.

We first assessed accuracy across the whole task (correct versus incorrect associations) to check whether any participant had an overall performance below chance (20%), which would exclude them from further analyses. All participants were above chance (fMRI experiment: mean ± s.d. 49.97 ± 9.77%, range 32.64–70.83%; behavioral experiment—recall: 58.47 ± 14.73%, range 33.85–91.15%). The distribution of responses for correct associations, foils and distractors across the whole task followed the expected pattern, with higher percentage of responses for correct associations than foils than distractors, indicating appropriate engagement with the task (fMRI experiment: correct associations 50%, foil 33.3%, distractor 16.5%, omission 0.17%; behavioral experiment—recall: correct associations 58.46%, foil 24.76%, distractor 12.29%, omission 4.49%). The number of omissions was considered negligent and removed from the dataset. We then plotted reaction times across the whole task; this showed a right-skewed distribution, typical for this metric. To trim the distribution, we calculated the 1st and 99th percentiles across all trials and participants and dropped trials below or above these thresholds.

To investigate whether the number of responses differed per response type across stimulation conditions we performed a multinominal logistic regression on the trial-by-trial data using the multinom function in the net package in R (ref. ^75^). The outcome variable response type contained three levels, target, foil and distractor, and the level ‘target’ was used as the intercept, with predictors for stimulation type (sham, TI 1:1, TI 1:3 in the fMRI experiment and sham and TI 1:3 in the behavioral experiment), *P* values were calculated using Wald tests. In addition, as we were interested in investigating the contrasts for correct and incorrect responses in the imaging data, we defined a binomial GLMM using a logistic link function to model the effect of stimulation type on accuracy (correct versus incorrect associations). The final model included random intercepts for participant and task block (task block was not a variable of interest, as blocks were counterbalanced across stimulation conditions and thus included for appropriately modeling variance in the data). In the behavioral experiment, we included in addition random intercepts for session, and modeled accuracy used Bayesian mixed-effects models using the brms package^76^. Bayesian frameworks are robust to potential violations of normality or homoscedasticity and allow considering whether an effect is credibly different from a null value. The Bayesian model included random intercepts for participants, sessions and blocks.

To investigate whether reaction times differed per response type across stimulation conditions the data were modeled with GLMM using an inverse Gaussian distribution with the identity link function^77^. The final model included random intercepts for participants and blocks in the fMRI experiment and for participants, session and trial nested in blocks in the behavioral experiment. We also run an additional model using accuracy instead of response type, to investigate whether reaction times differed by accuracy and stimulation type (again employing the inverse Gaussian distribution with the identity link function and random intercepts as described above).

Finally, we investigated the participants confidence levels for the selected face–name associations. First, we removed trials where participants did not specify a confidence level within the time limit (fMRI experiment: 0.28% trials; behavioral experiment: 0.78% trials). To investigate whether confidence levels (ordinal variable) differed per response type across stimulation conditions, the data were modeled using a cumulative link mixed model (logit link function) using the ‘clmm’ function from the ordinal package in R (ref. ^78^).

### Statistical procedures

All statistical analyses were conducted using R version 3.6.0 via RStudio, and plots were generated with the ggplot2 package. GLMM models used the glmer function and LMM models the lmer function, from the lme4 package with *P* value approximation performed by the lmerTest package in R (refs. ^79,80^). Model suitability was evaluated using the residual diagnostics tool from the DHARMa package^81^. Bayesian models were implemented using the brms package^76^. We ran a minimum of 2,000 iterations over four Markov Chain Monte Carlo (MCMC) chains, with a ‘warm-up’ period of 1000 iterations per chain leading to 4000 usable posterior samples, visual inspection of all MCMC results revealed satisfactory Rhat values (<1.01). In these analyses, an effect is seen as statistically significant if the credible interval does not contain zero with 95% certainty.

### TI stimulation

TI stimulation was delivered using a custom-made device as described in ref. ^14^. Two sinusoidal waveforms (at frequencies 2 kHz and 2.005 kHz) were supplied via a balanced pair of current sources that were driven in precisely opposite phase with a ground electrode carrying any imbalance currents (<1%) from the paired current sources, preventing charging of the body relative to earth ground. Two pairs of stimulation electrodes (self-adhesive TENS, 1.5 cm × 1.5 cm with the corners cut to produce a rounded shape), were positioned on the participants’ heads using a conductive paste (Ten20, D.O. Weaver) and held in place using medical tape (3M Micropore medical tape). Electrode 1 (e_1_) and electrode 3 (e_3_) were positioned on the left hemisphere at the level of the nasion plane, e_1_ was positioned anterior to e_3_ (e_1_ at 50% of the subject’s half circumference minus 2.5 cm and e_3_ at 50% of the subject’s half circumference plus 2.5 cm, both counting from the nasion; such that the centers of the electrodes were 5 cm apart). Electrodes 2 and 4 (e_2_ and e_4_) were positioned on the right hemisphere at a plane just above the eyebrow, e_2_ was positioned anterior to e_4_ (e_2_ at 20% of the subject’s half circumference minus 1 cm and e_4_ at 70% of the subject’s half circumference plus 1 cm, both counting from the nasion). e_1_–e_2_ formed one electrode pair and e_3_–e_4_ the second electrode pair (Fig. 1b).

Stimulation was applied in two conditions: (1) TI stimulation directed to the mid left hippocampus: a current of 2 mA (peak to baseline) was applied to both electrode pairs, that is, a current ratio 1:1 (‘TI 1:1’ condition); (2) TI stimulation steered to the anterior left hippocampus: a current of 1 mA was applied to the electrode pair e_1_–e_2_ and a current of 3 mA (peak to baseline) was applied to the electrode pair e_3_–e_4_, that is, a current ratio 1:3 (‘TI 1:3’ condition). In both conditions, the stimulation began with a 5 s ramp-up and ended with a 5 s ramp-down. Sham stimulation was equivalent to the TI 1:1 stimulation condition in the fMRI experiments, but the current was ramped down to zero immediately after it was ramped up. In the behavioral experiment, sham stimulation was equivalent to the TI 1:3 condition, with an initial period of 30 s of stimulation followed by ramp-up and ramp-down periods before the first block of the face–name task and at the beginning and end of four consecutive blocks of the face–name task (Supplementary Fig. 9). The duration of stimulation was kept constant across participants for the fMRI experiment (96 s per stimulation condition), but durations varied between participants for the behavioral experiment where TI was applied throughout the face–name task blocks and responses were self-paced (stimulation during face–name task: mean ± s.d. 34.5 ± 3 min, range 29.37–40.35 min; total stimulation duration TI session: 44.53 ± 3 min, range 39.37–50.35 min, which includes 10 min of stimulation before the face–name task, 5 min during rest and 5 min during a general alertness task; Supplementary Fig. 9).

The beginning and end of each stimulation block/period was controlled via an external trigger, sent from the computer running the experimental paradigm to the stimulator (triggers were sent from MATLAB using serial port commands). In the fMRI experiment, TI stimulation was delivered in one session and in the same run of the fMRI task. The start of each block of the task was triggered by a signal from the magnetic resonance (MR) scanner; this ensured that task and stimulation were synchronous to the scanner clock. The stimulator was placed outside the MR shielded room, and the currents from the stimulator were delivered into the scanner room via radio frequency (RF) filters, one placed in the operator room and another inside the scanner bore (NeuroConn GmbH). The filter inside the MRI bore was connected to the stimulation electrodes via two MR-compatible stimulation cables (NeuroConn GmbH). Phantom and pilot experiments were initially conducted to ensure that the experimental setup did not introduce artifacts in the fMRI signal. Additionally, we estimated temporal signal-to-noise ratio (tSNR) in the fMRI signal in the brain regions underneath the electrodes on the left hemisphere and their contralateral equivalents (that is, ROIs) to assess whether the presence of the electrodes had an effect on the quality of the signal. Temporal signal-to-noise ratio was calculated by dividing the mean of the signal over time by the s.d. over the whole fMRI acquisition (Supplementary Fig. 10).

### Brain stimulation procedure

Before each experiment, the participants’ sensation and comfort were tested. Participants were first exposed to low-frequency stimulation followed by TI stimulation, for each electrode pair at a time, first e_1_–e_2_ followed by e_3_–e_4_. Stimulation started at 0.1 mA and increased in steps of 0.1 mA until participants reported any sensations associated to stimulation (that is, pins and needles, burning, phosphenes and so on) or until maximum intensities for the experimental protocol were reached (2 mA for e_1_–e_2_ and 3 mA for e_3_–e_4_). Participants responses were collected electronically by the experimenter using Microsoft Excel. A detailed description of perceptual sensations and thresholds is provided in Supplementary Tables 21–23. At the end of the experiments, participants completed a questionnaire (using pen and paper or an editable form in pdf format in Adobe Acrobat Reader) to assess possible side effects of TI stimulation by rating from 0 (none) to 4 (severe) the intensity and duration of: pain, burning, warmth/heat, itchiness, pitching, metallic taste, fatigue, effect on performance, trouble concentrating, sleepiness/fatigue, headache, mood change or any other side effect perceived. A detailed description of side effects, their intensity and the number of incidences is reported in Supplementary Tables 19 and 20. In the behavioral experiment we compared the strength ratings of the side effects using Wilcoxon signed-rank tests performed using the coin package^82^. A summary of methodological details associated to the simultaneous TI-fMRI procedure is reported according to the ContES checklist^83^ in Supplementary Table 25.

### Effectiveness of sham blinding

In the behavioral experiment, where we had separate active and sham sessions, we included an additional protocol to investigate the effectiveness of sham blinding by asking at four time points during each session the following questions: ‘Do you think you had stimulation?’ (yes/no) and ‘How confident are you?’ (1 is not confident at all; 10 is extremely confident). Participants responded to each question by using an editable form in pdf format in Adobe Acrobat Reader. Following Greinacher et al.^84^, we combined the two questions into a weighted score, whereby a ‘yes’ answer was assigned a +1 value and ‘no’ answer a value of −1, which were then multiplied by the confidence rating. We extracted the median and 95% confidence intervals for each time point and each stimulation condition using a smooth bootstrap technique^85^ implemented in the kernelboot package^86^. We used a Gaussian kernel and 10,000 permutations for each probe point.

### MRI data acquisition

Scanning was performed in a 3T Siemens Verio (Siemens) at the Imperial College’s CIF, using a 32-channel head coil. Standard T1-weighted structural images were acquired using a magnetization-prepared rapid gradient-echo (MP-RAGE) sequence, 1 mm^3^ isotropic voxel, repetition time (TR) 2.3 s, echo time (TE) 2.98 ms, inversion time 900 ms, flip angle (FA) 9°, field of view 256 × 256 mm, 256 × 256 matrix, 160 slices, GRAPPA acceleration factor 2. Field map scans were acquired to correct the echoplanar imaging images for signal distortion (TR = 599 ms, TE = 7.65 ms, FA = 60°). FMRI images were obtained using a T2*-weighted gradient-echo echoplanar imaging sequence, 3 mm^3^ isotropic voxel, TR 2 s, TE 30 ms, FA 80°, field of view 192 × 192 × 105 mm, 35 slices, GRAPPA acceleration factor 2. A total of 804 volumes were acquired on average (range 592–1,162); times varied depending on how long participants took on the recall stage of the task.

### ROIs

ROIs included: (1) the hippocampi, (2) their longitudinal parcellations, (3) regions corresponding to the AT–PM networks and (4) regions corresponding to the cortical regions overlying the stimulated hippocampus, that is, underneath all stimulating electrodes and between the stimulating electrodes e_1_ and e_3_.

Hippocampal masks were defined on the basis of the segmentation of the whole hippocampi performed for each subject using the pipeline for automated hippocampal subfield segmentation in FreeSurfer (version 6.0.0 (refs. ^87–89^)). The hippocampal masks were normalized to Montreal Neurological Institute (MNI) space and split into thirds along the long axis of the hippocampus^90^ (posterior portion of the hippocampus: from *Y* = −40 to −30; mid-portion of the hippocampus: from *Y* = −29 to −19; anterior portion of the hippocampus: from *Y* = −18 to −4). The inverse normalization parameters were used to create subject-specific parcellated ROIs and used in the subject space for fMRI analyses.

ROIs for the AT–PM networks were defined following^90^ using regions previously identified as belonging to distinct networks through resting state and FC analyses during associative memory encoding^91,92^. AT regions included the bilateral perirhinal cortex, amygdala, anterior fusiform gyrus, anterior inferior temporal cortex and lateral orbitofrontal cortex. PM regions included the parahippocampal cortex, posterior cingulate cortex, precuneus and angular gyrus. The ROIs were obtained from probabilistic atlases thresholded at 50%, including a medial temporal lobe atlas^93,94^ for parahippocampal cortex and precuneus, and the Harvard-Oxford cortical and subcortical atlases for all other regions (Fig. 4a).

ROIs for the cortex overlying the stimulated hippocampus were defined for each subject using the anatomical T1 images. All cortex ROIs were 10 mm spherical masks. See Fig. 1d for ROIs in the left hemisphere and Supplementary Fig. 5 for ROIs in the right hemisphere.

We extracted two additional sets of ROIs for control measurements: the left amygdala (using a procedure analogous to the individual hippocampal ROIs), the left temporal lobe (excluding the hippocampus) and additional cortical ROIs in the right hemisphere (same axial plane as the left hemisphere cortical ROIs). All ROIs were converted to the subject space for fMRI analyses.

### fMRI data preprocessing

Data were preprocessed using the FMRIB Software Library (FSL version 6.0.1 (refs. ^95,96^)). Functional data were preprocessed using the FMRI Expert Analysis Tool (FEAT), including motion correction using MCFLIRT^97^, distortion correction using fieldmap images prepared from fsl_prepare_fieldmap, slice-time correction using Slicetimer, smoothing with a 3D Gaussian kernel (8 mm full-width at half maximum) and high-pass filtered at a cutoff of 0.008 Hz. Skull stripping was performed using FSL’s BET^98^. Head motion was estimated using FSL motion outliers through DVARS (the spatial root mean square of the data after temporal differencing)^99^. Criterion for excessive motion was DVARS >0.5 in more than 20% of the volumes. In the fMRI experiment, one subject was excluded on this basis. For the sample included in the analyses, mean DVARS and s.d. were 0.25 and 0.03, respectively. There was no difference in motion across stimulation conditions (*F*_(2,38)_ = 1.03, *P* = 0.367).

### fMRI analysis

For each participant, preprocessed fMRI data were modeled using three different GLMs, two designed for univariate analyses and a third for assessing FC using generalized psychophysiological interaction (gPPI)^100^. In addition to the explanatory variables (EVs) of interest (described below), all GLMs included as nuisance regressors 24 motion parameters (6 motion parameters—translation and rotation in three directions, the square of the six motion parameters and their temporal derivatives) and a regressor with volume outliers identified by DVARS to model out volumes (that is, scrubbing) with extensive motion.

The first GLM was used to analyze univariate BOLD effects during encode and recall periods of the task and included three EVs for encode and three EVS for recall (one EV per stimulation condition and task stage) and their first temporal derivatives. Regressors were created by convolving a boxcar kernel with a canonical double-gamma hemodynamic response function.

The second GLM analyzed univariate BOLD effects for correct and incorrect trials during encode and recall periods. This was possible without temporal jittering because we obtained a balanced distribution between correct and incorrect responses and the ordering of trials was randomized as a consequence of subject performance and pseudo-randomization of the stimuli presentation across participants^101^. This model included 12 EVs (one for correct and another for incorrect trials for encode and recall periods per stimulation condition), 3 EVs for the confidence intervals (one per stimulation condition) and their first temporal derivatives. Regressors were created by convolving a boxcar kernel with a canonical double-gamma hemodynamic response function.

The third set of GLMs were used to assess FC. We used a gPPI method to quantify the effective connectivity for the contrast correct > incorrect, using the Ant, Mid and Post regions of the left hippocampus as seeds and the AT and PM network as targets. The gPPI allowed us to quantify the directional connectivity between the seeds and targets while accounting for task-unrelated connectivity and task-related activity. The gPPI models included 25 EVs, describing physiological, psychological and PPI regressors. Physiological regressors were defined from the fMRI time course extracted from seeds in the Ant, Mid and Post left hippocampus (see ROI definition). The psychological regressors included those modeled for the second GLM. For each model (one per seed), the physiological term and the psychological term were used to create the PPI interaction terms.

Using the output of the first GLM we assessed the fMRI BOLD signal to the encode and recall periods of the task (contrasted against the baseline), first for the sham condition (Fig. 2) and then for each stimulation condition (Fig. 3). Using the output of the second GLM, we measured BOLD response to correct and incorrect associations during the encode period of the task (contrasted against the baseline), first for the sham condition and then for all stimulation conditions (EVs 1–12). The contrast correct > incorrect was also used to extract connectivity values in the gPPI models described above.

### fMRI statistics

Whole brain BOLD activity at the group level was visualized by employing mixed-effects analyses using FLAME 1 (refs. ^102,103^). *Z* statistical images were thresholded using Gaussian random fields-based cluster inference with an initial cluster-forming threshold of *Z* > 3.1 and an FWE-corrected cluster-extent threshold of *P* < 0.05.

Statistical analyses of fMRI data were performed using the model estimates (in percent BOLD signal change) from the ROIs defined a priori. For each subject, we employed FSL Featquery tool to interrogate time-series-associated statistics, for each of the contrasts defined above, in the ROIs in the subject space (see ROI specification). For analysis of the BOLD magnitude, we used the median % BOLD signal change (as the mean values were often not normally distributed) and for connectivity analysis we used the means.

ROI statistical analyses were conducted using R version 3.6.0, and plots were generated with the ggplot2 package. All mixed-effects models were fitted using the function lmer from the lme4 package in R. ANOVA Type II Wald *F* tests with Kenward–Roger approximation for degrees of freedom or Type II Wald chi-square tests were performed using the function Anova() for *P*-value approximation. Post hoc Tukey’s comparisons were made using the estimated marginal means from the emmeans package. The level of statistical significance was set at *P* < 0.05 for all tests.

Voxelwise analyses within ROIs were performed using FSL’s randomise tool with 5,000 permutations and FWE correction for multiple comparisons using threshold-free cluster enhancement. All statistical maps were FWE corrected and thresholded at *P* < 0.05.

### Reporting summary

Further information on research design is available in the Nature Portfolio Reporting Summary linked to this article.

## Online content

Any methods, additional references, Nature Portfolio reporting summaries, source data, extended data, supplementary information, acknowledgements, peer review information; details of author contributions and competing interests; and statements of data and code availability are available at 10.1038/s41593-023-01456-8.

### Supplementary information

Supplementary Information Supplementary Figs. 1–10 and Tables 1–25.

Reporting Summary

## Data Availability

Group-level data used to generate the fMRI figures are available in NeuroVault (https://neurovault.org/collections/11908/). A 3D viewer for visualization of the spatial distribution of the amplitude modulation magnitude (TI) and of the maximum carrier frequency electric field (HF) is available online (https://osparc.io/study/9641ba42-c4db-11ed-b8b9-02420a0b5f22). Faces used in the face–name task were retrieved from the Chicago Face Database v.2.0.3 (ref. ^67^).

## Code availability

Data and key scripts are available on GitLab (https://gitlab.eps.surrey.ac.uk/nemo/ti-paper). The code for the face–name task is available on GitLab (https://gitlab.eps.surrey.ac.uk/nemo/facenametask).
