# Publisher Correction: Non-invasive temporal interference electrical stimulation of the human hippocampus

**DOI:** 10.1038/s41593-023-01517-y

**Published:** 2023-11-13

**Authors:** Ines R. Violante, Ketevan Alania, Antonino M. Cassarà, Esra Neufeld, Emma Acerbo, Romain Carron, Adam Williamson, Danielle L. Kurtin, Edward Rhodes, Adam Hampshire, Niels Kuster, Edward S. Boyden, Alvaro Pascual-Leone, Nir Grossman

**Affiliations:** 1 School of Psychology, Faculty of Health and Medical Sciences, University of Surrey, Guildford, UK; 2 Department of Brain Sciences, Imperial College London, London, UK; 3 UK Dementia Research Institute, Imperial College London, London, UK; 4 Foundation for Research on Information Technologies in Society (IT’IS), Zurich, Switzerland; 5 Institut de Neurosciences des Systèmes, Aix-Marseille University, INSERM, Marseille, France; 6 Department of Neurology and Neurosurgery, Emory University Hospital, Atlanta, GA USA; 7 Department of Functional and Stereotactic Neurosurgery, Timone University Hospital, Marseille, France; 8 International Clinical Research Center, St. Anne’s University Hospital and Faculty of Medicine, Masaryk University, Brno, Czech Republic; 9 Department of Information Technology and Electrical Engineering, Swiss Federal Institute of Technology, Zurich, Switzerland; 10 Departments of Brain and Cognitive Sciences, Media Arts and Sciences, and Biological Engineering, McGovern and Koch Institutes, Massachusetts Institute of Technology, Cambridge, MA USA; 11 Howard Hughes Medical Institute, Cambridge, MA USA; 12 Hinda and Arthur Marcus Institute for Aging Research and Deanna and Sidney Wolk Center for Memory Health, Hebrew SeniorLife, Boston, MA USA; 13 Department of Neurology, Harvard Medical School, Boston, MA USA

**Keywords:** Medical research, Neuroscience, Biological techniques

Correction to: *Nature Neuroscience* 10.1038/s41593-023-01456-8, published online 19 October 2023.

In the version of the article initially published, the first affiliation for Romain Carron was incorrect and has now been updated to Institut de Neurosciences des Systèmes, Aix-Marseille University, INSERM, Marseille, France in the HTML and PDF versions of the article.

