# Supplementary material for: Non-invasive temporal interference electrical stimulation of the human hippocampus
Source: Nat Neurosci. 2023 Oct 19;26(11):1994–2004. doi: 10.1038/s41593-023-01456-8 (PMC10620081; doi:10.1038/s41593-023-01456-8)
Supplement: Supplementary file 1 — Supplementary Figs. 1–10 and Tables 1–25. [file 41593_2023_1456_MOESM1_ESM.pdf]

nature neuroscience

Article

https://doi.org/10.1038/s41593-023-01456-8

# Non-invasive temporal interference electrical stimulation of the human hippocampus

In the format provided by the
authors and unedited

# Supplemental Information

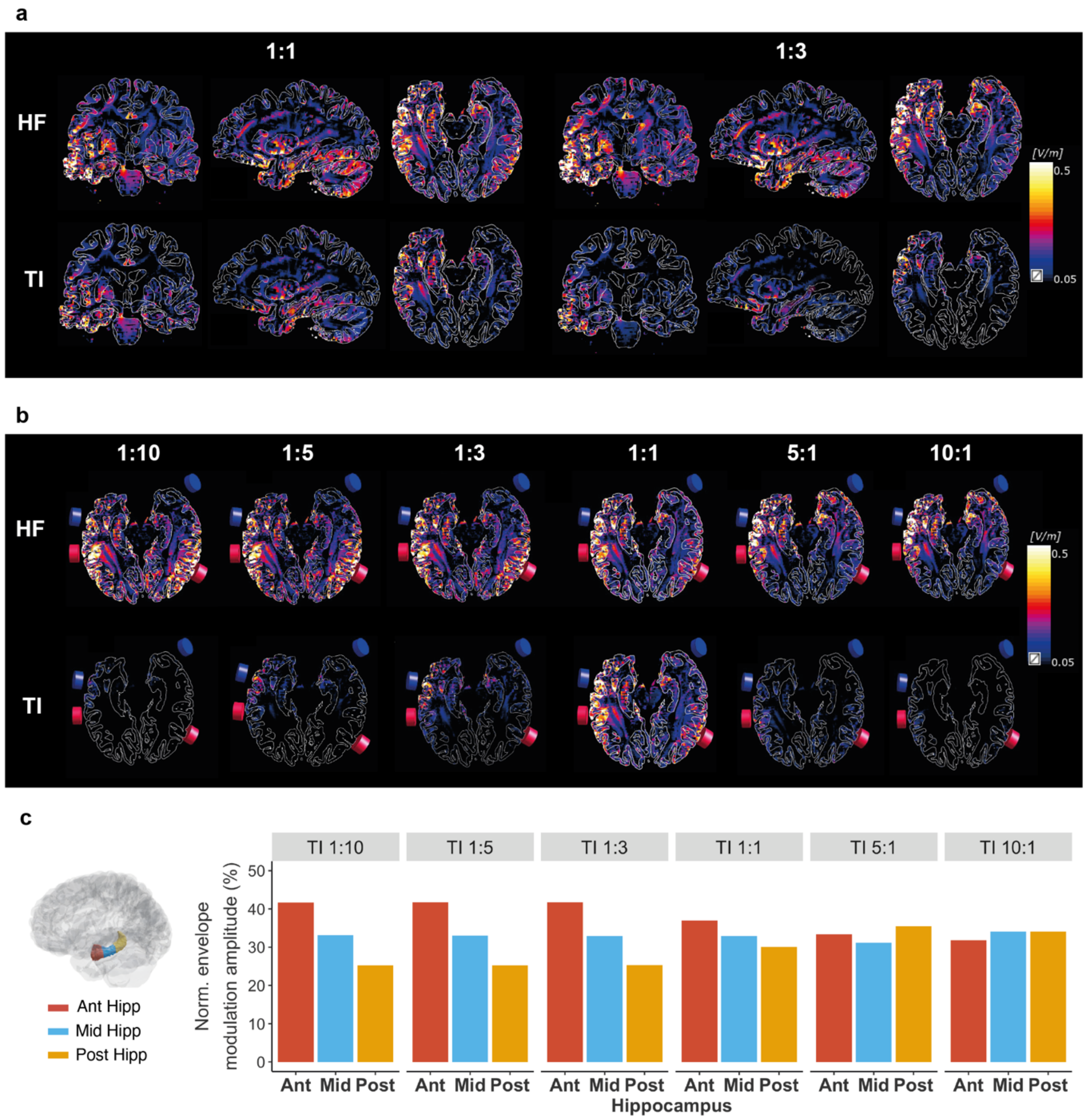


**Fig. S1: TI field distribution computed in the MIDA model.**
**a,** High frequency (HF, i.e. absolute amplitude) and TI (i.e. envelope modulation amplitude) fields showed across coronal, sagittal and axial planes for current ratios 1:1 and 1:3. All planes show slices that pass through the hippocampus and are shown in neurological orientation. A 3D viewer is available online [1].
**b,** High frequency (HF, i.e. absolute amplitude) and TI (i.e. envelope modulation amplitude) fields showed in an axial slice parallel to the hippocampal longitudinal axis for varying current ratios (total current kept fixed at 1 mA per electrode pair). Images are shown in neurological orientation.
**c**, Envelope modulation amplitude in hippocampal ROIs (ROI schematic shown on the left panel) for the current ratios shown in **b**; ROI amplitudes were normalised to total hippocampal exposure. Note how shifting the relative current amplitude between electrode pairs steers the envelope modulation to the field with lower amplitude, i.e. shifting from Ant (anterior) to the Post (posterior) hippocampus. In combination, panels **b** and **c** show that the TI can be steered

to more anterior or posterior portions of the hippocampus, but current ratios with very small current intensities in one electrode pair also result in a smaller envelope amplitude at the target region.

[1] - https://osparc.io/study/9641ba42-c4db-11ed-b8b9-02420a0b5f22

**Table S1 | Induced potential in human cadaver; related to Fig. 1g.**
Induced potential for contacts in cortical and hippocampal tissue measured for the envelope modulation amplitude and absolute amplitudes for electrodes *a-c* (see **Fig. 1g**). Superficial contacts across the three intracranial electrodes were assigned to 'cortex' tissue (7 contacts, contacts 15-9, 12-33 mm depth) and deeper contacts to 'hippocampal' (8 contacts, contacts 8-1, 36.5-61 mm depth) tissue. Values represent the median ± standard deviation (SD) averaged across electrodes per region. Linear mixed model (LMM) for envelope modulation amplitude and absolute amplitude, followed by post-hoc pairwise comparisons, two-sided. Models included the median envelope modulation amplitude (EnvModAmp) or the median absolute amplitude (AbsAmp) as the dependent variable, region (Cortex or Hippocampus) as independent variable, and random intercepts for electrodes (a, b, c). Shown are the Analysis of Deviance Tables (Type II Wald F tests with Kenward-Roger correction for degrees of freedom), generated by the Anova() function applied to the linear mixed models fitted in R, followed by the post-hoc contrasts, two-sided. [a]: Specification of the linear model fitted in the R language, Df, Degrees of freedom; Df.res, residual degrees of freedom; F, F-statistic; P: P-value; B, estimate; SE, standard error, t, t-statistic. Signif. codes: '***' 0.001, '**' 0.01, '*' 0.05, '.' 0.1.
Amplitudes in **Fig. 1g** have been normalised to hippocampal values.

| | **Cortex** | | **Hippocampus** | | |
|---|---|---|---|---|---|
| Envelope Modulation Amplitude mV | 0.35±0.38 | | 1.22±0.34 | | |
| Absolute Amplitude mV | 5.30±1.00 | | 3.49±0.23 | | |
| **Linear mixed model for Envelope Modulation Amplitude** | | | | | |
| **lmer(EnvModAmp ~ region + (1 \| electrode) [a]** | **Df** | **Df.res** | **F** | **P** | |
| region | 1 | 2 | 27.533 | 0.0345 * | |
| **Post-hoc contrasts** | | | | | |
| | **B** | **SE** | **df** | **t** | **p** |
| Cortex - Hippocampus | -0.877 | 0.0167 | 2 | -5.515 | 0.0345* |
| **Linear mixed model for Absolute Amplitude** | | | | | |
| **lmer(AbsAmp ~ region + (1 \| electrode) [a]** | **Df** | **Df.res** | **F** | **P** | |
| region | 1 | 2 | 49.723 | 0.01952 * | |
| **Post-hoc contrasts** | | | | | |
| | **B** | **SE** | **df** | **t** | **p** |
| Cortex - Hippocampus | 1.974 | 0.028 | 2 | 7.051 | 0.0195* |

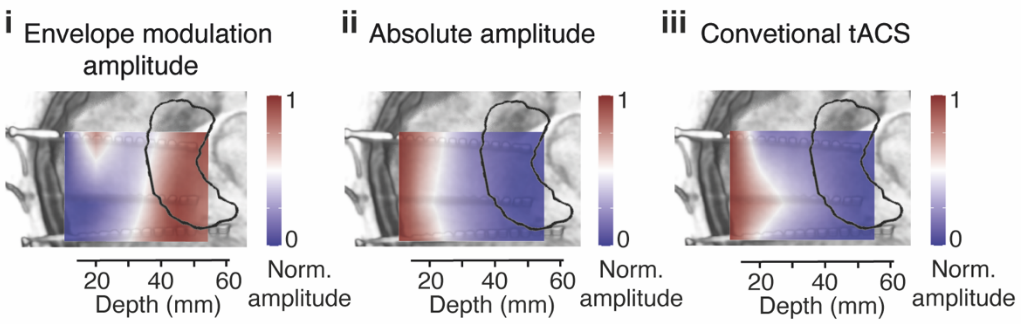


**Fig. S2: Measurements in human cadaver; related to Fig. 1.** Zoomed view of box region highlighted in **Fig. 1g** showing interpolated normalised amplitude maps of (**i**) envelope modulation amplitude map, (**ii**) absolute amplitude, (**iii**) absolute amplitude for conventional transcranial alternating current stimulation (tACS) at 5 Hz. Higher amplitudes are observed at the location of the hippocampus (black contour) for the envelope compared to absolute amplitude and conventional tACS.

**Table S2 | TI fields in the hippocampus; related to Fig. 2c.**
**S2.1**. Median and standard deviation (SD) for individualised electric field simulations based on participants' anatomical models, extracted from ROIs in the cortex and left hippocampus (see **Fig. 1d** for a schematic of the ROI locations). Shown are the envelope modulation and absolute amplitudes; N=16 (four subjects had to be excluded from the modelling since their electrodes were not visible in the MRI – see **Fig. S3**) for TI 1:1 and TI 1:3 stimulation conditions. **S2.2.** Statistical analyses on the normalised modulation envelope amplitudes for TI 1:1 and TI 1:3 stimulation conditions. Fields for each hippocampal region (**Fig. 2b)** were normalised to the whole hippocampus. Linear mixed model (LMM) for each stimulation condition. Models included the median normalised modulation envelope as the dependent variable (mdField), hippocampal regions (HippRg) as independent variable, and random intercepts for participants. Ant – anterior, Mid – middle, Post – posterior. Shown are the Analysis of Deviance Tables (Type II Wald F tests with Kenward-Roger correction for degrees of freedom), generated by the Anova() function applied to the linear mixed models fitted in R, followed by post-hoc contrasts using the Tukey HSD test, two-sided. [a]: Specification of the linear model fitted in the R language, Df, Degrees of freedom; Df.res, residual degrees of freedom; F, F-statistic; P: P-value; B, estimate; SE, standard error, t, t-statistic. Signif. codes: '***' 0.001, '**' 0.01, '*' 0.05, '.' 0.1.

| **S2.1 – Electric Field Simulation for Individualised Models** | | | |
|---|---|---|---|
| | | **Cortex** | **Hippocampus** |
| **TI 1:1** | Envelope Modulation Amplitude V/m | 0.23±0.18 | 0.40±0.05 |
| | Absolute Amplitude V/m | 0.46±0.25 | 0.47±0.05 |
| **TI 1:3** | Envelope Modulation Amplitude V/m | 0.19±0.15 | 0.24±0.03 |
| | Absolute Amplitude V/m | 0.45±0.26 | 0.47±0.05 |

| **S2.2 – Steering effect – Individualised Models** | | | | |
|---|---|---|---|---|
| **Linear mixed model for TI 1:1** | | | | |
| **lmer(mdField ~ HippRg + (1 \| ID)** [a] | **Df** | **Df.res** | **F** | **P** |
| Hippocampal regions (HippRg) | 2 | 30 | 26.045 | $2.77 \times 10^{-7}$ *** |
| **Post-hoc contrasts** | | | | |

| | B | SE | df | t | p |
|---|---|---|---|---|---|
| Ant - Mid | -0.0487 | 0.00884 | 30 | -5.515 | <.0001 * |
| Ant - Post | 0.0113 | 0.00884 | 30 | 1.274 | 0.4203 |
| Mid - Post | 0.0600 | 0.00884 | 30 | 6.789 | <.0001 * |
| **Linear mixed model for TI 1:3** | | | | | |
| **lmer(mdField ~ HippRg + (1 \| ID)** [a] | **Df** | **Df.res** | **F** | **P** | |
| Hippocampal regions (HippRg) | 2 | 30 | 359.62 | $< 2.2 \times 10^{-16}$ *** | |
| **Post-hoc contrasts** | | | | | |
| | **B** | **SE** | **df** | **t** | **p** |
| Ant - Mid | 0.0648 | 0.00552 | 30 | 11.741 | <.0001 * |
| Ant - Post | 0.1476 | 0.00552 | 30 | 26.752 | <.0001 * |
| Mid - Post | 0.0828 | 0.00552 | 30 | 15.011 | <.0001 * |

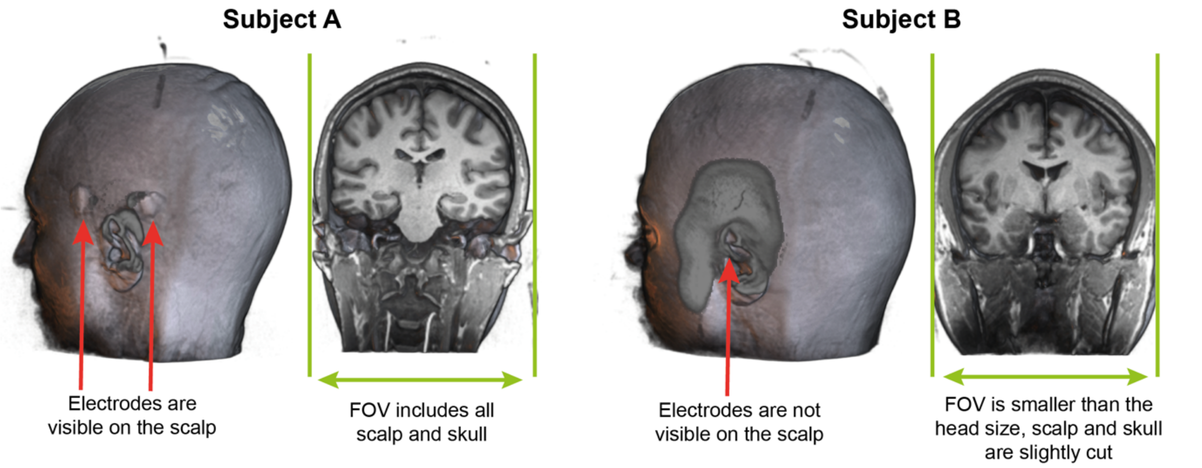


**Fig. S3. Constraints to individualised electric field simulations.** Anatomical 3D reconstructions and coronal slices showing a participant for which individualised electric field simulation was conducted (Subject A) and a participant for which this was not possible (Subject B). For 4 out of the 20 participants, their scalp and skull were not completely inside the field-of-view (FOV) causing the electrodes in the left hemisphere not to be visible in the anatomical MRI images and inaccurate scalp and skull segmentations.

**Table S3 | One sample t-tests for recall accuracy for face-name task performed during fMRI acquisition.**

One sample t-tests on the proportion of associations correctly recalled per stimulation condition. Statistical analyses were performed using one-sample t-tests (type "greater", i.e. one-sided, null value 0.2, which is the chance level, i.e. probability of selecting the target out of 5 possible responses). P-values corrected using False Discover Rate (FDR). Shown are the estimate; t, t-statistic; P, P-value; P(FDR), P-value corrected using FDR; N=20. Signif. codes: '***' 0.001, '**' 0.01, '*' 0.05, '.' 0.1.

| **Stimulation condition** | **t** | **P** | **P (FDR)** |
|---|---|---|---|
| Sham | 10.014 | $2.575 \times 10^{-9}$ *** | $2.575 \times 10^{-9}$ *** |
| TI 1:1 | 10.476 | $1.237 \times 10^{-9}$ *** | $1.856 \times 10^{-9}$ *** |
| TI 1:3 | 13.208 | $2.521 \times 10^{-11}$ *** | $7.563 \times 10^{-11}$ *** |

**Table S4 | Memory performance statistics, fMRI study; related to Fig. 2d**
Three main variables of interest were analysed to assess behavioural performance, i.e., response type – related to accuracy, reaction time for name selection and confidence level. Shown are the statistical models applied to each behavioural metric (Model), the Analysis of Deviance Tables (Type II Wald chisquare tests), generated by the Anova() function applied to the models fitted in R. [a]: Specification of the model fitted in the R language, Df, Degrees of freedom; $\chi^2$, Chi-square; P, P-value; N=20 (ID). Signif. codes: '***' 0.001, '**' 0.01, '*' 0.05, '.' 0.1.

| **Accuracy** | | | |
|---|---|---|---|
| **Multinomial logistic regression** | | | |
| **nnet::multinom(formula= Response ~ ST)[a]** | **Df** | $\chi^2$ | **P** |
| Stimulation Type (ST) | 4 | 2.4288 | 0.6574 |
| **Binomial logistic regression** | | | |
| **glmer(Accuracy ~ ST + (1\| ID) + (1\|block), family = binomial(link = "logit"))[a]** | **Df** | $\chi^2$ | **P** |
| Stimulation Type (ST) | 2 | 0.0583 | 0.9713 |
| **Reaction Time** | | | |
| **Generalised mixed linear model** | | | |
| **glmer(RT ~ ST* Response + (1\|ID) + (1\|block), family = inverse.gaussian(link=identity)[a]** | **Df** | $\chi^2$ | **P** |
| Stimulation Type (ST) | 2 | 1.0652 | 0.5871 |
| Response (Target, Foil, Distractor) | 2 | 68.242 | $1.518 \times 10^{-15}$ *** |
| Stimulation Type:Response | 4 | 6.8863 | 0.1420 |
| **Generalised mixed linear model - binomial** | | | |
| **glmer(RT ~ ST*Accuracy + (1\|ID) + (1\|block), family = inverse.gaussian(link=identity))[a]** | **Df** | $\chi^2$ | **P** |
| Stimulation Type (ST) | 2 | 1.0700 | 0.58567 |
| Accuracy (Correct, Incorrect) | 1 | 69.212 | $< 2 \times 10^{-16}$ *** |
| Stimulation Type:Accuracy | 2 | 5.1867 | 0.07477 |
| **Confidence** | | | |
| **Cumulative Link Mixed Model** | | | |
| **clmm(Confidence ~ ST*Response + (1\|ID) + (1\|block), Hess = TRUE)[a]** | **Df** | $\chi^2$ | **P** |
| Stimulation Type (ST) | 2 | 10.43 | 0.005433 ** |
| Response (Target, Foil, Distractor) | 2 | 420.54 | $< 2 \times 10^{-16}$ *** |
| Stimulation Type:Response | 4 | 5.39 | 0.249476 |

**Table S5 | BOLD fMRI signal evoked by face-name memory task during sham (i.e., no) stimulation in the hippocampus; related to Fig. 2f.**
**S5.1.** One sample t-tests on the median BOLD signal (% signal change) extracted from individual hippocampal masks during encode and recall stages of the task, in the absence of stimulation, i.e. sham, condition. Statistical analyses were performed using one-sample t-tests, two-sided. P-values corrected using False Discover Rate (FDR). Shown are the estimate; t, t-statistic; P, P-value; P(FDR), P-value corrected using FDR. **S5.2**. Linear mixed effects model, with median BOLD (mdBOLD) signal as the dependent variable, independent factors for hemisphere (H: left, right) and task stage (TS: encode, recall), and random intercepts for participants (ID). Shown are the Analysis of Deviance Table (Type II Wald F

tests with Kenward-Roger correction for degrees of freedom), generated by the Anova() function applied to the repeated measures analysis with mixed models fitted in R. [a]: Specification of the linear model fitted in the R language, Df, Degrees of freedom; Df.res, residual degrees of freedom; F, F-statistic; P, P-value; B, estimate; SE, standard error, t, t-statistic; N=20. Signif. codes: '***' 0.001, '**' 0.01, '*' 0.05, '.' 0.1.

| **S5.1 - One-sample t-test** | | | | |
|---|---|---|---|---|
| **Task Stage** | **Hemisphere** | **t** | **P** | **P (FDR)** |
| Encode | Left | 3.6981 | 0.00152 *** | 0.00305 ** |
| | Right | 3.9215 | 0.00091 *** | 0.00305 ** |
| Recall | Left | -1.2812 | 0.21553 | 0.2874 |
| | Right | -0.2503 | 0.80503 | 0.805 |

| **S5.2 - Repeated measures analysis with mixed model** | | | | |
|---|---|---|---|---|
| **lmer(mdBOLD ~ TS*H + (1 \| ID) [a]** | **Df** | **Df.res** | **F** | **P** |
| Task Stage (TS) | 1 | 57 | 20.4921 | $3.097 \times 10^{-5}$ *** |
| Hemisphere (H) | 1 | 57 | 0.5211 | 0.4733 |
| Task Stage: Hemisphere | 1 | 57 | 0.1328 | 0.7169 |

**Table S6 | BOLD fMRI signal evoked by face-name memory task during sham (i.e., no) stimulation in the segmented left hippocampus; related to Fig. 2g.**
LMM on the median BOLD signal (% signal change) extracted from individual segmentations of the left hippocampus during the encode stage of the task, in the absence of stimulation, i.e. sham, condition. Statistical analyses were performed using LMM, with median BOLD (mdBOLD) signal as the dependent variable, independent factors for ROI (Ant - anterior, Mid – mid, Post – posterior), and random intercepts for participants (ID). Shown are the Analysis of Deviance Table (Type II Wald F tests with Kenward-Roger correction for degrees of freedom), generated by the Anova() function applied to the repeated measures analysis with mixed models fitted in R, followed by the post-hoc contrasts for models with significant interactions using the Tukey HSD test, two-sided. [a]: Specification of the linear model fitted in the R language, Df, Degrees of freedom; Df.res, residual degrees of freedom; F, F-statistic; P, P-value; B, estimate; SE, standard error, t, t-statistic N=20. Signif. codes: '***' 0.001, '**' 0.01, '*' 0.05, '.' 0.1.

| **Repeated measures analysis with mixed model** | | | | | |
|---|---|---|---|---|---|
| **lmer(mdBOLD ~ ROI + (1 \| ID) [a]** | **Df** | **Df.res** | **F** | **P** | |
| ROI | 2 | 38 | 8.7171 | 0.0007658 *** | |
| **Post-hoc contrasts** | | | | | |
| | **B** | **SE** | **df** | **t** | **P** |
| Ant - Mid | 0.1294 | 0.0322 | 38 | 4.024 | 0.0008 *** |
| Ant - Post | 0.0957 | 0.0322 | 38 | 2.976 | 0.0137 * |
| Mid - Post | -0.0337 | 0.0322 | 38 | -1.048 | 0.5518 |

**Table S7 | BOLD fMRI signal evoked by face-name memory task during sham (i.e., no) stimulation in the segmented left hippocampus for the contrast correct > incorrect.**
**S7.1**. LMM for the median BOLD signal (% signal change) extracted from individual left hippocampal ROIs for fMRI Model 2, which differentiates between correct and incorrect

responses during the encode stage of the task. Median BOLD (mdBOLD) signal is the dependent variable, independent factors for ROI (Ant - anterior, Mid – mid, Post – posterior) and response type (RspType: correct, incorrect), and random intercepts for participants (ID). Followed by estimated marginal means and standard error (SE).
**S7.2**. Same as S7.1 but for the right hippocampus.
Shown are the Analysis of Deviance Table (Type II Wald F tests with Kenward-Roger correction for degrees of freedom), generated by the Anova() function applied to the repeated measures analysis with mixed models fitted in R, followed by presentation of the estimated marginal means for each factor for models with significant main effects. [a]: Specification of the linear model fitted in the R language, Df, Degrees of freedom; Df.res, residual degrees of freedom; F, F-statistic; P, P-value; Mean, estimated marginal mean; SE, standard error; N=20. Signif. codes: '***' 0.001, '**' 0.01, '*' 0.05, '.' 0.1.

| **S7.1 – Left Hippocampus** | | | | |
|---|---|---|---|---|
| **Repeated measures analysis with mixed model** | | | | |
| **lmer(mdBOLD ~ ROI*RspType + (1 \| ID)** [a] | **Df** | **Df.res** | **F** | **P** |
| ROI | 2 | 95 | 4.5808 | 0.012612 * |
| RspType | 1 | 95 | 11.0923 | 0.001235 ** |
| ROI: RspType | 2 | 95 | 0.9456 | 0.392065 |
| **Estimated Marginal Means** | | | | |
| **ROI** | **RspType** | **Mean** | | **SE** |
| Ant | Correct | 0.2748 | | 0.0547 |
| | Incorrect | 0.1151 | | 0.0547 |
| Mid | Correct | 0.1418 | | 0.0547 |
| | Incorrect | -0.0049 | | 0.0547 |
| Post | Correct | 0.1236 | | 0.0547 |
| | Incorrect | 0.0738 | | 0.0547 |
| **S7.2 – Right Hippocampus** | | | | |
| **Repeated measures analysis with mixed model** | | | | |
| **lmer(mdBOLD ~ ROI*RspType + (1 \| ID)** [a] | **Df** | **Df.res** | **F** | **P** |
| ROI | 2 | 95 | 0.1263 | 0.8815 |
| RspType | 1 | 95 | 1.3453 | 0.2490 |
| ROI: RspType | 2 | 95 | 0.1735 | 0.8410 |

**Table S8 | BOLD signal in the hippocampus across stimulation conditions; related to Fig. 3b and Fig. 3g.**
**S8.1**. BOLD signal (% signal change) for the left, i.e. stimulated, hippocampus during encode and recall stages of the task, for the three stimulation conditions: sham, TI 1:1 and TI 1:3 (**Fig. 3b**). Statistical analyses were performed using a linear mixed effects model (LMM), with median BOLD (mdBOLD) signal as the dependent variable, independent factors for stimulation type (ST: sham, TI 1:1, TI 1:3) and task stage (TS: encode, recall), and random intercepts for participants (ID), N=20. Post-hoc comparisons using the Tukey HSD test, wto-sided. **S8.2**. As per 8.1, but for the right, i.e. non-stimulated hippocampus; related to **Fig. 3g**; N=20**.** Signif. codes: '***' 0.001, '**' 0.01, '*' 0.05, '.' 0.1.

| **S8.1 – Left Hippocampus (stimulated hippocampus)** | | | | |
|---|---|---|---|---|
| **Repeated measures analysis with mixed model** | | | | |
| **lmer(mdBOLD ~ ST*TS + (1 \| ID)** [a] | **Df** | **Df.res** | **F** | **P** |
| Stimulation Type (ST) | 2 | 95 | 3.2224 | 0.04425 * |

| | | | | | |
|---|---|---|---|---|---|
| Task Stage (TS) | 1 | 95 | 44.8436 | $1.492 \times 10^{-9}$ *** | |
| Stimulation Type:Task Stage | 2 | 95 | 2.9611 | 0.05656 . | |
| **Post-hoc contrasts** | | | | | |
| | **B** | **SE** | **df** | **t** | **P** |
| Task Stage = Encode | | | | | |
| Sham - TI 1:1 | 0.0124 | 0.042 | 95 | 0.296 | 0.9529 |
| Sham - TI 1:3 | 0.1317 | 0.042 | 95 | 3.133 | 0.0065 * |
| TI 1:1 - TI 1:3 | 0.1192 | 0.042 | 95 | 2.837 | 0.0153 * |
| Task Stage = Recall | | | | | |
| Sham - TI 1:1 | 0.0266 | 0.042 | 95 | 0.634 | 0.8020 |
| Sham - TI 1:3 | 0.0141 | 0.042 | 95 | 0.335 | 0.9399 |
| TI 1:1 - TI 1:3 | -0.0125 | 0.042 | 95 | -0.298 | 0.9522 |
| **S8.2 – Right Hippocampus** | | | | | |
| **Repeated measures analysis with mixed model** | | | | | |
| **lmer(mdBOLD ~ ST*TS + (1 \| ID)** [a] | **Df** | **Df.res** | **F** | **P** | |
| Stimulation Type (ST) | 2 | 95 | 0.7244 | 0.4873 | |
| Task Stage (TS) | 1 | 95 | 18.3715 | $4.359 \times 10^{-5}$ *** | |
| Stimulation Type:Task Stage | 2 | 95 | 2.0744 | 0.1313 | |

**Table S9 | BOLD signal in the segmented hippocampus across stimulation conditions; related to Fig. 3c.**
BOLD signal (% signal change) extracted from individual hippocampal segments during the encode stage of the task, for the three stimulation conditions: sham, TI 1:1 and TI 1:3. Statistical analyses were performed using a linear mixed effects model, with median BOLD (mdBOLD) signal as the dependent variable, independent factors for stimulation type (ST: sham, TI 1:1, TI 1:3) and ROI (Ant - anterior, Mid – mid, Post – posterior), and random intercepts for participants (ID), N=20. Shown are the Analysis of Deviance Table (Type II Wald F tests with Kenward-Roger correction for degrees of freedom), generated by the Anova() function applied to the repeated measures analysis with mixed models fitted in R, followed by estimated marginal means for each factor for models with significant main effects and difference to Sham condition. [a]: Specification of the linear model fitted in the R language, Df, Degrees of freedom; Df.res, residual degrees of freedom; F, F-statistic; P, P-value; Mean, estimated marginal mean; SE, standard error; N=20. Signif. codes: '***' 0.001, '**' 0.01, '*' 0.05, '.' 0.1.

| | | | | |
|---|---|---|---|---|
| **Repeated measures analysis with mixed model** | | | | |
| **lmer(mdBOLD ~ ST*ROI + (1 \| ID)** [a] | **Df** | **Df.res** | **F** | **P** |
| Stimulation Type (ST) | 2 | 152 | 12.6459 | $8.313 \times 10^{-6}$ *** |
| ROI | 2 | 152 | 6.3506 | 0.002245 ** |
| Stimulation Type:ROI | 4 | 152 | 0.4639 | 0.762166 |
| **Estimated Marginal Means and Relative Differences to Sham** | | | | |
| | **Mean** | **SE** | | **Mean – Sham** |
| **ROI = Ant** | | | | |
| Sham | 0.2015 | 0.0501 | | - |
| TI 1:1 | 0.1931 | 0.0501 | | 0.0084 |
| TI 1:3 | 0.0247 | 0.0501 | | 0.1770 |
| **ROI = Mid** | | | | |
| Sham | 0.0721 | 0.0501 | | - |
| TI 1:1 | 0.0846 | 0.0501 | | -0.0124 |
| TI 1:3 | -0.0192 | 0.0501 | | 0.0913 |

| | | | |
|---|---|---|---|
| **ROI = Post** | | | |
| Sham | 0.1058 | 0.0501 | - |
| TI 1:1 | 0.0713 | 0.0501 | 0.0345 |
| TI 1:3 | -0.0333 | 0.0501 | 0.1391 |

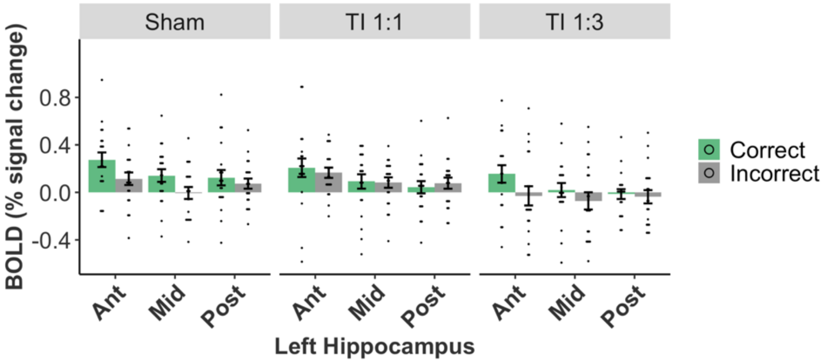


**Fig. S4: Comparison of group median change in BOLD signal between correct and incorrect encoded associations for each stimulation condition in the left hippocampus; related to Fig. 3e.** See **Table S7** and **Table S10** for full statistics. Bar plots show mean and standard error (SE), black dots show individual participant data, N=20.

**Table S10 | BOLD signal in the segmented hippocampus for correct and incorrect encoded associations; related to Fig. 2e.**
**S10.1**. Median BOLD signal (% signal change) extracted from individual left hippocampal ROIs during TI 1:3 stimulation for fMRI Model 2, which differentiates between correct and incorrect responses during the encode stage of the task. Statistical analyses were performed using a linear mixed effects model, with median BOLD (mdBOLD) signal as the dependent variable, independent factors for ROI (Ant - anterior, Mid – mid, Post – posterior) and response type (RspType: correct, incorrect), and random intercepts for participants (ID), N=20. **S10.2**. Same as S10.1 but for the TI 1:1 condition. Shown are the Analysis of Deviance Table (Type II Wald F tests with Kenward-Roger correction for degrees of freedom), generated by the Anova() function applied to the repeated measures analysis with mixed models fitted in R, followed by the post-hoc contrasts for models with significant interactions. [a]: Specification of the linear model fitted in the R language, Df, Degrees of freedom; Df.res, residual degrees of freedom; F, F-statistic; P, P-value; B, estimate; SE, standard error, t, t-statistic. Signif. codes: '***' 0.001, '**' 0.01, '*' 0.05, '.' 0.1.

| **S10.1 – Left Hippocampus Segmented – TI 1:3 condition** | | | | |
|---|---|---|---|---|
| **Repeated measures analysis with mixed model** | | | | |
| **lmer(mdBOLD ~ ROI*RspType + (1 \| ID)** [a] | **Df** | **Df.res** | **F** | **P** |
| ROI | 2 | 95 | 0.7125 | 0.49302 |
| RspType | 1 | 95 | 6.6161 | 0.01166 * |
| ROI: RspType | 2 | 95 | 1.4183 | 0.24722 |
| **Estimated Marginal Means** | | | | |
| **ROI** | **RspType** | **Mean** | **SE** | |
| Ant | Correct | 0.1123 | 0.0679 | |
| | Incorrect | -0.0672 | 0.0679 | |
| Mid | Correct | 0.0199 | 0.0679 | |

| | | | |
|---|---|---|---|
| | Incorrect | -0.0715 | 0.0679 |
| Post | Correct | -0.0141 | 0.0679 |
| | Incorrect | -0.0369 | 0.0679 |

| **S10.2 – Left Hippocampus Segmented – TI 1:1 condition** | | | | |
|---|---|---|---|---|
| **Repeated measures analysis with mixed model** | | | | |
| **lmer(mdBOLD ~ ROI*RspType + (1 \| ID)** [a] | **Df** | **Df.res** | **F** | **P** |
| ROI | 2 | 95 | 4.7586 | 0.01073 * |
| RspType | 1 | 95 | 0.0273 | 0.86905 |
| ROI: RspType | 2 | 95 | 0.3992 | 0.67196 |
| **Estimated Marginal Means** | | | | |

| **ROI** | **RspType** | **Mean** | **SE** |
|---|---|---|---|
| Ant | Correct | 0.2076 | 0.0553 |
| | Incorrect | 0.1653 | 0.0553 |
| Mid | Correct | 0.0921 | 0.0553 |
| | Incorrect | 0.0828 | 0.0553 |
| Post | Correct | 0.0434 | 0.0553 |
| | Incorrect | 0.0776 | 0.0553 |

**Table S11 | Correlation between evoked BOLD signal and accuracy**
**S11.1.** Correlations between the magnitude of the BOLD signal during encoding of face-name pairs and task accuracy for each stimulation condition (Sham, TI 1:1 and TI 1:3). Pearson correlations (two-tailed) were performed using the % signal change for the contrast encode > baseline, measured from regions-of-interest (ROI) in the left hippocampus (Ant - anterior, Mid – middle, Post – posterior, Hippocampus – whole hippocampus), and mean accuracy. **S11.2**. Same as S11.1 but using % signal change from the contrast correct > incorrect encodings. r, r-value; P, P-value; N=20.

| **S11.1 – Correlation analysis between BOLD signal (contrast encode > baseline) and mean accuracy** | | | |
|---|---|---|---|
| **Stimulation Type** | **ROI** | **r** | **P** |
| Sham | Ant | -0.07085 | 0.7666 |
| Sham | Mid | -0.2888 | 0.2168 |
| Sham | Post | -0.07891 | 0.7409 |
| Sham | Hippocampus | -0.01305 | 0.9565 |
| TI 1:1 | Ant | 0.07367 | 0.7576 |
| TI 1:1 | Mid | 0.01153 | 0.9615 |
| TI 1:1 | Post | 0.1273 | 0.5928 |
| TI 1:1 | Hippocampus | 0.1393 | 0.5582 |
| TI 1:3 | Ant | 0.1588 | 0.5036 |
| TI 1:3 | Mid | 0.02905 | 0.9033 |
| TI 1:3 | Post | 0.2996 | 0.1994 |
| TI 1:3 | Hippocampus | 0.22 | 0.3512 |
| **S11.2 – Correlation analysis between BOLD signal (contrast correct > incorrect encoding) and mean accuracy** | | | |
| **Stimulation Type** | **ROI** | **r** | **P** |

| | | | |
|---|---|---|---|
| Sham | Ant | 0.176 | 0.458 |
| Sham | Mid | 0.3223 | 0.1658 |
| Sham | Post | 0.1662 | 0.4837 |
| Sham | Hippocampus | 0.3292 | 0.1564 |
| TI 1:1 | Ant | 0.00597 | 0.9801 |
| TI 1:1 | Mid | -0.03798 | 0.8737 |
| TI 1:1 | Post | -0.01115 | 0.9628 |
| TI 1:1 | Hippocampus | -0.0429 | 0.8575 |
| TI 1:3 | Ant | 0.2182 | 0.3554 |
| TI 1:3 | Mid | 0.1531 | 0.5193 |
| TI 1:3 | Post | 0.1738 | 0.4636 |
| TI 1:3 | Hippocampus | 0.2247 | 0.3408 |

**Table S12 | BOLD signal in cortical regions across stimulation conditions; related to Fig. 3h.**
**S12.1**. BOLD signal (% signal change) extracted from individual ROIs underneath and between the left hemisphere stimulation electrodes, for the three stimulation conditions: sham, TI 1:1 and TI 1:3. Statistical analyses were performed using a linear mixed effects model, with median BOLD (mdBOLD) signal as the dependent variable, independent factors for stimulation type (ST: sham, TI 1:1, TI 1:3), ROI (Ant - anterior, Mid – mid, Post – posterior), and task stage (TS: encode, recall), and random intercepts for participants (ID), N=16. **S12.2.** Same as 12.1, but for the ROIs underneath the stimulation electrodes in the right hemisphere (see **Fig. S5**). **S12.3.** As per 12.1, but for the left temporal lobe (excluding the hippocampus); N=20**.** Shown are the Analysis of Deviance Table (Type II Wald F tests with Kenward-Roger correction for degrees of freedom), generated by the Anova() function applied to the repeated measures analysis with mixed models fitted in R, followed by the post-hoc contrasts for models with significant interactions using the Tukey HSD test, two-sided. [a]: Specification of the linear model fitted in the R language, Df, Degrees of freedom; Df.res, residual degrees of freedom; F, F-statistic; P, P-value; B, estimate; SE, standard error, t, t-statistic. Signif. codes: '***' 0.001, '**' 0.01, '*' 0.05, '.' 0.1.

| **S12.1 – Cortical ROIs Left Hemisphere** | | | | |
|---|---|---|---|---|
| **Repeated measures analysis with mixed model** | | | | |
| **lmer(mdBOLD ~ ST*ROI*TS + (1 \| ID)** [a] | **Df** | **Df.res** | **F** | **P** |
| Stimulation Type (ST) | 2 | 255 | 0.8285 | 0.4379 |
| ROI | 2 | 255 | 21.3390 | $2.699 \times 10^{-9}$ *** |
| Task Stage (TS) | 1 | 255 | 10.1473 | 0.0016 ** |
| Stimulation Type:ROI | 4 | 255 | 0.1870 | 0.9450 |
| Stimulation Type:Task Stage | 2 | 255 | 0.3121 | 0.7322 |
| ROI:Task Stage | 2 | 255 | 0.1377 | 0.8714 |
| Stimulation Type:ROI:Task Stage | 4 | 255 | 0.1343 | 0.9696 |
| **S12.2 – Cortical ROIs Right Hemisphere** | | | | |
| **Repeated measures analysis with mixed model** | | | | |
| **lmer(mdBOLD ~ ST*ROI*TS + (1 \| ID)** [a] | **Df** | **Df.res** | **F** | **P** |
| Stimulation Type (ST) | 2 | 165 | 0.6016 | 0.5491 |

| | | | | |
|---|---|---|---|---|
| ROI | 1 | 165 | 12.7489 | 4.669 x$10^{-4}$ *** |
| Task Stage (TS) | 1 | 165 | 15.7893 | 1.056 x$10^{-4}$ *** |
| Stimulation Type:ROI | 2 | 165 | 0.0471 | 0.9540 |
| Stimulation Type:Task Stage | 2 | 165 | 0.0776 | 0.9254 |
| ROI:Task Stage | 1 | 165 | 11.8425 | 7.335 x$10^{-4}$ *** |
| Stimulation Type:ROI:Task Stage | 2 | 165 | 0.1108 | 0.8952 |
| **S12.3 – Temporal Lobe** | | | | |
| **Repeated measures analysis with mixed model** | | | | |
| **lmer(mdBOLD ~ ST*TS + (1 \| ID)** [a] | **Df** | **Df.res** | **F** | **P** |
| Stimulation Type (ST) | 2 | 95 | 0.9704 | 0.3827 |
| Task Stage (TS) | 1 | 95 | 19.8443 | 2.294x$10^{-5}$ *** |
| Stimulation Type:Task Stage | 4 | 95 | 1.4511 | 0.2394 |

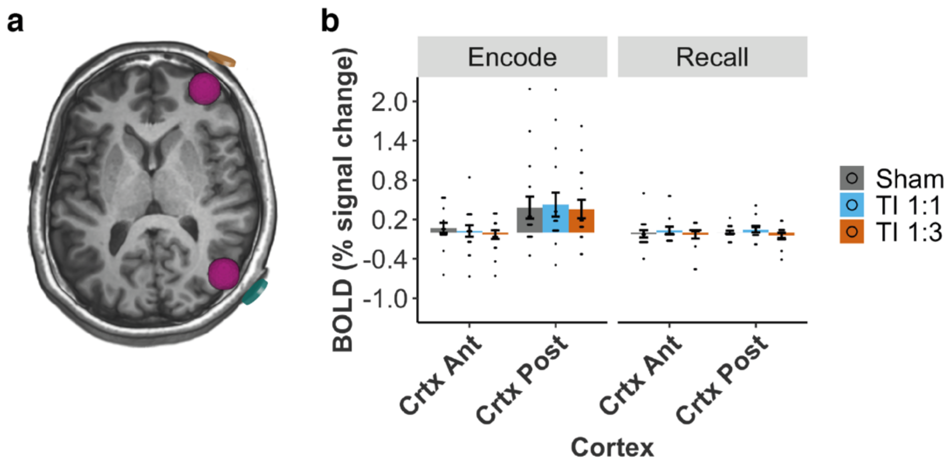


**Fig. S5: Comparison of BOLD signal in the right hemisphere electrodes**
**a**, Location of the stimulation electrodes on the right hemisphere (represented in orange and green) and the regions-of-interest (ROIs) underneath the electrodes (in purple) for an example participant. **b**, Comparison of group median percentage change in BOLD signal between stimulation conditions, in the anterior (Ant) and posterior (Post) regions of the overlying cortex, see **a** for ROI location; see **Table S12** for full statistics. Showing no difference in the BOLD signal change between stimulation conditions. Bar plots show mean and standard error (SE), black dots show individual participant data, N=16.

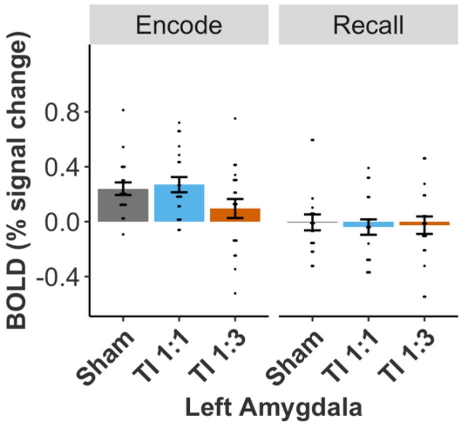

**Fig. S6: BOLD signal is not modulated by stimulation in the left amygdala.**
Group BOLD signal (% signal change) during encode and recall stages of the task across stimulation conditions (Sham, TI 1:1, TI 1:3). Statistical analyses were performed using a linear mixed effects model, with median BOLD signal as the dependent variable, independent factors for stimulation type (sham, TI 1:1, TI 1:3) and task stage (encode, recall), and random intercepts for participants, N=20. There is a main effect of task stage ($F_{(1,95)}$= 39.737, p= 9.08e$^{-9}$), but no effect of stimulation type ($F_{(2,95)}$= 1.646, p= 0.198) and no interaction between task stage and stimulation type ($F_{(2,95)}$= 1.772, p= 0.176). Bar plots show mean and standard error (SE), black dots show individual participant data, N=20.

**Table S13 | One sample t-tests for cortical regions and left amygdala during sham stimulation.**
**S13.1.** One sample t-tests on the median BOLD signal (% signal change) extracted from individual cortical masks (see **Fig. 1d** for schematics of the location of the ROIs) during the encoding stage of the task, in the absence of stimulation, i.e. sham, condition. Statistical analyses were performed using one-sample t-tests, one-sided ("greater" than zero). P-values corrected using False Discover Rate (FDR). Shown are the estimate; t, t-statistic; P, P-value; P(FDR), P-value corrected using FDR; N=16. **S13.2**. Same as S13.1 but for the ROIs underneath the stimulation electrodes in the right hemisphere (**Fig. S5**), N=16; **S13.3**. Same as S13.1 but for the left amygdala; N=20. Signif. codes: '***' 0.001, '**' 0.01, '*' 0.05, '.' 0.1.

| **Cortical ROIs Left Hemisphere** | | | |
|---|---|---|---|
| **S13.1 - One-sample t-test ("greater")** | | | |
| **ROI** | **t** | **P** | **P (FDR)** |
| Crtx Ant | -0.8763 | 0.8027 | 0.8027 |
| Crtx Mid | 2.054 | 0.0289 * | 0.0434 * |
| Crtx Post | 2.565 | 0.0108 * | 0.0323 * |
| **Cortical ROIs Right Hemisphere** | | | |
| **S13.2 - One-sample t-test ("greater")** | | | |
| **ROI** | **t** | **P** | **P (FDR)** |
| Crtx Ant | 0.9190 | 0.1863 | 0.1863 |
| Crtx Post | 2.2752 | 0.0190 * | 0.038 * |
| **Left Amygdala** | | | |
| **S13.3 - One-sample t-test ("greater")** | | | |
| **ROI** | **t** | **P** | **P (FDR)** |
| Left amygdala | 5.2502 | $2.280 \times 10^{-5}$ *** | $2.280 \times 10^{-5}$ *** |

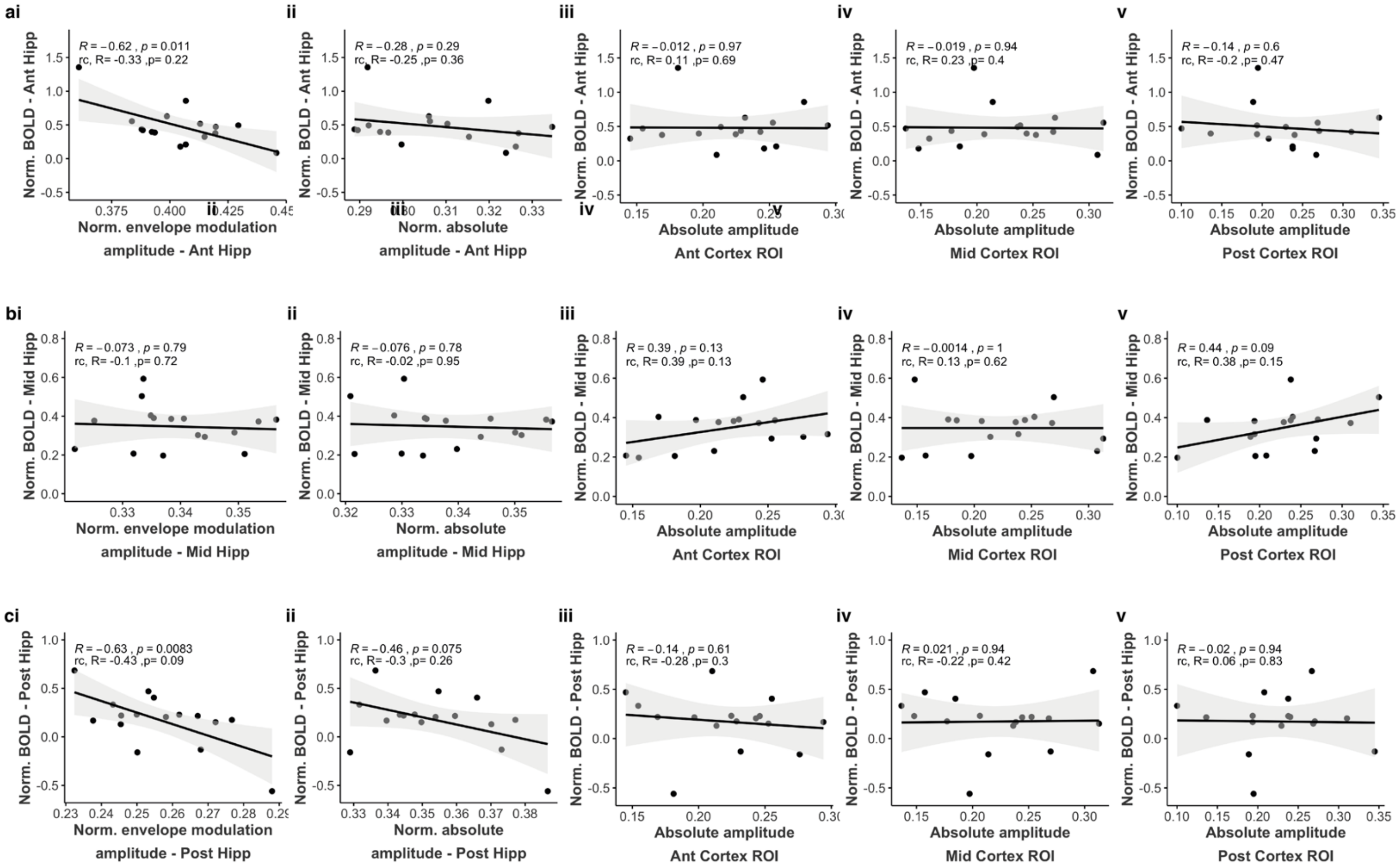


**Fig. S7:** Correlation between participants' evoked BOLD signal and their electric fields amplitudes for the TI 1:3 stimulation.

**a,** Correlation between BOLD signal from the Ant hippocampal and (i) envelope modulation amplitude in the Ant hippocampal region (ii) absolute amplitude in the Ant hippocampal region (expressed relative to total hippocampal exposure), (iii) absolute amplitude in the Ant overlying cortical region (iv) absolute amplitude in the Mid overlying cortical region (v) absolute amplitude in the Post overlying cortical region. The amplitude of the participants' evoked BOLD signal in the Ant left hippocampus during TI 1:3 stimulation was inversely correlated with the amplitude of the induced envelope modulation in this region (i, Pearson correlation but not robust correlation), but not with the absolute field amplitude in this region (ii) nor with the absolute field amplitude in the overlying cortical regions (iii-v). **b** and **c,** Same as (a), but using BOLD signal from the Mid (b) and Post (c) hippocampal region. Similar relationship between BOLD and TI fields observed for the Post region (c) as was observed in (a).

**a-c**, Median hippocampal BOLD signal and median field amplitudes in the hippocampus were normalized to total hippocampal exposure. For each plot, values at the top show the Pearson correlation coefficient (*R*), alongside p-values (*p*), regression lines (black line), 95% confidence intervals of the correlation coefficient (shaded grey); also shown are the R and p-values calculated using robust correlation (rc). Robust correlations were computed used the percentage-bend correlation, using the *pbcor* function (bending factor = 0.2) from the WRS2 package[1], which estimates linear relationships and often provides better estimates of the true relationship between variables[2]. N=16 subjects.

**Table S14 | Functional connectivity; related to Fig. 4.**
**S14.1**. Changes in functional connectivity for the sham condition during encode and recall stages of the task. The segmented regions on the left hippocampus were used as seeds (Ant, anterior; Mid, middle and Post, posterior) and nodes corresponding to the antero-temporal (AT) and posterior-medial (PM) networks as targets (see **Fig. 4a**). Functional connectivity was estimated using gPPI on the contrast correct > incorrect associations. Statistical analyses were performed using one-sample t-test, two sided, for the two task stages (encode, recall), three seeds (Ant, Mid, Post) and nodes bellowing to the AT or PM networks. The table shows the mean estimates (represented in **Fig. 4b**). t, t-statistic; P, P-value; P(FDR), P-value corrected with False Discovery Rate (FDR). N=20, **=p < 0.05, FDR-corrected; *=p < 0.05, uncorrected. **S14.2**. Functional connectivity values corresponding to the connectivity between each seed and target for each stimulation condition during the encode stage of the task (**Fig. 4c** – showing post-hoc contrasts). Statistical analyses were performed using a linear mixed effects model, with mean connectivity (mcon) as the dependent variable, independent factors for stimulation type (ST: sham, TI 1:1, TI 1:3), seed (S: Ant, anterior; Mid, middle; Post, posterior) and network (N: AT, PM), and random intercepts for participants (ID), and node from the AT or PM network. N = 20. Shown are the Analysis of Deviance Table (Type II Wald F tests with Kenward-Roger correction for degrees of freedom), generated by the Anova() function applied to the repeated measures analysis with mixed models fitted in R, followed by the post-hoc contrasts to assess the significant interactions. P value adjustment for post-hoc contrasts performed using Tukey's method (for a family of 3 estimates), two-sided. [a]: Specification of the linear model fitted in the R language, Df, Degrees of freedom; Df.res, residual degrees of freedom; F, F-statistic; P: P-value; B, estimate; SE, standard error, t, t-statistic. Signif. codes: '***' 0.001, '**' 0.01, '*' 0.05, '.' 0.1.

| **S14.1 – Changes in functional connectivity for the sham condition** | | | | | | |
|---|---|---|---|---|---|---|
| **One sample t-test, two-sided** | | | | | | |
| **Task Stage** | **Seed** | **Target** | **estimate** | **t** | **P** | **P (FDR)** |
| Encode | Ant | AT | 0.1125 | 2.3221 | 0.0223 * | 0.1336 |
| Encode | Ant | PM | -0.0070 | -0.2546 | 0.7997 | 0.8724 |
| Encode | Mid | AT | 0.1445 | 3.1171 | 0.0024 * | 0.0287 * |
| Encode | Mid | PM | 0.0438 | 1.4547 | 0.1497 | 0.2756 |
| Encode | Post | AT | 0.0775 | 1.8025 | 0.0745 | 0.2756 |
| Encode | Post | PM | 0.0046 | 0.1550 | 0.8772 | 0.8772 |
| Recall | Ant | AT | 0.0482 | 1.6270 | 0.1069 | 0.2756 |
| Recall | Ant | PM | 0.0090 | 0.4087 | 0.6839 | 0.8207 |
| Recall | Mid | AT | 0.0538 | 1.4131 | 0.1608 | 0.2756 |
| Recall | Mid | PM | 0.0231 | 1.5282 | 0.1305 | 0.2756 |
| Recall | Post | AT | -0.0275 | -0.9292 | 0.3551 | 0.5326 |
| Recall | Post | PM | 0.0125 | 0.7563 | 0.4517 | 0.6023 |

| **S14.2 – Changes in connectivity across stimulation conditions** | | | | |
|---|---|---|---|---|
| **Repeated measures analysis with mixed model – Encode** | | | | |
| **lmer(mcon ~ ST*S*NT + (1 \| ID) + (1 \| node) [a]** | **Df** | **Df.res** | **F** | **P** |
| Stimulation Type (ST) | 2 | 1576 | 16.4165 | $8.784 \times 10^{-8}$ *** |
| Seed (S) | 2 | 1576 | 0.5299 | 0.58879 |
| Network (NT) | 1 | 1576 | 2.5686 | 0.15304 |
| Stimulation Type:Seed | 2 | 1576 | 2.6275 | 0.03305 * |
| Stimulation Type:Network | 2 | 1576 | 2.8940 | 0.05565 |
| Seed:Network | 4 | 1576 | 2.0604 | 0.12774 |
| Stimulation Type:Seed:Network | 4 | 1576 | 2.5382 | 0.03835 * |

| Post-hoc contrasts | | | | | |
|---|---|---|---|---|---|
| | B | SE | df | t | P |
| Network = AT, seed = Ant | | | | | |
| Sham - TI 1:1 | 0.16982 | 0.0475 | 1576 | 3.577 | 0.0010 ** |
| Sham - TI 1:3 | 0.02835 | 0.0475 | 1576 | 2.437 | 0.8216 |
| TI 1:1 - TI 1:3 | -0.14146 | 0.0475 | 1576 | 2.321 | 0.0082 ** |
| Network = AT, seed = Mid | | | | | |
| Sham - TI 1:1 | 0.14638 | 0.0475 | 1576 | 3.084 | 0.0059 ** |
| Sham - TI 1:3 | 0.27135 | 0.0475 | 1576 | 5.717 | <.0001 *** |
| TI 1:1 - TI 1:3 | 0.12497 | 0.0475 | 1576 | 2.633 | 0.0232 * |
| Network = AT, seed = Post | | | | | |
| Sham - TI 1:1 | 0.10521 | 0.0475 | 1576 | 2.216 | 0.0687 |
| Sham - TI 1:3 | 0.11532 | 0.0475 | 1576 | 2.429 | 0.0404 * |
| TI 1:1 - TI 1:3 | 0.01011 | 0.0475 | 1576 | 0.213 | 0.9753 |
| Network = PM, seed = Ant | | | | | |
| Sham - TI 1:1 | 0.04541 | 0.0531 | 1576 | 0.856 | 0.6684 |
| Sham - TI 1:3 | 0.04958 | 0.0531 | 1576 | 0.934 | 0.6187 |
| TI 1:1 - TI 1:3 | 0.00417 | 0.0531 | 1576 | 0.079 | 0.9966 |
| Network = PM, seed = Mid | | | | | |
| Sham - TI 1:1 | 0.07352 | 0.0531 | 1576 | 1.385 | 0.3488 |
| Sham - TI 1:3 | 0.04898 | 0.0531 | 1576 | 0.923 | 0.6259 |
| TI 1:1 - TI 1:3 | -0.02454 | 0.0531 | 1576 | -0.462 | 0.8888 |
| Network = PM, seed = Post | | | | | |
| Sham - TI 1:1 | 0.04004 | 0.0531 | 1576 | 0.754 | 0.7310 |
| Sham - TI 1:3 | 0.06538 | 0.0531 | 1576 | 1.232 | 0.4345 |
| TI 1:1 - TI 1:3 | 0.02534 | 0.0531 | 1576 | 0.478 | 0.8819 |

**Table S15 | Functional connectivity – comparison across stimulation conditions during recall period of the face-name memory task.**

**S15.1**. Functional connectivity values corresponding to the connectivity between each seed and target for each stimulation condition during the recall stage of the task. Statistical analyses were performed using a linear mixed effects model, with mean connectivity (mcon) as the dependent variable, independent factors for stimulation type (ST: sham, TI 1:1, TI 1:3), seed (S: Ant, anterior; Mid, middle; Post, posterior) and network (N: AT, PM), and random intercepts for participants (ID), and node from the AT or PM network. N = 20. **S15.2**. Follow-up LMM using only stimulation conditions as independent variable.

Shown are the Analysis of Deviance Table (Type II Wald F tests with Kenward-Roger correction for degrees of freedom), generated by the Anova() function applied to the repeated measures analysis with mixed models fitted in R, followed by the post-hoc contrasts to assess the significant interactions. P value adjustment for post-hoc contrasts performed using Tukey's method (for a family of 3 estimates), two-sided. [a]: Specification of the linear model fitted in the R language, Df, Degrees of freedom; Df.res, residual degrees of freedom; F, F-statistic; P: P-value; B, estimate; SE, standard error, t, t-statistic. Signif. codes: '***' 0.001, '**' 0.01, '*' 0.05, '.' 0.1.

| S15.1 – Changes in connectivity across stimulation conditions | | | | |
|---|---|---|---|---|
| **Repeated measures analysis with mixed model – Recall** | | | | |
| **lmer(mcon ~ ST*S*NT + (1 \| ID) + (1 \| node) [a]** | **Df** | **Df.res** | **F** | **P** |
| Stimulation Type (ST) | 2 | 1576 | 8.3202 | 0.0002544 *** |

| | | | | |
|---|---|---|---|---|
| Seed (S) | 2 | 1576 | 1.2781 | 0.2788424 |
| Network (NT) | 1 | 7 | 0.2214 | 0.6522743 |
| Stimulation Type:Seed | 2 | 1576 | 0.7631 | 0.5492473 |
| Stimulation Type:Network | 2 | 1576 | 0.7193 | 0.4872524 |
| Seed:Network | 4 | 1576 | 0.8136 | 0.4434303 |
| Stimulation Type:Seed:Network | 4 | 1576 | 0.7121 | 0.5836283 |
| **S15.2 – Changes in connectivity across stimulation conditions** | | | | |
| **Repeated measures analysis with mixed model – Recall** | | | | |
| **lmer(mcon ~ ST + (1 \| ID) [a]** | **Df** | **Df.res** | **F** | **P** |
| Stimulation Type (ST) | 2 | 1598 | 8.2723 | 0.0002666 *** |
| **Post-hoc contrasts** | | | | |

| | **B** | **SE** | **df** | **t** | **P** |
|---|---|---|---|---|---|
| Sham - TI 1:1 | 0.05182 | 0.0145 | 1598 | 3.577 | 0.0010 ** |
| Sham - TI 1:3 | 0.00169 | 0.0145 | 1598 | 0.117 | 0.9925 |
| TI 1:1 - TI 1:3 | -0.05013 | 0.0145 | 1598 | -3.463 | 0.0016 ** |

**Table S16 | Memory performance statistics for face-name task; related to Fig. 5a.**
**S16.1**. Frequentist Analyses. Three main variables of interest were analysed to assess behavioural performance, i.e., response type – related to accuracy, reaction time for name selection and confidence level. Shown are the Analysis of Deviance Table (Type II Wald Chi-square or F tests with Kenward-Roger correction for degrees of freedom), generated by the Anova() function applied to the repeated measures analysis with mixed models fitted in R, followed by the post-hoc contrasts, two-sided. [a]: Specification of the linear model fitted in the R language, Df, Degrees of freedom; Df.res, residual degrees of freedom; F, F-statistic; P: P-value; B, estimate; SE, standard error, t, t-statistic, Chisq – Chi-square. N=21. Signif. codes: '***' 0.001, '**' 0.01, '*' 0.05, '.' 0.1. **S16.2.** Bayesian analysis. Results of Bayesian Regression Model for the effect of stimulation on accuracy (binomial distribution, i.e. correct and incorrect responses) estimated using the brms package in R. The specification of the model is shown in the R language, Est., the estimate of the mean marginal posterior distribution; Est. error, standard deviation of the estimate, CI, the 2.5% and 97.5% credible intervals centred on the mean, Post Dist. > 0, proportion of the posterior distribution greater than zero. N=21. Bayesian posterior density is plotted at the end of the table.

| **S16.1 – Frequentist Analyses<br>Accuracy** | | | |
|---|---|---|---|
| **Multinomial logistic regression** | | | |
| **nnet::multinom(formula= Response ~ ST)[a]** | **Df** | $\chi^2$ | **P** |
| Stimulation Type (ST) | 2 | 6.353 | 0.04173 * |
| **Post-hoc contrasts** | | | |

| | **B** | **SE** | **df** | **t** | **P** |
|---|---|---|---|---|---|
| Response = Target; Sham - TI 1:3 | -0.0282 | 0.0055 | 4 | -5.132 | 0.0068 * |
| Response = Foil; Sham - TI 1:3 | 0.01968 | 0.0108 | 4 | 1.827 | 0.1418 |
| Response = Distractor; Sham - TI 1:3 | 0.00851 | 0.0087 | 4 | 0.978 | 0.3836 |

| **Binomial logistic regression** | | | |
|---|---|---|---|
| **glmer(Accuracy ~ ST + (1\| ID) + (1\|session) + (1\|block), family = binomial(link = "logit"))[a]** | **Df** | $\chi^2$ | **P** |
| Stimulation Type (ST) | 1 | 5.8567 | 0.01552 * |

| Reaction Time | | | |
|---|---|---|---|
| **Generalised mixed linear model** | | | |
| **glmer(RT ~ ST*Response + (1\|ID) + (1\|session) + (1\|block/trial), family = inverse.gaussian(link=identity),** [a] | **Df** | $\chi^2$ | **P** |
| Stimulation Type (ST) | 1 | 3.0172 | 0.08239 |
| Response (Target, Foil, Distractor) | 2 | 424.71 | < 2 x10$^{-16}$ *** |
| Stimulation Type:Response | 2 | 2.8267 | 0.24333 |
| **Generalised mixed linear model - binomial** | | | |
| **glmer(RT ~ Accuracy*Response + (1\|ID) + (1\|session) + (1\|block/trial), family = inverse.gaussian(link=identity),** [a] | **Df** | $\chi^2$ | **P** |
| Stimulation Type (ST) | 1 | 2.9928 | 0.08364 |
| Response (Correct, Incorrect) | 1 | 425.47 | < 2 x10$^{-16}$ *** |
| Stimulation Type:Accuracy | 1 | 1.4945 | 0.22151 |
| **Confidence** | | | |
| **Cumulative Link Mixed Model** | | | |
| **clmm(Confidence ~ ST*Response + (1\|ID) + (1\|block), Hess = TRUE)**[a] | **Df** | $\chi^2$ | **P** |
| Stimulation Type (ST) | 1 | 0.35 | 0.5568 |
| Response (Target, Foil, Distractor) | 2 | 2006.1 | < 2 x10$^{-16}$ *** |
| Stimulation Type:Response | 2 | 0.42 | 0.8086 |

**S16.2 – Bayesian Analysis**

**Accuracy**

| Model | Predictor | Est. | Est. Error | CI 2.5% | CI 97.5% | Post Dist. > 0 |
|---|---|---|---|---|---|---|
| brm(Accuracy ~ Stimulation + (1\|ID) + (1\|session) + (1\|block), bernoulli(link = "logit"), prior = Prior_weak2.2, warmup = 2000, iter = 10000, chains = 4,cores = 4, control = list(adapt_delta = 0.999, max_treedepth = 17), seed = 1234)<br><br>ID = participant number<br>session = session number<br>block = block number | Intercept | 0.40 | 0.75 | -1.31 | 1.82 | - |
| | TI 1:3 | 0.12 | 0.05 | 0.02 | 0.22 | 99.21% |

| | |
|---|---|
| | TI 1:3 - Sham 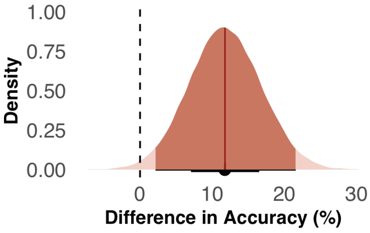  12%, 95% CI [2 22] |

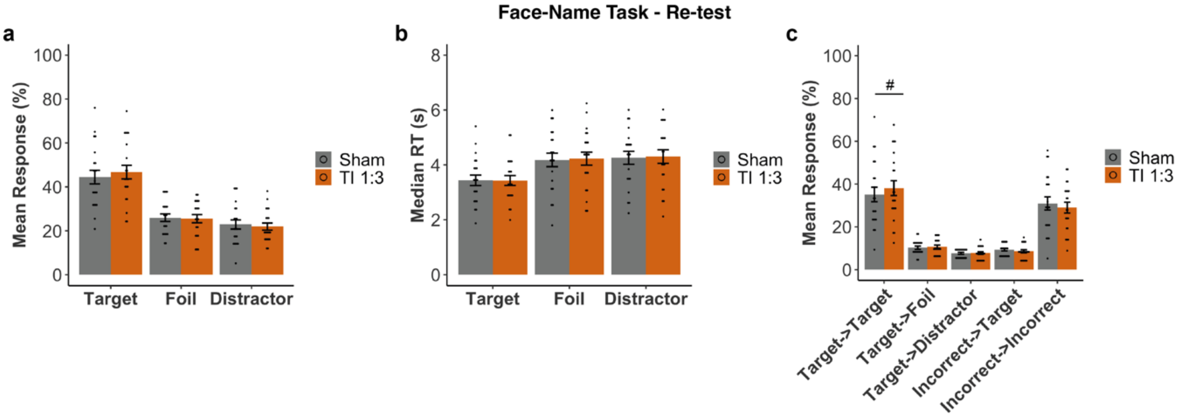


**Fig. S8**. **Behavioural performance for re-test period for sham and TI stimulation.**
**a**, Comparison of participants' memory performances during re-test between sham (grey) and TI 1:3 (orange) across response type (target, foil and distractor); there was no significant main effect of stimulation or interaction between stimulation and response type. **b**, Same as **a** but for median reaction time, again showing no significant effect of stimulation. **c**, Comparison between sham and TI 1:3 stimulation for the memory performance for each face-name pair. We assigned one of 5 categories to each face-name pair that summarise the outcome of each pair from recall to re-test: 1) Target -> Target are associations correctly identified in recall and re-test; 2,3) Target -> Foil/Distractor are associations correctly identified during recall, but forgotten during re-test, with participant selecting foil or distractor, respectively; 4) Incorrect -> Target are associations incorrectly remembered at recall, but correctly matched at re-test; 5) Incorrect -> Incorrect are associations incorrectly remembered at both recall and re-test. Cardinal (#) indicates significant exploratory post-hoc for Target ->Target condition, trend level main effect of stimulation. See **Table S18** for full statistics. **a-c**, Bar plots show mean and standard error (SE), black dots show individual participant data, N=21.

**Table S17 | Additional Memory Performance Statistics for Re-Test; related to Fig. S8 a-b**

**S17.1**. Frequentist Analyses. Response type, related to accuracy (**Fig. S8a**), and reaction time (**Fig. S8b**) for the re-test period for sham and TI 1:3 stimulation. Shown are the Analysis of Deviance Table (Type II Wald Chi-square or F tests with Kenward-Roger correction for degrees of freedom), generated by the Anova() function applied to the repeated measures analysis with mixed models fitted in R. [a]: Specification of the model in the R language, Df, Degrees of freedom; P, P-value; B, estimate; SE, standard error, t, t-statistic. Signif. codes: '***' 0.001, '**' 0.01, '*' 0.05, '.' 0.1. **S17.2.** Bayesian analysis. Results of Bayesian Regression

Model for the effect of stimulation on accuracy (binomial distribution, i.e. correct and incorrect responses) estimated using the brms package in R. The specification of the model is shown in the R language, Est., the estimate of the mean marginal posterior distribution; Est. error, standard deviation of the estimate, CI, the 2.5% and 97.5% credible intervals centred on the mean, Post Dist. > 0, proportion of the posterior distribution greater than zero. Bayesian posterior density is plotted at the end of the table.

| **S17.1 – Frequentist Analyses** | | | |
|---|---|---|---|
| **Accuracy** | | | |
| **Multinomial logistic regression** | | | |
| **nnet::multinom(formula= Response ~ ST)[a]** | **Df** | $\chi^2$ | **P** |
| Stimulation Type (ST) | 2 | 3.0076 | 0.2223 |
| **Binomial logistic regression** | | | |
| **glmer(Accuracy ~ ST + (1\| ID) + (1\|session) + (1\|block), family = binomial(link = "logit"))[a]** | **Df** | $\chi^2$ | **P** |
| Stimulation Type (ST) | 1 | 1.8392 | 0.1751 |
| **Reaction Time** | | | |
| **Generalised mixed linear model** | | | |
| **glmer(RT ~ ST*Response + (1\|ID) + (1\|session) + (1\|block/trial), family = inverse.gaussian(link=identity), [a]** | **Df** | $\chi^2$ | **P** |
| Stimulation Type (ST) | 1 | 0.2437 | 0.6215 |
| Response (Target, Foil, Distractor) | 2 | 291.30 | $< 2 \times 10^{-16}$ *** |
| Stimulation Type:Response | 2 | 0.3385 | 0.8443 |
| **Generalised mixed linear model - binomial** | | | |
| **glmer(RT ~ Accuracy*Response + (1\|ID) + (1\|session) + (1\|block/trial), family = inverse.gaussian(link=identity), [a]** | **Df** | $\chi^2$ | **P** |
| Stimulation Type (ST) | 1 | 0.2549 | 0.6137 |
| Response (Correct, Incorrect) | 1 | 290.58 | $< 2 \times 10^{-16}$ *** |
| Stimulation Type:Accuracy | 1 | 0.3373 | 0.5614 |

| **S17.2 – Bayesian Analysis** | | | | | | |
|---|---|---|---|---|---|---|
| **Accuracy** | | | | | | |
| **Model** | **Predictor** | **Est.** | **Est. Error** | **CI 2.5%** | **CI 97.5%** | **Post Dist. > 0** |
| brm(Accuracy ~ Stimulation + (1\| ID) + (1\|session) + (1\|block), bernoulli(link = "logit"), prior = Prior_weak2.2, warmup = 2000, iter = 10000, chains = 4,cores = 4, control = list(adapt_delta = 0.999, max_treedepth = 17), seed = 1234)<br><br>ID = participant number | Intercept | -0.08 | 0.62 | -1.42 | 1.33 | - |
| | TI 1:3 | 0.06 | 0.05 | -0.03 | 0.16 | 90.90% |

| | | | | | | |
|---|---|---|---|---|---|---|
| session = session number<br>block = block number | | | | | | |
| | 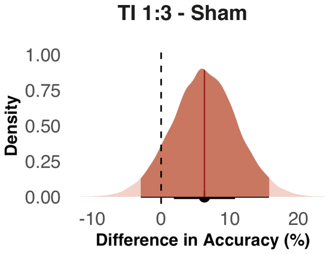<br>6%, 95% CI [-3 16] | | | | | |



**Table S18 | Memory Performance During Re-Test – Face-Name Pairs Outcome Between Recall and Re-Test; related to Fig. S8c**

Comparison between sham and TI 1:3 stimulation for the memory performance for each face-name pair. We assigned one of 5 categories to each face-name pair that summarise the outcome of each pair from recall to re-test: 1) Target -> Target are associations correctly identified in recall and re-test; 2,3) Target -> Foil/Distractor are associations correctly identified during recall, but forgotten during re-test, with participant selecting foil or distractor, respectively; 4) Incorrect -> Target are associations incorrectly remembered at recall, but correctly matched at re-test; 5) Incorrect -> Incorrect are associations incorrectly remembered at both recall and re-test. Statistical analyses were performed using a multinomial logistic regression, with the 5 categories described above and independent factors for stimulation condition (sham, TI 1:3), N=21. Shown are the Analysis of Deviance Table (Type II Wald Chi-square or F tests with Kenward-Roger correction for degrees of freedom), generated by the Anova() function applied to the repeated measures analysis with mixed models fitted in R, followed by the post-hoc contrasts, two-sided. [a]: Specification of the model in the R language, Df, Degrees of freedom; P, P-value; B, estimate; SE, standard error, t, t-statistic. Signif. codes: '***' 0.001, '**' 0.01, '*' 0.05, '.' 0.1.

| **Multinomial logistic regression** | | | | | |
|---|---|---|---|---|---|
| **nnet::multinom(formula = Response Category ~ Stimulation Type)** | **Chisq** | **Df** | | **P** | |
| Stimulation Type | 9.0111 | 4 | | 0.06082 . | |
| **Post-hoc contrasts** | | | | | |
| | **B** | **SE** | **df** | **t** | **P** |
| Target -> Target | | | | | |
| Sham - TI 1:3 | -0.028136 | 0.0104 | 8 | -2.711 | 0.0266 |
| Target -> Foil | | | | | |
| Sham - TI 1:3 | -0.003993 | 0.0082 | 8 | -0.485 | 0.6407 |
| Target -> Distractor | | | | | |
| Sham - TI 1:3 | -0.000566 | 0.0054 | 8 | -0.105 | 0.9187 |
| Incorrect -> Target | | | | | |
| Sham - TI 1:3 | 0.008824 | 0.0072 | 8 | 1.232 | 0.2530 |
| Incorrect -> Incorrect | | | | | |
| Sham - TI 1:3 | 0.023871 | 0.0198 | 8 | 1.207 | 0.2619 |

**Table S19 | Summary of post-stimulation side effects questionnaire for fMRI experiment**

Summary statistics for adverse effects questionnaire (adapted from [3]). Subjects rated each item between 1 (absent) and 4 (severe). Average Intensity and Range calculated over the whole cohort of participants.

| | Side effects questionnaire | | |
|---|---|---|---|
| | Average Intensity (SD) | Number of incidences > 1 (out of 21*) | Range |
| **Headache** | 1.75(0.5) | 4 | 1-2 |
| **Scalp pain** | 1.67(0.5) | 4 | 1-2 |
| **Burning** | 2(0) | 4 | 2 |
| **Warmth** | 2(0) | 4 | 2 |
| **Tingling** | 2.29(0.5) | 8 | 2-3 |
| **Prickling** | 2(0) | 3 | 2 |
| **Itching** | 1.67(0.5) | 4 | 1-2 |
| **Metallic taste** | 1(0) | 1 | 1 |
| **Sleepiness/Fatigue** | 2.57(0.9) | 8 | 1-4 |
| **Trouble concentrating** | 2.17(0.4) | 7 | 2-3 |
| **Effect on performance** | 2(0.6) | 6 | 1-3 |
| **Acute mood change** | 1(0) | 1 | 1 |
| **Other** | 1(0) | 1 | 1 |

* Initial sample size was 21. One participant was excluded from imaging analysis because of excessive movement. Information for all participants is included in the table. SD, standard deviation.

**Table S20 | Summary of post-stimulation side effects questionnaire for TI and Sham sessions for behavioural experiment**

Summary statistics for adverse effects questionnaire (adapted from [3]). Subjects rated each item between 1 (absent) and 4 (severe). Average Intensity and Range calculated over the whole cohort of participants. Statistical analyses were performed Wilcoxon signed-rank tests, two-sided.

| | TI session | | | Sham session | | | Wilcoxon signed-rank tests | |
|---|---|---|---|---|---|---|---|---|
| | Average Intensity (SD) | Number of incidences > 1 (out of 21) | Range | Average Intensity (SD) | Number of incidences > 1 (out of 21) | Range | Z | p |
| **Headache** | 1.2(0.5) | 3 | 1-3 | 1(0.2) | 1 | 1-2 | -1.063 | 0.288 |

| | | | | | | | | |
|---|---|---|---|---|---|---|---|---|
| **Scalp pain** | 1.1(0.3) | 2 | 1-2 | 1.1(0.2) | 3 | 1-2 | 0.471 | 0.638 |
| **Burning** | 1.1(0.2) | 1 | 1-2 | 1(0) | 0 | 1 | -1 | 0.317 |
| **Warmth/Heat** | 1.1(0.3) | 2 | 1-2 | 1(0.2) | 1 | 1-2 | -0.592 | 0.554 |
| **Tingling** | 1.5(0.6) | 8 | 1-3 | 1.4(0.6) | 9 | 1-3 | 0 | 1 |
| **Itching** | 1.2(0.4) | 5 | 1-2 | 1(0) | 0 | 1 | -2.354 | 0.019* |
| **Metallic taste** | 1(0) | 0 | 1 | 1(0) | 0 | 1 | - | - |
| **Fatigue** | 1.6(0.7) | 10 | 1-3 | 1.6(0.8) | 9 | 1-3 | -0.112 | 0.911 |
| **Sleepiness** | 1.7(0.9) | 9 | 1-3 | 1.6(0.8) | 8 | 1-3 | -0.473 | 0.637 |
| **Acute mood change** | 1(0) | 0 | 1 | 1(0) | 0 | 1 | - | - |
| **Visual Sensation** | 1(0) | 0 | 1 | 1(0) | 0 | 1 | - | - |
| **Dizziness/ Nausea** | 1(0.2) | 1 | 1-2 | 1(0) | 0 | 1-2 | -1 | 0.317 |
| **Nervousness/ Anxiety** | 1(0.2) | 1 | 1-2 | 1(0) | 0 | 1-2 | -1 | 0.317 |
| **Discomfort/ Unpleasant** | 1.1(0.3) | 2 | 1-2 | 1(0) | 0 | 1-3 | -1.432 | 0.152 |
| **Other** | 1(0) | 0 | 1 | 1(0) | 0 | 1 | - | - |

**Table S21 | Perceptual sensations and threshold across participants (ID) for conventional transcranial alternating current stimulation (tACS) and TI stimulation for fMRI experiment**

Perceptual sensations reported by participants and thresholds (i.e. current intensity for which a perceptual sensation was first reported). Participants were exposed to short conventional tACS and TI stimulation during setup, immediately before entering the MRI scanner. TACS stimulation was always administered first, so participants would be aware of the possible sensations elicited by electrical stimulation. Current intensity was ramped in steps of 0.1 mA until a sensation was reported, starting from electrode pair *e*1-*e*2 and then moving to *e*3-*e*4.

| | **tACS (5 Hz)** | | | | **TI (CF = 2 and 2.005 kHz, Δ*f* = 5 Hz)** | | | |
|---|---|---|---|---|---|---|---|---|
| | ***e*1 – *e*2** | | ***e*3 - *e*4** | | ***e*1 – *e*2** | | ***e*3 – *e*4** | |
| **ID** | **Threshold (mA)** | **Sensation** | **Threshold (mA)** | **Sensation** | **Threshold (mA)** | **Sensation** | **Threshold (mA)** | **Sensation** |
| **1** | 0.3 | burning | 0.4 | light burning | none | none | 3 | stinging |
| **2** | 0.5 | prickling | 0.4 | stinging | none | none | none | none |
| **3** | 0.3 | tingling | 0.4 | tingling | none | none | none | none |
| **4*** | 0.3 | warmth | 0.5 | vibrating | none | none | none | none |
| **5** | 1 | vibration | 1.2 | tingling | none | none | none | none |
| **6** | 0.3 | prickling | 0.3 | tingling | none | none | none | none |
| **7** | 0.5 | prickling | 0.7 | itchy | none | none | none | none |
| **8** | 0.7 | prickling | 0.7 | warmth | none | none | none | none |
| **9** | 0.7 | tingling | 0.7 | tingling | none | none | none | none |
| **10** | 0.3 | prickling | 1.2 | tingling | none | none | none | none |
| **11** | 0.5 | prickling | 0.5 | warmth | none | none | none | none |

| | | | | | | | | |
|---|---|---|---|---|---|---|---|---|
| **12** | 0.3 | prickling | 1.2 | tingling | none | none | none | none |
| **13** | 0.5 | stinging | 0.5 | tingling | none | none | none | none |
| **14** | 0.3 | tingling | 0.5 | tingling | none | none | none | none |
| **15** | 0.3 | tingling | 0.5 | tingling | 2 | tingling | 3 | tingling |
| **16** | 0.5 | prickling | 0.5 | prickling | none | none | none | none |
| **18** | 0.5 | tingling | 0.7 | tingling | none | none | none | none |
| **19** | 0.3 | tingling | 0.7 | tingling + warmth | none | none | 3 | pressure # |
| **20** | 0.3 | tingling | 0.5 | warmth | none | none | 3 | burning # |
| **21** | 0.5 | tingling | 0.5 | tingling + warmth | none | none | 3 | tingling # |
| **22** | 0.5 | stinging | 0.5 | stinging | none | none | none | none |

tACS – transcranial alternating current stimulation; TI – temporal interference stimulation; CF – carrier frequency; Δ*f* – delta frequency or modulated frequency; e – electrode. * Participant was excluded from imaging analysis because of excessive movement; # No sensations reported in the scanner.

**Table S22 | Perceptual sensations and threshold across participants (ID) for conventional transcranial alternating current stimulation (tACS) and TI stimulation – Session 1 behavioural experiment**

Perceptual sensations reported by participants and thresholds (i.e. current intensity for which a perceptual sensation was first reported). Participants were exposed to short conventional tACS and TI stimulation during setup, immediately before entering the MRI scanner. TACS stimulation was always administered first, so participants would be aware of the possible sensations elicited by electrical stimulation. Current intensity was ramped in steps of 0.1 mA until a sensation was reported, starting from electrode pair *e*1-*e*2 and then moving to *e*3-*e*4.

| | **tACS (5 Hz)** | | | | **TI (CF = 2 and 2.005 kHz, Δ*f* = 5 Hz)** | | | |
|---|---|---|---|---|---|---|---|---|
| | ***e*1 – *e*2** | | ***e*3 - *e*4** | | ***e*1 – *e*2** | | ***e*3 – *e*4** | |
| **ID** | **Threshold (mA)** | **Sensation** | **Threshold (mA)** | **Sensation** | **Threshold (mA)** | **Sensation** | **Threshold (mA)** | **Sensation** |
| **1** | 0.3 | pinprick | 0.3 | pinprick | 2 | pinprick | 2 | pinprick |
| **2** | 0.1 | stinging | 0.1 | stinging | 1.5 | stinging | 2 | stinging |
| **3** | 0.3 | pinprick | 0.3 | pinprick | 2 | tingling | 3 | tingling |
| **4** | 0.3 | tingling | 0.3 | tingling | 1.5 | vibrations | 1.5 | vibrations |
| **5** | 0.3 | tingling | 0.3 | tingling | none | none | none | none |
| **6** | 0.3 | pinprick | 0.7 | tingling | 1 | numbing | 2 | tingling |
| **7** | 0.5 | tingling | 0.3 | tingling | none | none | 2.5 | pressure |
| **8** | 0.5 | pinprick | 0.5 | pinprick | none | none | none | none |
| **9** | 0.3 | stinging | 0.5 | stinging | 2 | vibrations | 2 | vibrations |
| **10** | 0.1 | pinprick | 0.3 | pinprick | none | none | none | none |
| **11** | 0.1 | tingling | 0.3 | tingling | none | none | none | none |
| **12** | 0.5 | tingling | 0.5 | tingling | none | none | none | none |
| **13** | 0.7 | phosphenes | 0.5 | pulling | 1 | push/pull | 1 | push/pull |
| **14** | 0.3 | pinprick | 0.1 | pinprick | 1 | white noise | 1 | white noise |
| **15** | 0.3 | pinprick | 0.1 | heat | 2 | pinprick | 2 | pinprick |
| **16** | 0.1 | tingling | 0.3 | tingling | 2 | tingling | 2.5 | tingling |
| **17** | 0.3 | pinching | 0.3 | pinprick | none | none | 2 | hair prickle |

| | | | | | | | | |
|---|---|---|---|---|---|---|---|---|
| **18** | 0.3 | pinprick | 0.3 | pinprick | none | none | none | none |
| **19** | 0.3 | pinprick | 0.5 | pinprick | 2 | tingling | none | none |
| **20** | 0.1 | pinprick | 0.1 | pinprick | 1.5 | pain | 1.5 | pain |
| **21** | 0.1 | pinprick | 0.5 | pinprick | 1 | laughter | 1 | laughter |

tACS – transcranial alternating current stimulation; TI – temporal interference stimulation; CF – carrier frequency; Δ*f* – delta frequency or modulated frequency; e – electrode.

**Table S23 | Perceptual sensations and threshold across participants (ID) for conventional transcranial alternating current stimulation (tACS) and TI stimulation – Session 2 behavioural experiment**

Perceptual sensations reported by participants and thresholds (i.e. current intensity for which a perceptual sensation was first reported). Participants were exposed to short conventional tACS and TI stimulation during setup, immediately before entering the MRI scanner. TACS stimulation was always administered first, so participants would be aware of the possible sensations elicited by electrical stimulation. Current intensity was ramped in steps of 0.1 mA until a sensation was reported, starting from electrode pair *e*1-*e*2 and then moving to *e*3-*e*4.

| | **tACS (5 Hz)** | | | | **TI (CF = 2 and 2.005 kHz, Δ*f* = 5 Hz)** | | | |
|---|---|---|---|---|---|---|---|---|
| | ***e*1 – *e*2** | | ***e*3 - *e*4** | | ***e*1 – *e*2** | | ***e*3 – *e*4** | |
| | **Threshold (mA)** | **Sensation** | **Threshold (mA)** | **Sensation** | **Threshold (mA)** | **Sensation** | **Threshold (mA)** | **Sensation** |
| **1** | 0.3 | tingling | 0.3 | tingling | 1.5 | vibrations | 2 | pinprick |
| **2** | 0.1 | tingling | 0.1 | tingling | 1.5 | stinging | 2 | stinging |
| **3** | 0.1 | pinprick | 0.3 | tingling | 2 | tickling | 3 | tingling |
| **4** | 0.3 | tingling | 0.3 | tingling | 1.5 | tingling | 1.5 | vibrations |
| **5** | 0.3 | tingling | *RM* | *RM* | 2 | twitching | none | none |
| **6** | 0.3 | tingling | 0.3 | tingling | 2 | tingling | 2 | tingling |
| **7** | 0.5 | tingling | 0.3 | tingling | none | none | 2.5 | pressure |
| **8** | 0.3 | pinprick | 0.3 | pinprick | none | none | none | none |
| **9** | 0.3 | tingling | 0.3 | tingling | none | none | 2 | vibrations |
| **10** | 0.1 | pinprick | 0.5 | pinprick | 2 | none | none | none |
| **11** | 0.1 | tingling | 0.3 | tingling | none | none | none | none |
| **12** | 0.3 | tingling | 0.3 | mild tingling | 2 | none | none | none |
| **13** | 0.7 | piercing | 0.9 | none | 2 | slight pulling in occipital region | 1 | push/pull |
| **14** | 0.1 | pinprick | 0.5 | faster pinpricks | 1 | white noise | 1 | white noise |
| **15** | 0.1 | pinprick | 0.3 | pinprick | 1.5 | pinprick | 2 | pinprick |
| **16** | 0.3 | touch | 0.7 | sting | 2 | tingling | 2.5 | tingling |
| **17** | 0.5 | pinprick/small needle | 0.5 | pinprick/ small needle | 1 | light tapping | 2 | hair prickle |
| **18** | 0.1 | tingling | 0.1 | tingling | none | none | none | none |
| **19** | 0.3 | tingling | 0.5 | slight tingling | 1 | hair moving | none | none |
| **20** | 0.1 | tingling | 0.1 | tingling | 1.5 | itchy | 1.5 | pain |
| **21** | 0.1 | pinprick | 0.5 | pinprick | 1.5 | headache | 1.5 | headache |

tACS – transcranial alternating current stimulation; TI – temporal interference stimulation; CF – carrier frequency; Δ*f* – delta frequency or modulated frequency; e – electrode. RM – record missing

**Table S24 | Blinding effectiveness**

During the behavioural sessions participants were asked at 4 time points whether they thought they had stimulation and how confident they were. The two questions were combined into a weighted score (WS), whereby a "yes" answer was assigned a +1 value and "no" answer a value of -1, which were then multiplied by the confidence rating. Shown is the statistical model applied to investigate whether there was an effect of stimulation (ST: sham, TI 1:3) or time point (Q) in the weighted score for perceiving stimulation, the Analysis of Deviance Table (Type II Wald chisquare tests), generated by the Anova() function applied to the model fitted in R. [a]: Specification of the model fitted in the R language, Df, Degrees of freedom; $\chi^2$, Chi-square; P, P-value; N=21 (ID). Signif. codes: '***' 0.001, '**' 0.01, '*' 0.05, '.' 0.1.

| **Repeated measures analysis with mixed model** | | | |
|---|---|---|---|
| **lmer(WS ~ ST*Q + (1\|S_ID) + (1\|Session)+ (1 \| ID)** [a] | **Df** | $\chi^2$ | **P** |
| Stimulation Type (ST) | 1 | 0.0710 | 0.7899 |
| Time point probed (Q) | 3 | 4.7361 | 0.1922 |
| Stimulation Type:Time point probed | 3 | 3.0186 | 0.3888 |

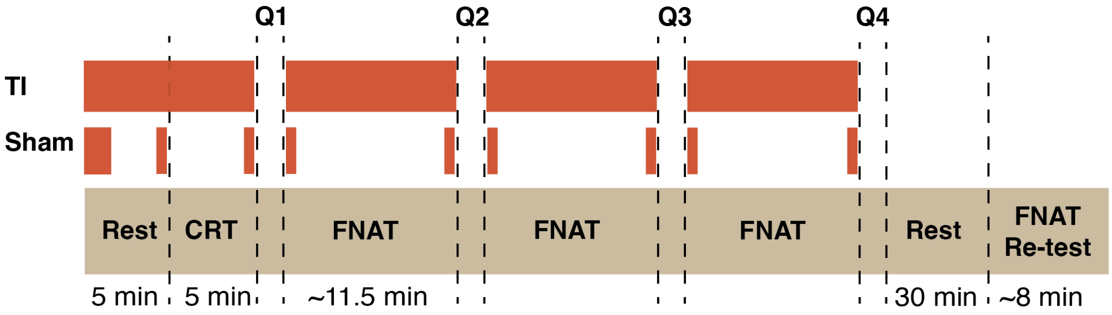


**Fig. S9. Experimental design for behavioural experiment.**

Red horizontal bars indicate stimulation periods; Q1-4, stimulation blindness questionnaire 1 to 4. FNAT, Face-Name Task; CRT, Choice-Reaction Time Task. The CRT is a simple 2-alternative force choice paradigm commonly used for testing general alertness and motor speed. Participants were presented with a central right or left pointing arrows and asked to press a right or left button matching the direction of the arrow, as described in[4]. The task consisted of 136 ± 1 trials, 1 ± 0.2 s inter stimulus interval (ISI, randomly drawn for each trial from a normal distribution with a mean of 1 s and variance of 0.2 s). Accuracy and reaction times were similar between stimulation conditions. There was no difference in accuracy between conditions (LMM: $\chi^2(1) = 0.179$, p= 0.672, accuracy mean±SD, sham: 99.7±5.4%; TI: 99.6±6%) or median reaction times (LMM: $\chi^2(1)= 3.507$, p= 0.0611, reaction time for correct trials median±SD, sham 0.394± 0.073 s; TI: 0.398± 0.076 s).

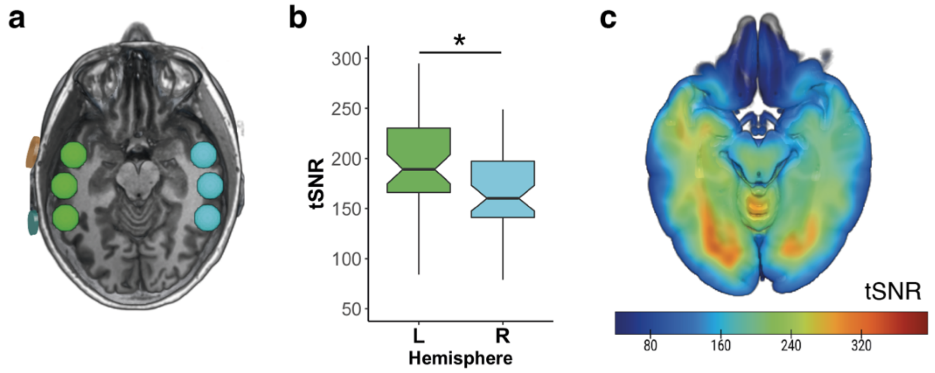


**Fig. S10: Temporal Signal to Noise Ratio (tSNR)**
**a,** To assess whether the presence of electrodes on the scalp affected the quality of the fMRI images, we estimated the temporal signal-to-noise ratio (tSNR) in the brain regions underneath and between the electrodes on the left hemisphere (green) and their contralateral equivalents (cyan, right hemisphere ROIs). The location of the stimulation electrodes on the left hemisphere are represented in orange for an example participant. **b,** Group tSNR for the left (L) and right (R) hemisphere ROIs (Median BOLD signal; centre line, median; box limits, upper and lower quartiles; lines, 1.5 x interquartile range; $^{*}p<0.05$). tSNR was calculated by dividing the mean of the signal over time by the standard deviation over the whole fMRI acquisition for the face-name task (N=16 where electrodes were clearly visible on T1 images). A linear mixed model (LMM) with tSNR as the dependent variable and hemisphere as the independent variable, and random intercepts for participants and ROI indicated a main effect of hemisphere ($F_{(1,77)}= 13.528$, $p= 4.331x10^{-4}$) explained by higher tSNR in the left compared to the right. This indicates that there was no reduction in tSNR underneath and between the electrodes in the left hemisphere, and in fact tSNR was higher in the left compared to the right hemisphere. **c**, Mean tSNR map of the fMRI task time course data calculated for each voxel and averaged across all participants (N=20). The tSNR map is overlaid on the MNI standard brain and shown in neurological orientation. Showing a slice in the same orientation as panel **a**, and confirming higher tSNR in this slice for left compared to right hemisphere. Hemispheric differences in tSNR in the regions shown are likely explained by differences in fMRI BOLD signal during the task. These results indicate that our electrodes and stimulation equipment did not introduce the patterns of noise in the MR signal identified in some studies conducting simultaneous brain stimulation and fMRI, which are typically characterised by a reduction of tSNR underneath the stimulation electrodes [5]. The mean tSNR image [1] and raw fMRI images of the first 200 TRs (400 seconds) of each participant [2] are available in the online repository.
[1] - https://gitlab.surrey.ac.uk/nemo/ti-paper/-/tree/main/Data/MRI/FaceNameTask/tSNR
[2] - https://gitlab.surrey.ac.uk/nemo/ti-paper/-/tree/main/Data/MRI/FaceNameTask/Raw_fMRI_gifs

**Table S25 | ContES Checklist**
Summary of technological, safety and noise tests, and methodological factors for concurrent transcranial electrical stimulation (tES)-fMRI studies based on the ContES Checklist[6]

**ContES Checklist**

| **Technological factors** | |
|---|---|
| Manufacturer of Stimulator | Custom-made device described in Grossman N. et al., Cell, 2017 |
| MR Conditional Electrodes | Self-adhesive TENS, 1.5 cm x 1.5 cm with the |

| | |
|---|---|
| | corners cut to produce a rounded shape |
| Electrode Positioning | Electrode 1 (*e*1) and electrode 3 (*e*3) were positioned on the left hemisphere at the level of the nasion plane, *e*1 was positioned anterior to *e*3 (*e*1 at 50% of the subject's half circumference minus 2.5 cm and *e*3 at 50% of the subject's half circumference plus 2.5 cm, both counting from the nasion; such that the centres of the electrodes were 5 cm apart). Electrodes 2 and 4 (*e*2 and *e*4) were positioned on the right hemisphere at a plane just above the eyebrow, *e*2 was positioned anterior to *e*4 (*e*2 at 20% of the subject's half circumference minus 1 cm and *e*4 at 70% of the subject's half circumference plus 1 cm, both counting from the nasion). *e*1-*e*2 formed one electrode pair and *e*3-*e*4 the second electrode pair.<br><br>Electrodes were kept in place using medical tape (3M$^{TM}$ Micropore$^{TM}$ medical tape). |
| MR Conditional Skin-Electrode Interface | Ten20 conductive paste *(Weaver and Company, Aurora, CO, USA)* |
| Amount of Contact Medium (Paste/Gel/Electrolyte) | Around 1 mm of paste was manually placed on the electrodes |
| Electrode Placement Visualization | See Fig. 1b |
| RF Filter | NeuroConn DC-STIMULATOR MR RF filter module with MRI-compatible cables and electrodes *(neuroConn GmbH, Ilmenau, Germany)*. Each RF filter box is connected to an ethernet cable via an ethernet RF filter installed in the penetration panel. |
| Wire Routing Pattern | The ethernet cable inside the scanner room is routed to the back of the scanner bore using sandbags to avoid loops.<br>The ethernet cable is connected to a 4-channel MR RF filter box placed in the back of the head coil inside the scanner bore. The stimulation electrode leads are connected to the filter box. Leads exit the MR 32 channel head coil on each side. The MR filter box is positioned in the centre of the bore. |
| tES-fMRI Machine Synchronization/Communication | The stimulus PC controlled the stimulator via a USB cable. The start of each stimulation block was determined by a TTL pulse from the scanner delivered to the stimulus PC via an USB cable. |
| **Safety and noise tests** | |
| MR Conditionality Specifics for tES | 3T Siemens Verio, 32-channel head coil; MRI |

| | |
|---|---|
| Setting | sequence parameters are available in the "MRI data acquisition" section |
| tES-fMRI Setting Test - Safety Testing | No temperature tests were performed during the experiment.<br><br>Participants were first exposed to low frequency stimulation followed by TI stimulation, for each electrode pair at a time, first *e*1- *e*2 followed by *e*3- *e*4. Stimulation started at 0.1 mA and increased in steps of 0.1 mA until participants reported any sensations associated to stimulation (i.e., pins and needles, burning, phosphenes, etc.) or until maximum intensities for the experimental protocol were reached (2 mA for *e*1- *e*2 and 3 mA for *e*3- *e*4). Perceptual sensations and their thresholds were recorded and reported.<br><br>At the end of the MRI session participants completed a post-stimulation side-effects questionnaire. |
| tES-fMRI Setting Test - Subjective Intolerance Reporting | All participants tolerated the stimulation well. |
| tES-fMRI Setting Test - Noise/Artifact | Signal to Noise Ratio (SNR) analyses were performed on the fMRI images. No signal dropout was observed in the electrode regions. |
| Impedance Testing | Impedances were checked during setup outside the scanner and after entering the scanner. |
| **Methodological factors** | |
| Concurrent tES-fMRI Timing | See Fig. 2a |
| Imaging Session Timing | Structural sequences preceded fMRI acquisition. All sequences were performed with the stimulation electrodes placed on the subjects' head and the circuit fully connected. |
| tES Experience Report | See Table S19 |

## References

1. Mair, P. & Wilcox, R. Robust statistical methods in R using the WRS2 package. *Behav Res Methods* **52**, 464-488 (2020).

2. Rousselet, G.A. & Pernet, C.R. Improving standards in brain-behavior correlation analyses. *Front Hum Neurosci* **6**, 119 (2012).

3. Brunoni, A.R., *et al.* A systematic review on reporting and assessment of adverse effects associated with transcranial direct current stimulation. *Int J Neuropsychopharmacol* **14**, 1133-1145 (2011).

4. Violante, I.R., *et al.* Externally induced frontoparietal synchronization modulates network dynamics and enhances working memory performance. *Elife* **6** (2017).

5. Antal, A., *et al.* Imaging artifacts induced by electrical stimulation during conventional fMRI of the brain. *Neuroimage* **85 Pt 3**, 1040-1047 (2014).

6. Ekhtiari, H., *et al.* A checklist for assessing the methodological quality of concurrent tES-fMRI studies (ContES checklist): a consensus study and statement. *Nat Protoc* **17**, 596-617 (2022).
