# Supplementary material for: Non-invasive temporal interference electrical stimulation of the human hippocampus
Source: Nat Neurosci. 2023 Oct 19;26(11):1994–2004. doi: 10.1038/s41593-023-01456-8 (PMC10620081; doi:10.1038/s41593-023-01456-8)
Supplement: Supplementary file 2 — Reporting Summary [file 41593_2023_1456_MOESM2_ESM.pdf]

Corresponding author(s): Nir Grossman, Ines R. Violante

Last updated by author(s): Sep 3, 2023

Reporting Summary

Nature Portfolio wishes to improve the reproducibility of the work that we publish. This form provides structure for consistency and transparency in reporting. For further information on Nature Portfolio policies, see our Editorial Policies and the Editorial Policy Checklist.

Statistics

For all statistical analyses, confirm that the following items are present in the figure legend, table legend, main text, or Methods section.

n/a	Confirmed	
☐	☒	The exact sample size (*n*) for each experimental group/condition, given as a discrete number and unit of measurement
☐	☒	A statement on whether measurements were taken from distinct samples or whether the same sample was measured repeatedly
☐	☒	The statistical test(s) used AND whether they are one- or two-sided<br>*Only common tests should be described solely by name; describe more complex techniques in the Methods section.*
☒	☐	A description of all covariates tested
☐	☒	A description of any assumptions or corrections, such as tests of normality and adjustment for multiple comparisons
☐	☒	A full description of the statistical parameters including central tendency (e.g. means) or other basic estimates (e.g. regression coefficient) AND variation (e.g. standard deviation) or associated estimates of uncertainty (e.g. confidence intervals)
☐	☒	For null hypothesis testing, the test statistic (e.g. *F*, *t*, *r*) with confidence intervals, effect sizes, degrees of freedom and *P* value noted<br>*Give P values as exact values whenever suitable.*
☐	☒	For Bayesian analysis, information on the choice of priors and Markov chain Monte Carlo settings
☒	☐	For hierarchical and complex designs, identification of the appropriate level for tests and full reporting of outcomes
☐	☒	Estimates of effect sizes (e.g. Cohen's *d*, Pearson's *r*), indicating how they were calculated

Our web collection on statistics for biologists contains articles on many of the points above.

Software and code

Policy information about availability of computer code

Data collection	sEEG data was collected using RHS Stim/Recording Controller (Intan Technologies). Behavioural data was collected using Psychtoolbox for MATLAB Version 9.4 (R2018a), https://gitlab.eps.surrey.ac.uk/nemo/facenametask. MRI data was collected using a 3T Siemens Verio. Perceptual sensations associated with stimulation were collected electronically using Microsoft Excel or Adobe Acrobat Reader.
Data analysis	Electric field modeling was performed using the the Sim4Life platform (ZMT ZurichMedTech AG, Zurich, Switzerland). Individualised models were generated using the SimNIBS framework (version 3.2), FreeSurfer (version 6.0.0) and the iSEG software (IT'IS Foundation, Zurich, Switzerland). sEEG data were pre-processed using MATLAB Version 9.4 (R2018a) (Mathworks, Natick, MA). FMRI data were pre-processed and analysed using FMRIB Software Library (FSL version 6.0.1). Statistical analyses were conducted using R version 3.6.0 via RStudio and plots were generated with the ggplot2 package. Data and key scripts are available on Gitlab (https://gitlab.eps.surrey.ac.uk/nemo/ti-paper). The code for the face-name task is available on Gitlab (https://gitlab.eps.surrey.ac.uk/nemo/facenametask).

For manuscripts utilizing custom algorithms or software that are central to the research but not yet described in published literature, software must be made available to editors and reviewers. We strongly encourage code deposition in a community repository (e.g. GitHub). See the Nature Portfolio guidelines for submitting code & software for further information.

Data

Policy information about availability of data

All manuscripts must include a data availability statement. This statement should provide the following information, where applicable:

- Accession codes, unique identifiers, or web links for publicly available datasets
- A description of any restrictions on data availability
- For clinical datasets or third party data, please ensure that the statement adheres to our policy

Group-level data used to generate the fMRI figures are available in NeuroVault (https://neurovault.org/collections/11908/). A 3D viewer for visualisation of the spatial distribution of the amplitude modulation magnitude (TI) and of the maximum carrier frequency electric field (HF) is available online (https://osparc.io/study/9641ba42-c4db-11ed-b8b9-02420a0b5f22). Faces used in the face-name task were retrieved from the Chicago Face Database v.2.0.3.

Human research participants

Policy information about studies involving human research participants and Sex and Gender in Research.

Reporting on sex and gender	Inclusion criteria for human in-vivo experiments included male and female participants defined based on self-identified biological sex. The final sample for the fMRI experiment included 11 females and 9 males. The final sample for the behavioural experiment included 10 females and 11 males. No sex-based analyses were performed, as our sample sizes were modest to investigate sex-based effects. The sample for the cadaver experiment was composed of 1 male individual.
Population characteristics	All participants in the in-vivo experiments were educated to degree level or above with no self-reported history of neurological or psychiatric illness. For the sample included in the fMRI experiment the age range was 20 to 54 years old, mean age 27.1 ± 7.6 SD, 19 right-handed and 1 left-handed. For the behavioural study participants age range was 19 to 30 years old, mean age 22.7 ± 3.2 SD, all right-handed. For the ex-vivo experiment, we included one human male cadaver (93 years old) with no known brain disorder. No racial or ethnic information was collected.
Recruitment	Participants were recruited via posters placed in public areas (e.g. university sites), flyers, suitable websites (e.g., university websites), university mailing lists and word of mouth. Our population was recruited based on convenience sampling and is therefore biased towards university educated individuals. The human male cadaver was provided by the “service des corps donnés à la science” by Aix Marseille Université.
Ethics oversight	Data for the reference head model used in this study was previously published and collected in accordance with the appropriate ethical approval from the Institute for Biomedical Engineering at the ETH, Zurich, Switzerland. Ethical approval for the human cadaver experiments was granted by the Faculty of Medicine La Timone (Aix Marseille Université). Ethical approval for in-vivo experiments with human participants was granted by the Imperial College Research Ethics Committee (ICREC). Participants gave written informed consent and those taking part in the in-vivo experiments were compensated for their time. The study conforms to the Declaration of Helsinki.

Note that full information on the approval of the study protocol must also be provided in the manuscript.

Field-specific reporting

Please select the one below that is the best fit for your research. If you are not sure, read the appropriate sections before making your selection.

☐ Life sciences ☒ Behavioural & social sciences ☐ Ecological, evolutionary & environmental sciences

For a reference copy of the document with all sections, see nature.com/documents/nr-reporting-summary-flat.pdf

Behavioural & social sciences study design

All studies must disclose on these points even when the disclosure is negative.

Study description	The fMRI experiment employed a within-subjects, sham-controlled experimental design. The behavioural experiment employed a within-subjects, sham-controlled, crossover design. The order of the stimulation conditions was counterbalanced across subjects using a balanced Latin square. Quantitative data included physiological measurements and task performance metrics. In addition we collected qualitative perceptions of brain stimulation.
Research sample	The research sample for the ex-vivo experiment included one male cadaver (93 years old) with no known brain disorder provided by the “service des corps donnés à la science” by Aix Marseille Université. Twenty-two healthy volunteers took part in the fMRI experiment, two participants were excluded, one because of technical difficulties with the MRI scanner (no images were collected) and another due to excessive movement in the scanner. Thus, the final cohort for this experiment was composed of twenty subjects (11 females, age range: 20 to 54 years old, mean age 27.1 ± 7.6 SD, 19 right-handed and 1 left-handed). Twenty-one healthy volunteers took part in the behavioural experiment (10 females, age range: 19 to 30 years old, mean age 22.7 ± 3.2 SD, all right-handed). All volunteers were educated to degree level or above with no self-reported history of neurological or psychiatric illness.

Inclusion criteria for in-vivo experiments included healthy female and male participants aged 18-55 years that could read and understand the instructions. Exclusion criteria included any metal implants in the head or neurosurgical procedure, history of seizure, any active neurological or psychiatric conditions, active frequent migraines, any head injury that lead to a loss of consciousness/ concussion in the last 12 months, psychoactive medication and symptoms of COVID-19 (for the behavioural study). Our population was recruited based on convenience sampling and includes a high proportion of university students (not representative of the general population). No statistical methods were used to predetermine sample sizes, but our sample sizes are similar to those reported in previous publications.

Sampling strategy

Sampling procedure included convenience sampling. Sample size for the fMRI experiment was based on previous studies showing modulation of BOLD signal during simultaneous transcranial electrical stimulation and fMRI using short stimulation durations, employing an ON/OFF design to minimise build-up while maximising signal-to-noise-ratio to assess physiological responses (Violante et al., eLife 2017; Li, L.M., et al. Hum Brain Mapp 2019). The behavioural study followed a similar sample size, but included longer periods of stimulation and experimental trials, a protocol designed to probe behavioural effects of stimulation (Booth, S.J. et al, Cortex 2022). Sample sizes of approximately 20 participants have shown to be sufficient to detect significant effects in both experimental designs with 80% power at p = 0.05 significance level.

Data collection

sEEG data was collected using RHS Stim/Recording Controller (Intan Technologies). Behavioural data was collected using Psychtoolbox for Matlab (https://gitlab.eps.surrey.ac.uk/nemo/facenametask). MRI data was collected using a 3T Siemens Verio. Perceptual sensations associated with stimulation were collected electronically using Excel or Adobe Acrobat Reader. The study was single-blinded. The researcher was in the same room as the participant for the behavioural experiment.
The Face-Name task was chosen based on a strong body of evidence demonstrating that face-name associations are dependent on hippocampal function and elicit bilateral hippocampal activations in healthy subjects. Faces were retrieved from the Chicago Face Database v.2.0.368 and names from the Office for National Statistics (Baby Names, England and Wales, 1996; which corresponds to the dataset closest to the mean age for the faces in the Chicago Face Database, mean age = 28 years old). We selected names that had between 5 and 7 letters. Names present in both female and male lists were removed (e.g. Charlie) and if the same name was present with a different spelling (e.g. Elliot and Elliott) only the one with the highest frequency was kept. The task was composed of 9 blocks in the fMRI experiment and 12 blocks in the behavioural experiment, each containing 16 unique face-name pairs of different ethnicities (4 black female, 4 black male, 4 white female and 4 white male per block; all with neutral facial expressions). The task was composed of an encoding and a recall stage. During the encoding stage each face-name pair was displayed for 2 s. Faces were displayed in the centre of the screen with the name underneath. Participants were instructed to read the name underneath the faces and try to learn each face-name pair. This was followed by either a delay period (16 s) where a fixation cross was present in the centre of the screen in the fMRI experiment, or a distractor task (40 s) where participants made odd/even judgments for random integers ranging from 1 to 99 in the behavioural experiment. In the recall stage, participants were shown each face with 5 names underneath: the target name, two distractor names (i.e., names that were not present in the block), and two foil names (i.e., names that were present in the block but associated with a different face) - target and distractor names were selected to have a similar name frequency. For the recall stage, participants were instructed to respond as quickly and as accurately as possible. There was a time limit (20 s in the fMRI experiment and 8 s in the behavioural experiment) to select each name and to rate the confidence level (5 s in the fMRI experiment and 3 s in the behavioural experiment). The order of the blocks was kept constant across participants, but the order of the face-name pairs was pseudo-randomised across participants, such that no more than three faces of the same type appeared in a row. The order of face recall was randomised across participants, and the last two encoding trials were never presented at the beginning of the recall. The position of the names in the recall stage was also randomised. This was followed by a confidence rating, in which participants were asked to rate how confident they were in their selection from 1 to 4 (1 being not confident at all and 4 extremely confident). For the recall stage, participants were instructed to respond as quickly and as accurately as possible. There was a time limit (20 s in the fMRI experiment and 8 s in the behavioural experiment) to select each name and to rate the confidence level (5 s in the fMRI experiment and 3 s in the behavioural experiment).The total duration of the fMRI acquisition was on average 26.8 min (range 19.7 - 38.7 min). The duration of the task for the behavioural experiment was on average 34.3 min (range 28.5 - 42.1 min).

Timing

Data for the fMRI experiment was collected between June and October 2019. Data for the behavioural experiment was collected between January and February 2022. The gap between collection periods occurred during the COVID-19 pandemic for periods when the research facilities were closed or studies with human populations were limited.

Data exclusions

Two participants were excluded from the fMRI experiment. One because of technical difficulties with the MRI scanner (no images were collected) and another due to excessive movement in the scanner. Criterion for excessive motion was DVARS > 0.5 in more than 20% of the volumes.

Non-participation

No drop-outs.

Randomization

Within-subjects experiments. The order of the stimulation conditions was counterbalanced across subjects using a balanced Latin square.

Reporting for specific materials, systems and methods

We require information from authors about some types of materials, experimental systems and methods used in many studies. Here, indicate whether each material, system or method listed is relevant to your study. If you are not sure if a list item applies to your research, read the appropriate section before selecting a response.

Materials & experimental systems

n/a	Involved in the study
☒	☐ Antibodies
☒	☐ Eukaryotic cell lines
☒	☐ Palaeontology and archaeology
☒	☐ Animals and other organisms
☒	☐ Clinical data
☒	☐ Dual use research of concern

Methods

n/a	Involved in the study
☒	☐ ChIP-seq
☒	☐ Flow cytometry
☐	☒ MRI-based neuroimaging

Magnetic resonance imaging

Experimental design

Design type: Task, block design

Design specifications: The task was composed of 9 blocks of task in the fMRI experiment. Each task block was composed of an encode and a recall and confidence block. Encode blocks lasted for 32 s and contained 16 unique face-name pairs displayed for 2 s. Recall blocks were composed of each of the 16 faces paired with 5 possible matching names. Participants had a maximum of 20 s to provide a response. After each recall trial they were asked to provide their confidence level, by selecting a number from 1 to 4 with a maximum period of 5 s. Responses were thus self-paced and the duration of the recall blocks varied per participant. Between each encode and recall block there was a 16 s fixation cross. An equivalent period of 16 s with a fixation cross was presented before each encode block and at the beginning and end of the task. The fixation periods served as baseline. Stimulation (sham, TI 1:1 and TI 1:3) were delivered during the encode blocks. The stimulation began with a 5 s ramp-up and ended with a 5 s ramp-down.

Behavioral performance measures: Three main variables of interest were analysed, i.e., response type, reaction time for name selection and confidence level (reaction times for confidence were also recorded but not analysed). We first assessed accuracy across the whole task (correct vs incorrect associations) to check whether any participant had an overall performance below chance (20%), which would exclude them from further analyses. All participants were above chance (mean = 49.97%, SD = 9.77%, range 32.64 – 70.83 %).

Acquisition

Imaging type(s): Structural and functional images were acquired.

Field strength: 3 T

Sequence & imaging parameters: Structural: T1-weighted structural images were acquired using a magnetization-prepared rapid gradient-echo (MP-RAGE) sequence, 1 mm3 isotropic voxel, repetition time (TR) 2.3 s, echo time (TE) 2.98 ms, inversion time 900 ms, flip angle (FA) 9°, field of view 256 × 256 mm, 256 × 256 matrix, 160 slices, GRAPPA acceleration factor = 2.
Functional: T2*-weighted gradient-echo EPI sequence, 3 mm3 isotropic voxel, TR 2 s, TE 30 ms, FA = 80°, field of view 192 × 192 × 105 mm, 35 slices, GRAPPA acceleration factor = 2, AP-PC orientation. A total of 804 volumes were acquired on average (range: 592 – 1162), times varied depending on how long participants took on the recall stage of the task (see Design specifications above).

Area of acquisition: Whole-brain

Diffusion MRI: ☐ Used ☒ Not used

Preprocessing

Preprocessing software: Data were pre-processed using FMRIB Software Library (FSL version 6.0.1, FMRIB's Software Library, www.fmrib.ox.ac.uk/fsl). Functional data were pre-processed using the FMRI Expert Analysis Tool (FEAT), including motion correction using MCFLIRT, distortion correction using fieldmap images prepared from fsl_prepare_fieldmap, slice-time correction using Slicetimer, smoothing with a 3D Gaussian kernel (8 mm full-width at half maximum, FWHM) and high-pass filtered at a cut-off of 0.008 Hz. Skull stripping was performed using FSL's BET. Anatomical data was also processed using the SimNIBS framework (version 3.262), employing the 'headreco' pipeline. Hippocampal masks were defined based on the segmentation of the whole hippocampi performed for each subject using the pipeline for automated hippocampal subfield segmentation in FreeSurfer (version 6.0.0).

Normalization: Registration to high resolution structural and standard space images was carried out using FLIRT in FEAT using the default parameters (BBR for registration to structural image and 12 DOF to standard space). FLIRT (FMRIB's Linear Image Registration Tool) is a fully automated robust and accurate tool for linear (affine) intra- and inter-modal brain image registration.

Normalization template: MNI152

Noise and artifact removal: Gradient distortion correction and head-motion correction (MCFLIRT) using twenty-four motion parameters (six motion

Noise and artifact removal

parameters - translation and rotation in three directions, the square of the six motion parameters and their temporal derivatives).

Volume censoring

FSL motion outliers using DVARS (the spatial root mean square of the data after temporal differencing). Criterion for excessive motion was DVARS > 0.5 in more than 20% of the volumes. One subject was excluded based on this. For all other participants the GLMs included a regressor with volume outliers identified by DVARS to model out volumes (i.e., scrubbing) with extensive motion.

Statistical modeling & inference

Model type and settings

A standard mass univariate analysis was performed using FSL FEAT. Data was modelled using three different general linear models (GLMs). In addition to the explanatory variables (EVs) of interest (described below), all GLMs included as nuisance regressors twenty-four motion parameters (six motion parameters - translation and rotation in three directions, the square of the six motion parameters and their temporal derivatives) and a regressor with volume outliers identified by DVARS to model out volumes (i.e., scrubbing) with extensive motion.

Effect(s) tested

NB: All the effects were tested between stimulation conditions.

The first GLM was used to analyse univariate BOLD effects during encode and recall periods of the task and included 3 EVS for encode and 3 EVS for recall (one EV per stimulation condition and task stage) and their first temporal derivatives. Regressors were created by convolving a boxcar kernel with a canonical double-gamma hemodynamic response function.
The second GLM analysed univariate BOLD effects for correct and incorrect trials during encode and recall periods. This model included 12 EVs (one for correct and another for incorrect trials for encode and recall periods per stimulation condition), 3 EVs for the confidence intervals (one per stimulation condition) and their first temporal derivatives. Regressors were created by convolving a boxcar kernel with a canonical double-gamma hemodynamic response function.
The third set of GLMs, used to assess functional connectivity. We used a generalised psychophysiological interaction (gPPI) method to quantify the effective connectivity for the contrast correct > incorrect, using the Ant, Mid and Post regions of the left hippocampus as seeds and the AT and PM network as targets. The gPPI models included 25 EVs, describing physiological, psychological and PPI regressors. Physiological regressors were defined from the fMRI time-course extracted from seeds in the Ant, Mid and Post left hippocampus. The psychological regressors included those modelled for the second GLM. For each model (one per seed), the physiological term and the psychological term were used to create the PPI interaction terms.
Using the output of the first GLM we assessed the fMRI BOLD signal to the encode and recall periods of the task (contrasted against the baseline). Using the output of the second GLM, we measured BOLD response to correct and incorrect associations during the encode period. The contrast correct > incorrect was also used to extract connectivity values in the gPPI models described above.

Specify type of analysis: ☐ Whole brain ☒ ROI-based ☐ Both

Anatomical location(s)

1) the hippocampi - Hippocampal masks were defined based on the segmentation of the whole hippocampi performed for each subject using the pipeline for automated hippocampal subfield segmentation in FreeSurfer (version 6.0.0).

2) longitudinal parcellations of the hippocampi - hippocampal masks were normalised to MNI and split into thirds along the long axis of the hippocampus (posterior portion of the hippocampus: from Y= −40 to −30; mid-portion of the hippocampus: from Y= −29 to −19; anterior portion of the hippocampus: from Y= −18 to −4). The inverse normalization parameters were used to create subject specific parcellated ROIs and used in the subject space for fMRI analyses.

3) regions corresponding to the AT-PM networks - obtained from probabilistic atlases thresholded at 50%, including a medial temporal lobe atlas (https://neurovault.org/collections/3731/; 84) for parahippocampal cortex and precuneus, and the Harvard-Oxford cortical and subcortical atlases for all other regions.

4) regions corresponding to the cortical regions underneath the stimulation electrodes and between the stimulation electrodes in the left hemisphere - defined for each subject using the anatomical T1 images.
Left Hemisphere (3 ROIs): one ROI was placed underneath the anterior stimulating electrode e1 (i.e., ROI Crtx Ant) and a second ROI was placed underneath the posterior stimulating electrode e3 (i.e., ROI Crtx Post), the third ROI was placed in the middle between the electrodes (i.e., ROI Crtx Mid).
Right Hemisphere (2 ROIs): each ROI placed underneath the stimulating electrodes in the right hemisphere (Crtx Ant and Crtx Post).
All cortex ROIs were 10 mm spherical masks.

Statistic type for inference
(See Eklund et al. 2016)

The Eklund paper concerns traditional group-level random effect analyses. Our main analyses are performed within individuals and then the extracted responses are analyzed with conservative general / linear mixed effects models.

Additional voxelwise analyses within ROIs were performed using FSL's randomise tool with 5,000 permutations and family-wise error correction for multiple comparisons using threshold-free cluster enhancement (TFCE). All statistical maps were family-wise corrected and thresholded at $p<0.05$.

Whole-brain group-level maps for visualisation of task activity were thresholded using Gaussian Random Fields based cluster inference with an initial cluster-forming threshold of $Z>3.1$ and a family-wise error (FWE) corrected cluster-extent threshold of $p<0.05$.

Correction	N/A (key tests are performed across conditions for individualised ROIs)<br>For voxelwise within ROI analyses FWE was used.

Models & analysis

n/a	Involved in the study
☐	☒ Functional and/or effective connectivity
☒	☐ Graph analysis
☒	☐ Multivariate modeling or predictive analysis

Functional and/or effective connectivity	generalised psychophysiological interaction (gPPI) method
